# Supplementary material for: Design and Synthesis of (Z)-2-(Benzylamino)-5-benzylidenethiazol-4(5H)-one Derivatives as Tyrosinase Inhibitors and Their Anti-Melanogenic and Antioxidant Effects
Source: Molecules. 2023 Jan 14;28(2):848. doi: 10.3390/molecules28020848 (PMC9865752; doi:10.3390/molecules28020848)
Supplement: Supplementary file 1 [file molecules-28-00848-s001.zip › molecules-2117016-supplementary.pdf]

Supporting Information

For

Design and synthesis of (*Z*)-2-(benzylamino)-5-benzylidenethiazol-4(5*H*)-one derivatives as tyrosinase inhibitors and their anti-melanogenic and antioxidant effects

Jieun Lee 1,†, Yu Jung Park 1,†, Hee Jin Jung 2,† , Sultan Ullah 3, Dahye Yoon 1, Yeongmu Jeong 1, Ga Young Kim 1, Min Kyung Kang 1, Dongwan Kang 4, Yujin Park 4, Pusoon Chun 5, Hae Young Chung 2 and Hyung Ryong Moon 1,*

[1] Laboratory of Medicinal Chemistry, Department of Manufacturing Pharmacy, College of Pharmacy, Pusan National University, Busan 46241, Republic of Korea

[2] Department of Pharmacy, College of Pharmacy, Pusan National University, Busan 46241, Republic of Korea

[3] Department of Molecular Medicine, UF Scripps Biomedical Research, West Palm Beach, FL 33458, USA

[4] Department of Medicinal Chemistry, New Drug Development Center, Daegu-Gyeongbuk Medical Innovation Foundation, Daegu 41061, Republic of Korea

[5] College of Pharmacy and Inje Institute of Pharmaceutical Sciences and Research, Inje University, Gimhae 50834, Gyeongnam, Republic of Korea

* Correspondence: mhr108@pusan.ac.kr; Tel.: +82-51-510-2815; Fax: +82-51-513-6754

† These authors contributed equally to this work.

Contents

Figure S1. ^{1}H NMR spectrum of compound **15**……….……………………………………………..5

Figure S2. ^{13}C NMR spectrum of compound **15**……..……………………..……………………...6

Figure S3. LRMS (ESI+) spectrum of compound **15**.…………………..……………..……..…7

Figure S4. ^{1}H NMR spectrum of compound **16**…………..…………………………………………..8

Figure S5. ^{13}C NMR spectrum of compound **16**….………………...…..……………..………...9

Figure S6. LRMS (ESI+) spectrum of compound **16**……..……………………..……………..…10

Figure S7. ^{1}H NMR spectrum of compound **1**…….…………………………………..………….......11

Figure S8. ^{13}C NMR spectrum of compound **1**………..….………………...…..……………………...12

Figure S9. LRMS (ESI–) spectrum of compound **1**………………………………………………13

Figure S10. LRMS (ESI+) spectrum of compound **1**……………………………………..………14

Figure S11. ^{1}H NMR spectrum of compound **2**…..……..…………….…………………..……..…...15

Figure S12. ^{13}C NMR spectrum of compound **2**……………...………………......…………………16

Figure S13. LRMS (ESI–) spectrum of compound **2**………………..…………………..……..…....17

Figure S14. LRMS (ESI+) spectrum of compound **2**………………..…………..………..…..….....18

Figure S15. ^{1}H NMR spectrum of compound **3**……….……………………………....…..……......19

Figure S16. ^{13}C NMR spectrum of compound **3**…….……………………....………..………….20

Figure S17. LRMS (ESI–) spectrum of compound **3**……………….…………………..……..…....21

Figure S18. LRMS (ESI+) spectrum of compound **3**……………….…………………..…..…..…...22

Figure S19. ^{1}H NMR spectrum of compound **4**……..……………………………...………...…...23

Figure S20. ^{13}C NMR spectrum of compound **4**………...……………………………………….24

Figure S21. LRMS (ESI–) spectrum of compound **4**……………….…………………..……..…....25

Figure S22. LRMS (ESI+) spectrum of compound **4**……………….…………………..…..…..…...26

Figure S23. ^{1}H NMR spectrum of compound **5**……..……………………………...………...…..27

Figure S24. ^{13}C NMR spectrum of compound **5**……..…...……………………………………….28

Figure S25. LRMS (ESI–) spectrum of compound **5**……………….…………………..……..…....29

Figure S26. LRMS (ESI+) spectrum of compound **5**……………….…………………..…..…........30

Figure S27. ^{1}H NMR spectrum of compound **6**……..……………………………...………...…..31
Figure S28. ^{13}C NMR spectrum of compound **6**……..…...………………………………………32
Figure S29. LRMS (ESI+) spectrum of compound **6**……..………………..…..………………..…..33
Figure S30. ^{1}H NMR spectrum of compound **7**…….………………………………....………...…..34
Figure S31. ^{13}C NMR spectrum of compound **7**……..…...……………..……………………………35
Figure S32. LRMS (ESI–) spectrum of compound **7**……………..………………….……..…....36
Figure S33. LRMS (ESI+) spectrum of compound **7**……………..………………….…..…........37
Figure S34. ^{1}H NMR spectrum of compound **8**…….………………………………....………...…..38
Figure S35. ^{13}C NMR spectrum of compound **8**……..…...……………..……………………………39
Figure S36. Proton-coupled ^{13}C NMR spectrum of compound **8**……..…...……………..…………...40

Figure S37. LRMS (ESI+) spectrum of compound **8**……..…………………………………………41

Figure S38. HRMS (ESI+) spectrum of compound **8**……………………………………………..42

Figure S39. ^{1}H NMR spectrum of compound **9**……..………………………………...………...…..43

Figure S40. ^{13}C NMR spectrum of compound **9**……..…...……………..………………………….……44

Figure S41. LRMS (ESI+) spectrum of compound **9**……..…………………….………………….……45

Figure S42. ^{1}H NMR spectrum of compound **10**…..……………………………...………...……46

Figure S43. ^{13}C NMR spectrum of compound **10**……...…………….…………………………….…..47

Figure S44. LRMS (ESI–) spectrum of compound **10**……………..…………………..……..…......48

Figure S45. LRMS (ESI+) spectrum of compound **10**………………..………………….. …..…......49

Figure S46. ^{1}H NMR spectrum of compound **11**…..……………………………...………..…….50

Figure S47. ^{13}C NMR spectrum of compound **11**…………………………………………………………51

Figure S48. LRMS (ESI–) spectrum of compound **11**…………………………………………………52

Figure S49. LRMS (ESI+) spectrum of compound **11**…………………………………………………53

Figure S50. ^{1}H NMR spectrum of compound **12**…………………………………………………………54

Figure S51. ^{13}C NMR spectrum of compound **12**…………………………………………………………55

Figure S52. LRMS (ESI–) spectrum of compound **12**…………………………………………………56

Figure S53. LRMS (ESI+) spectrum of compound **12**…………………………………………………57

Figure S54. ^{1}H NMR spectrum of compound **13**…………………………………………………………58

Figure S55. ^{13}C NMR spectrum of compound **13**…………………………………………………………59

Figure S56. LRMS (ESI–) spectrum of compound **13**…………………………………………………60

Figure S57. LRMS (ESI+) spectrum of compound **13**……………..…………………..……...…....61

Figure S58. ^{1}H NMR spectrum of compound **14**……...……………..………………...…………..62

Figure S59. ^{13}C NMR spectrum of compound **14**…………………………..………………….......63

Figure S60. LRMS (ESI–) spectrum of compound **14**……………..…………………..……...….....64

Figure S61. LRMS (ESI+) spectrum of compound **14**………………..…………………..…..……......65.

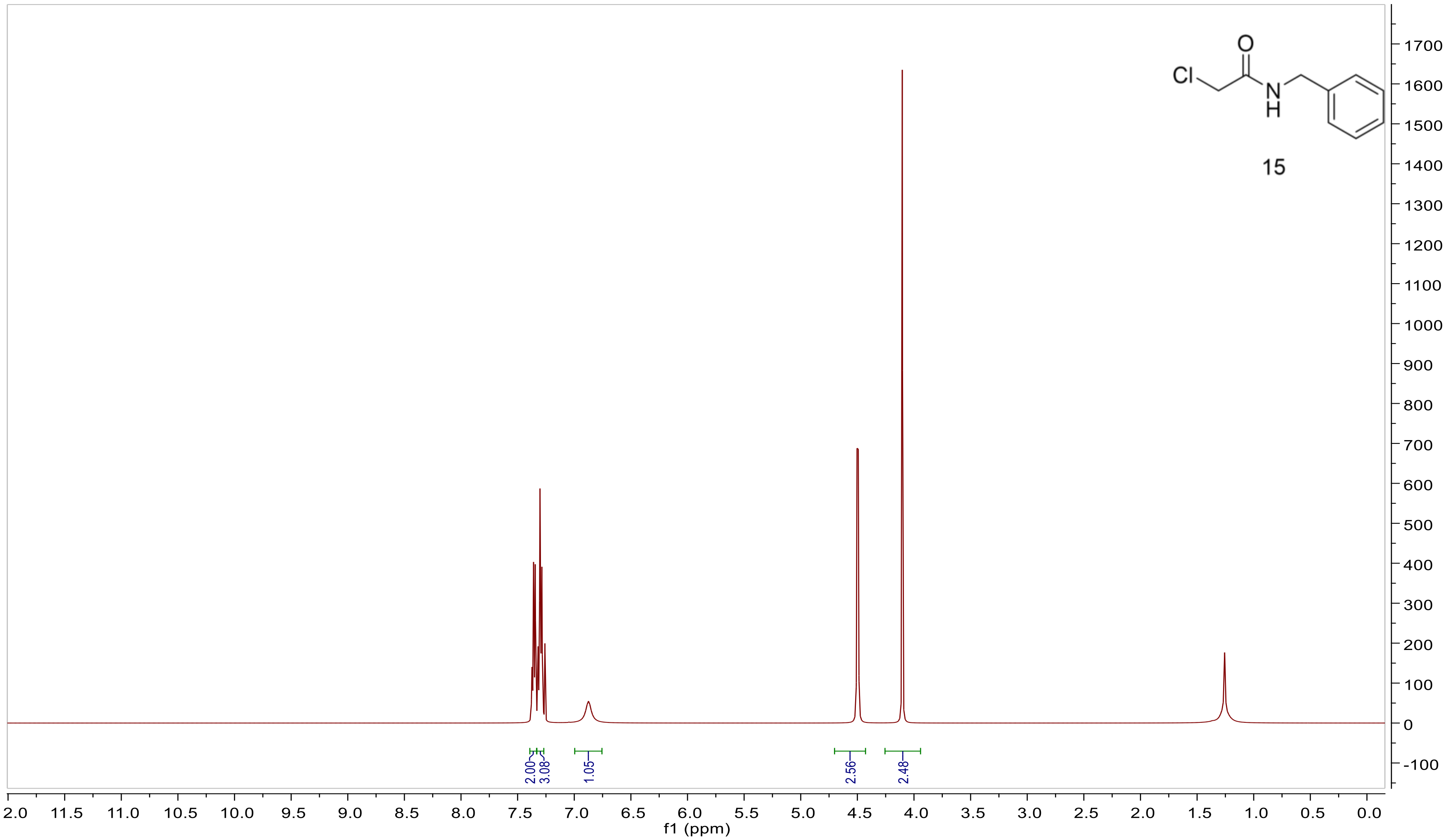

Figure S1. ^{1}H NMR spectrum of compound **15**

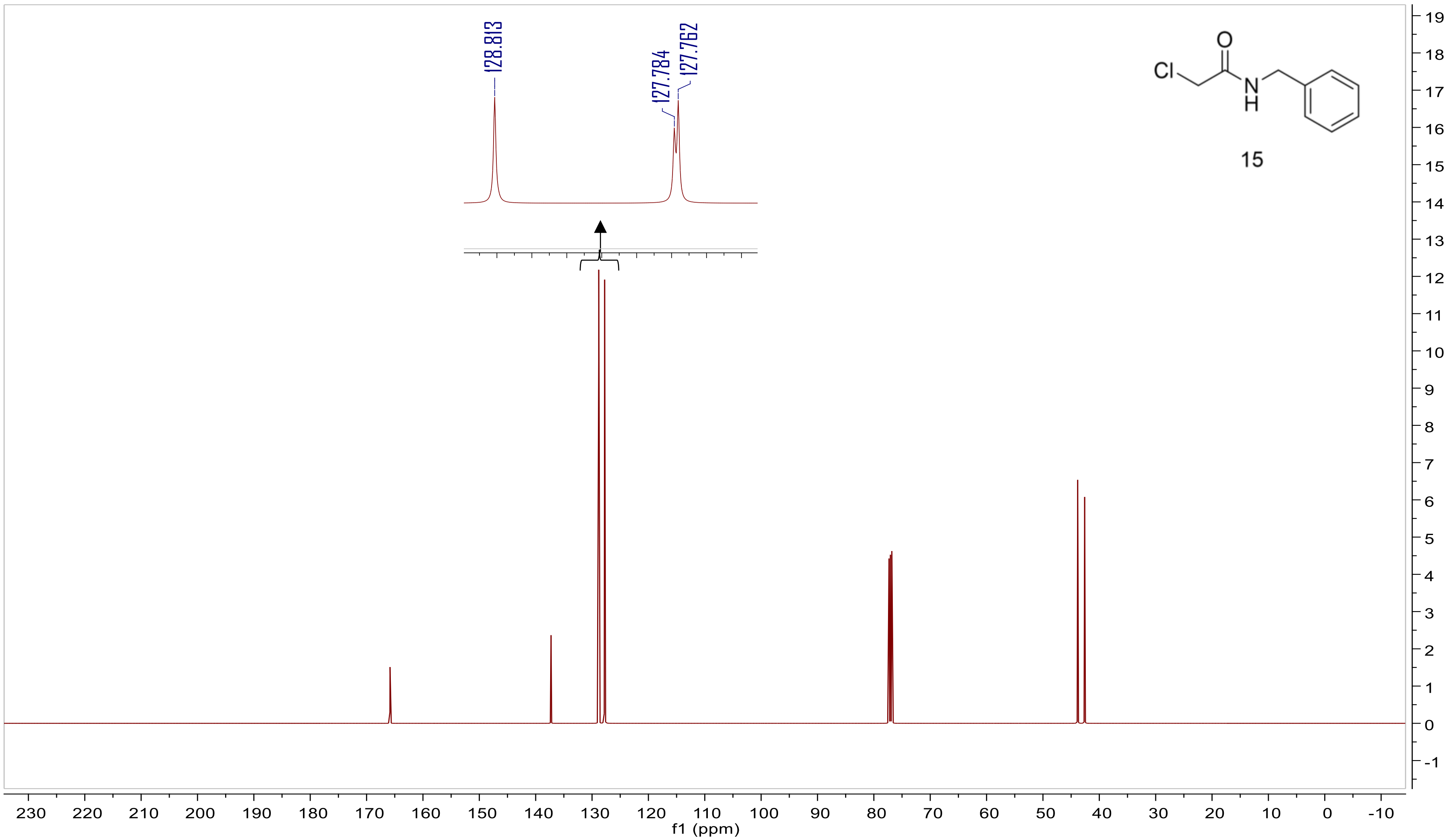

Figure S2. ^{13}C NMR spectrum of compound **15**

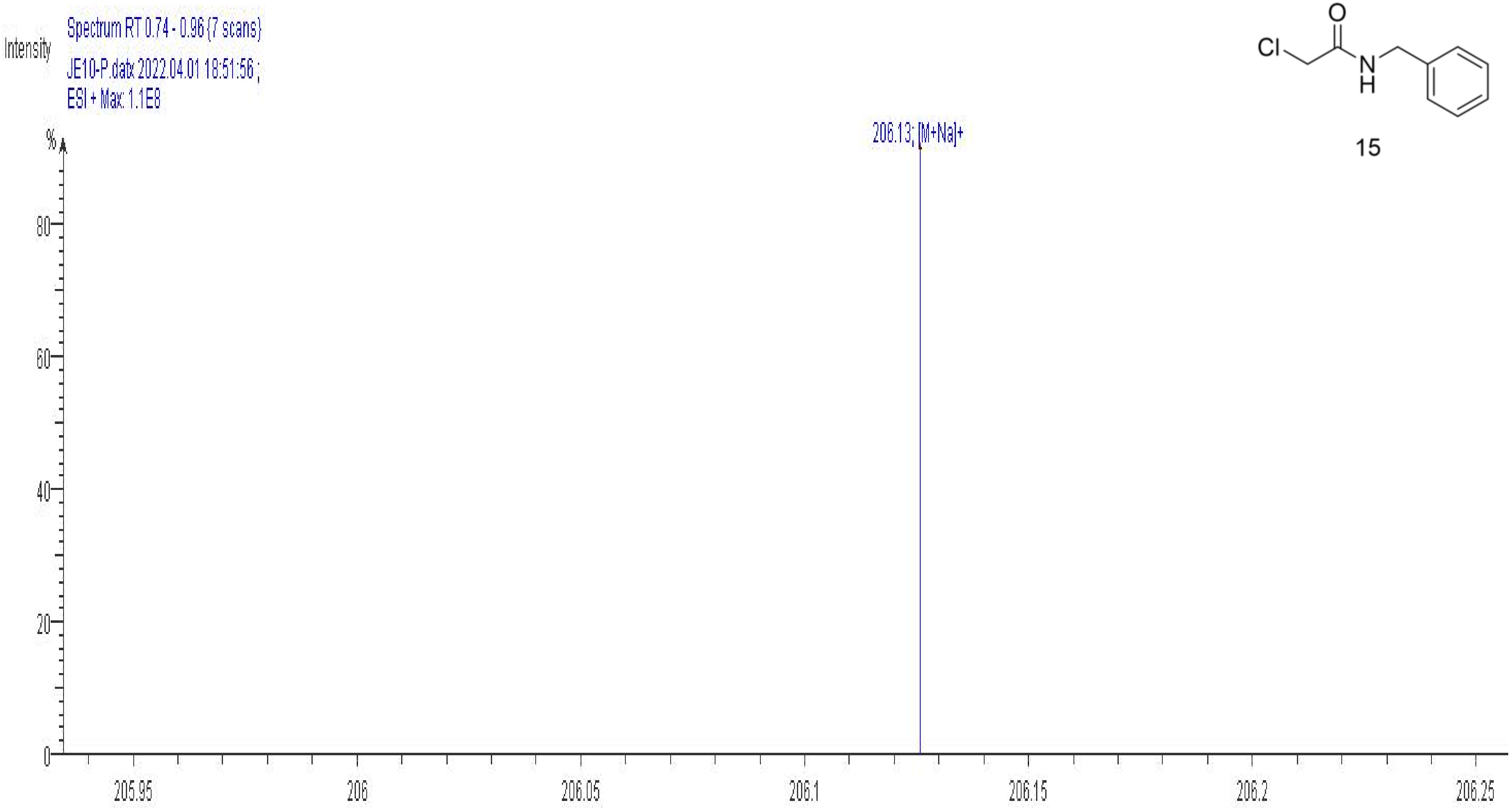

15

Figure S3. LRMS (ESI+) spectrum of compound **15**

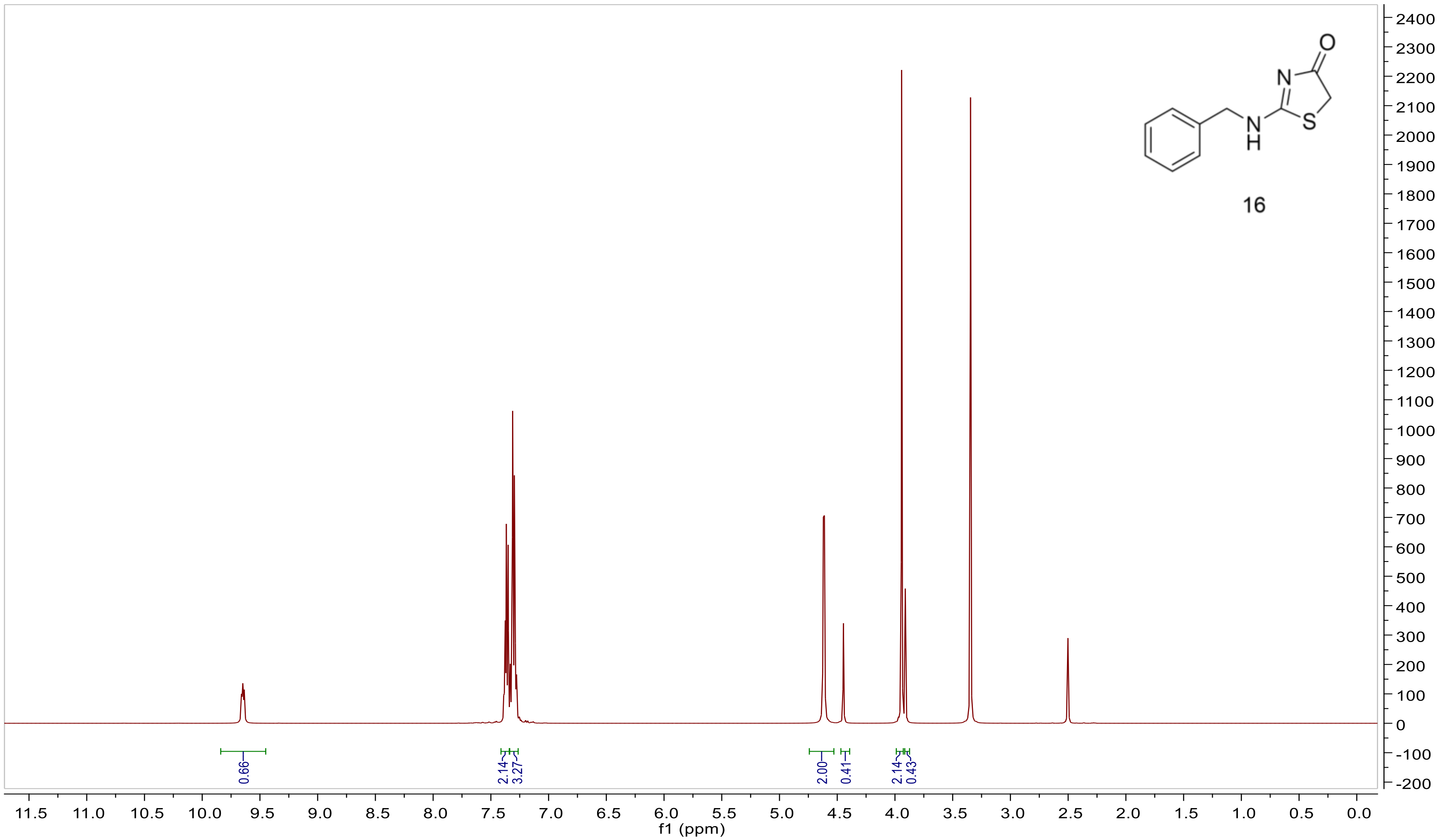

Figure S4. ^{1}H NMR spectrum of compound **16**

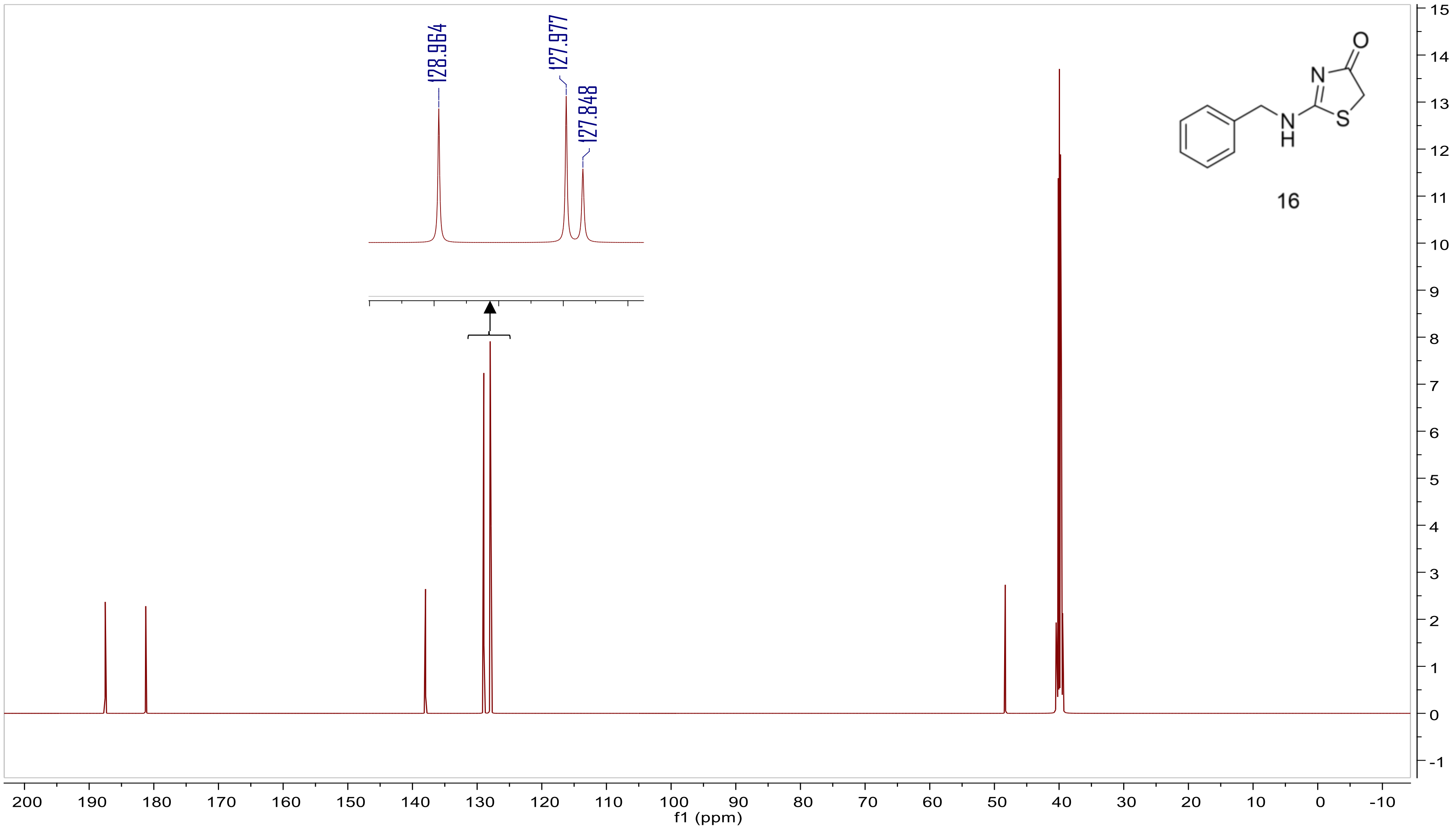

Figure S5. ^{13}C NMR spectrum of compound **16**

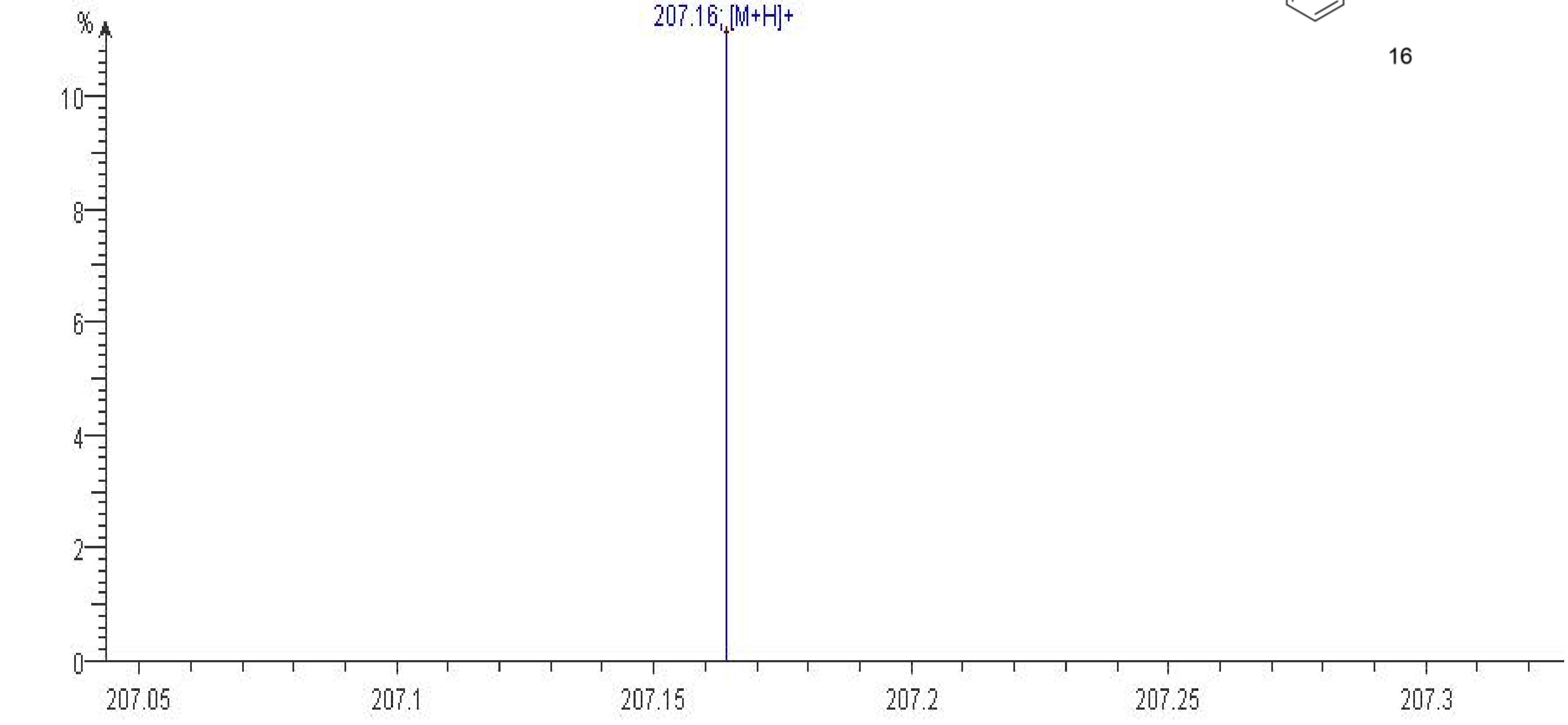

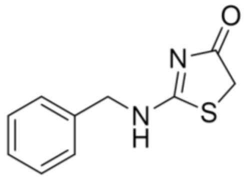

16

Figure S6. LRMS (ESI+) spectrum of compound **16**

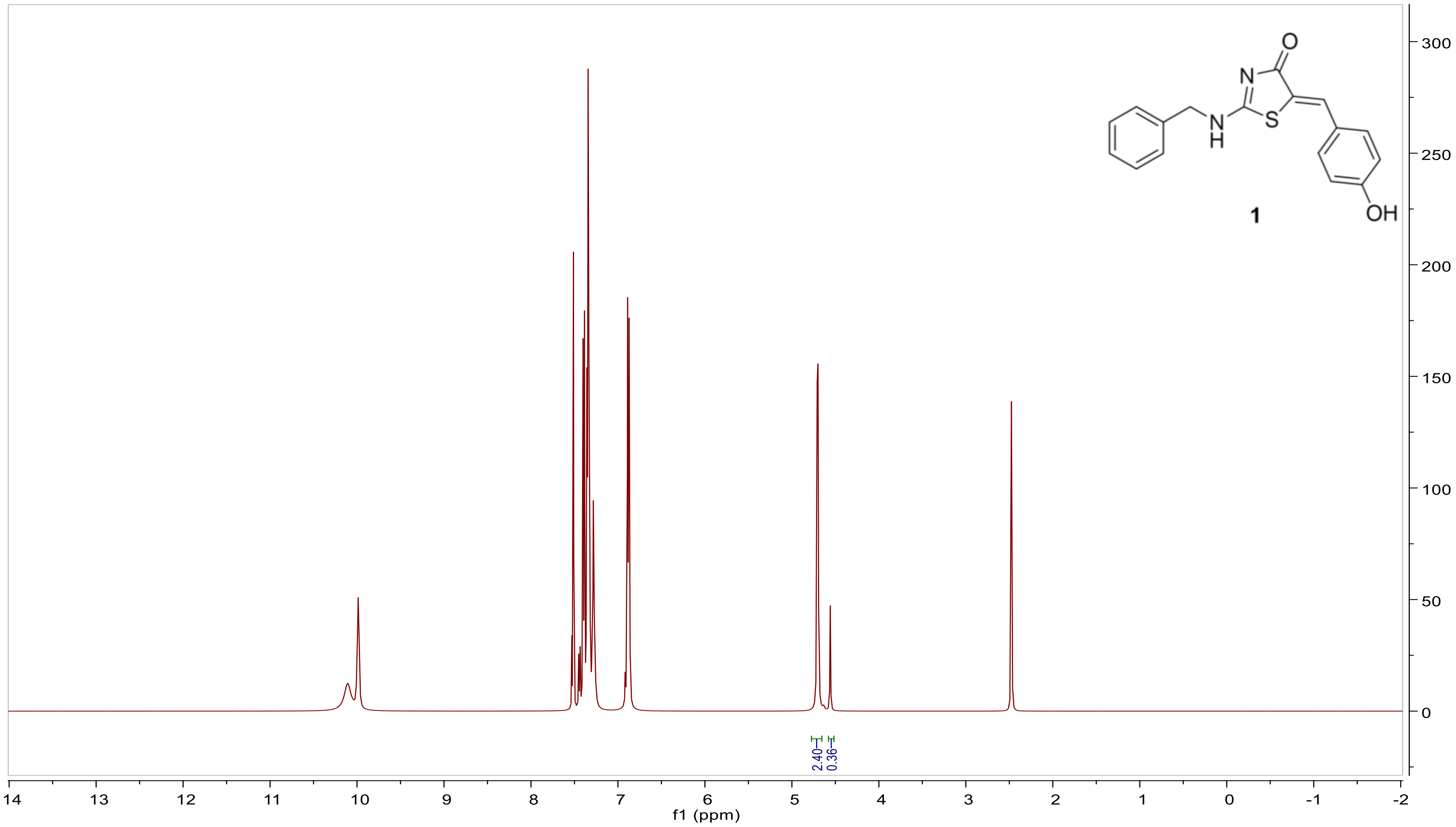

Figure S7. ^{1}H NMR spectrum of compound **1**

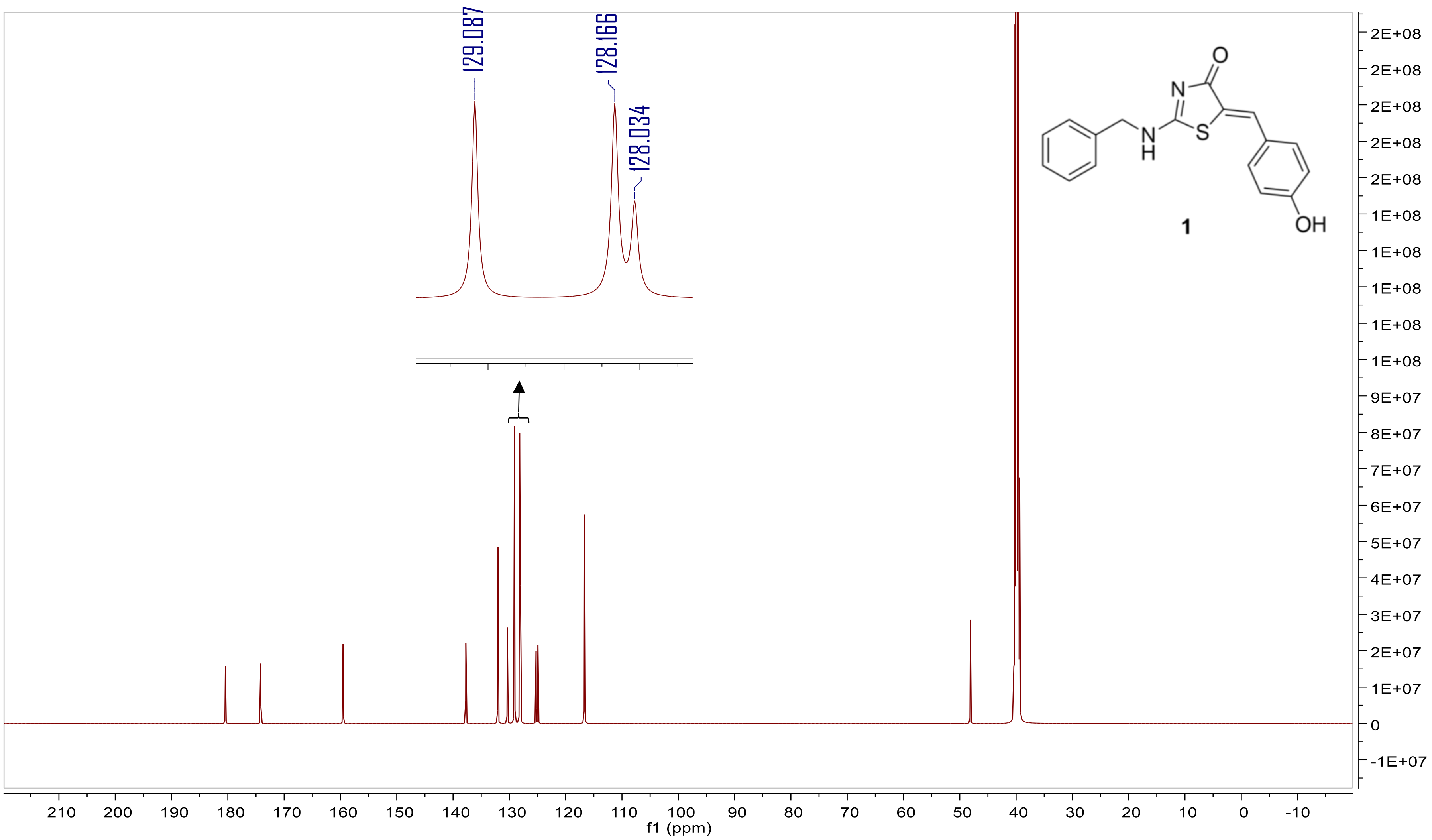

Figure S8. ^{13}C NMR spectrum of compound **1**

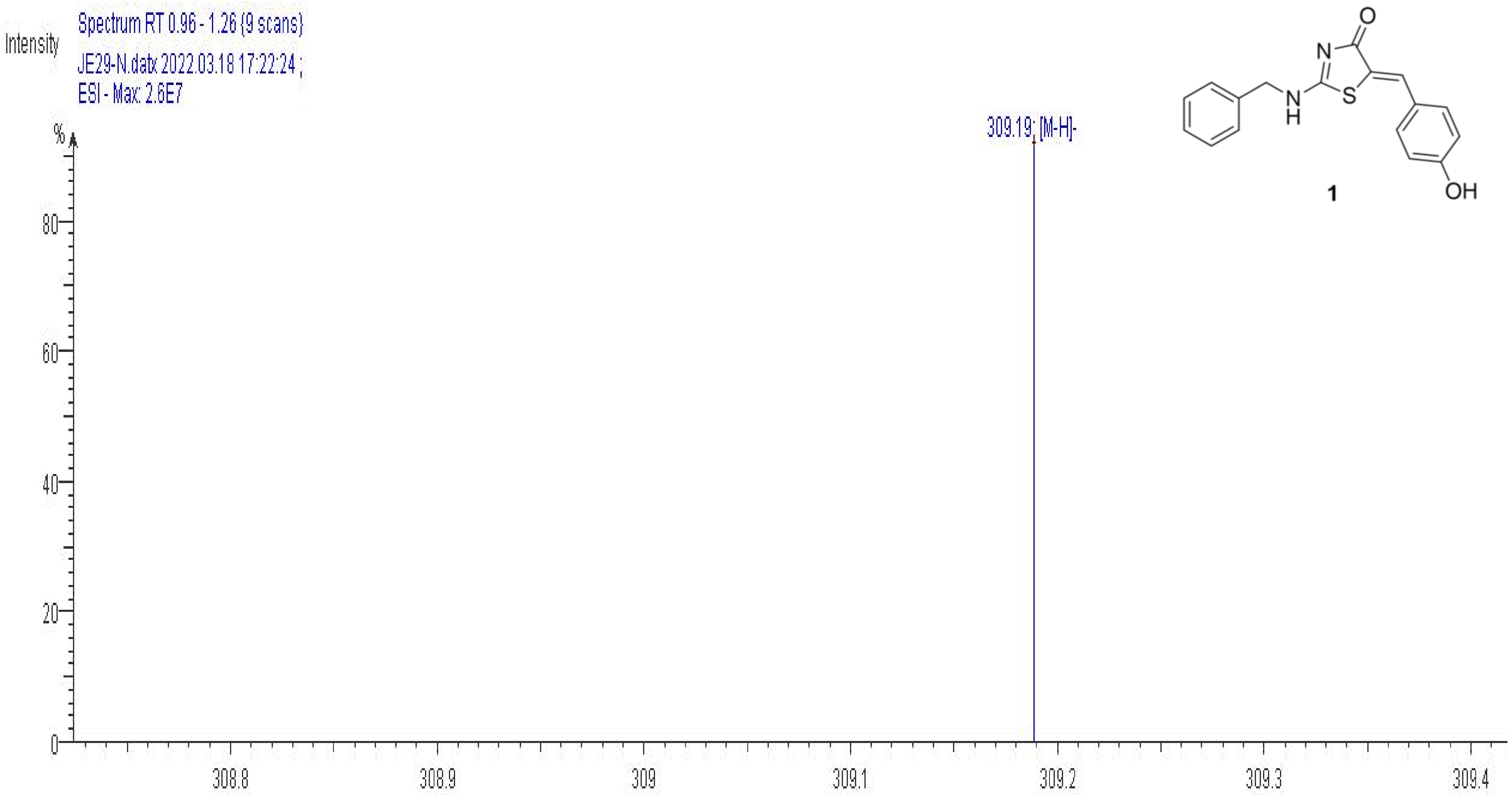

Figure S9. LRMS (ESI–) spectrum of compound **1**

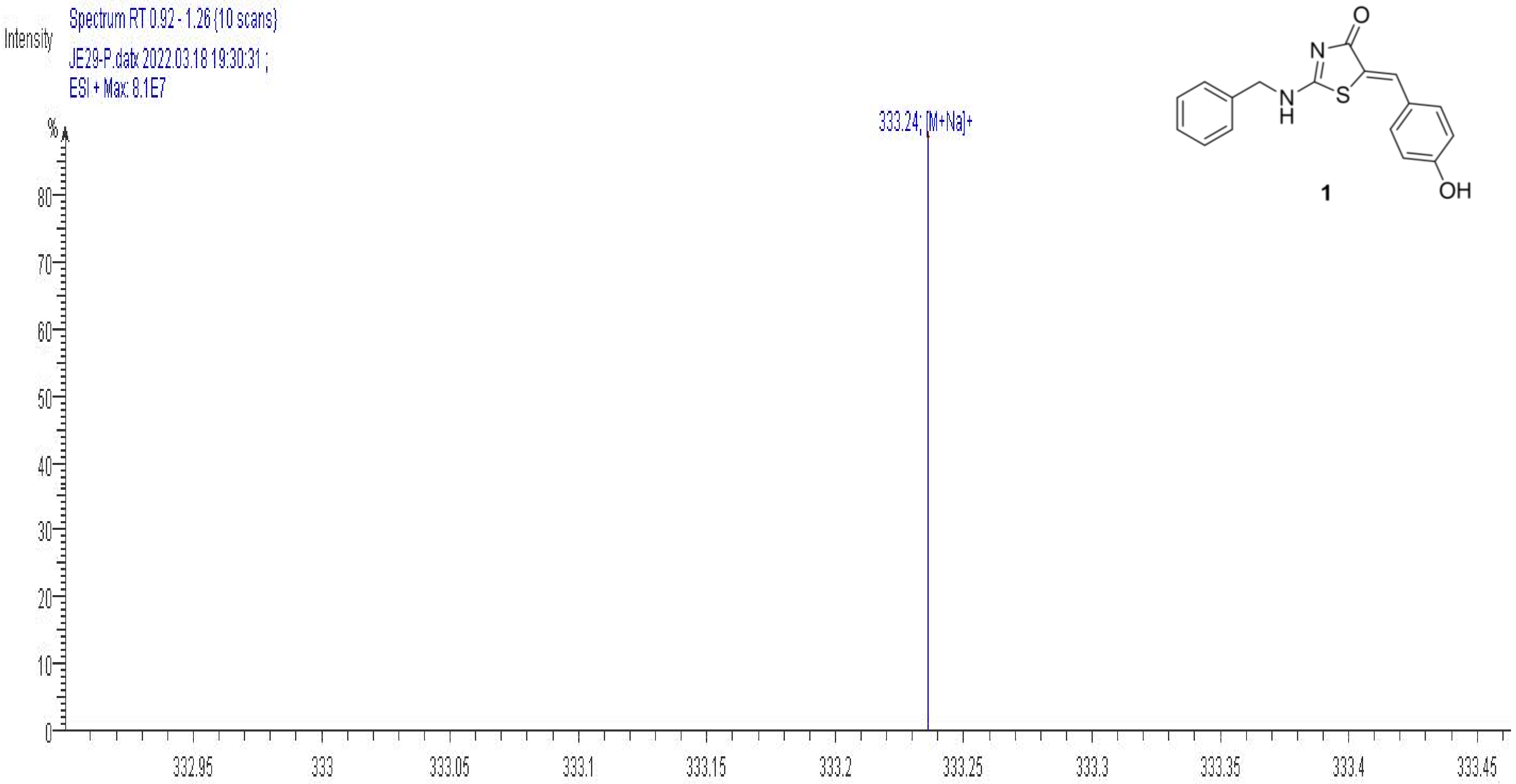

Figure S10. LRMS (ESI+) spectrum of compound **1**

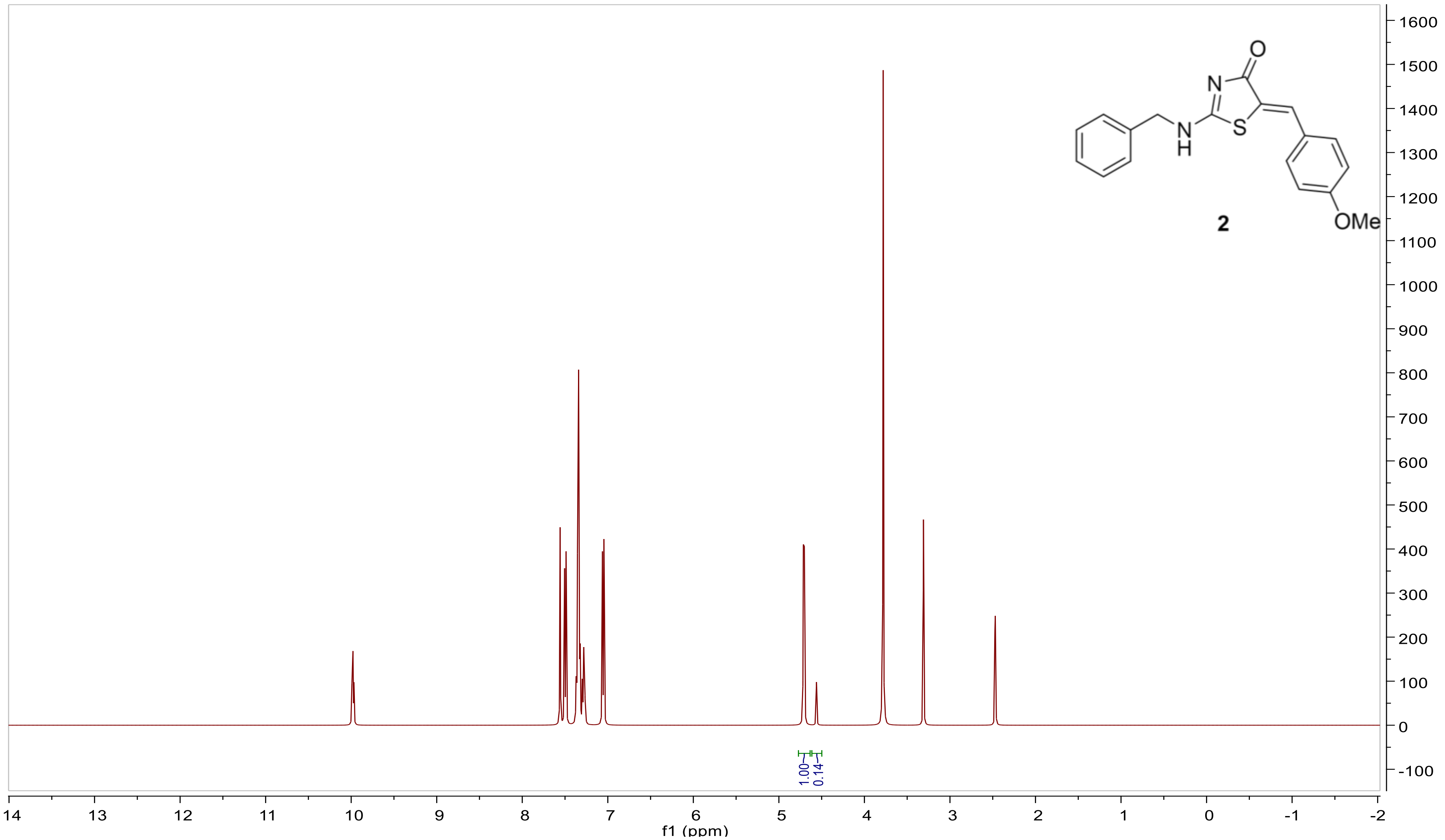

Figure S11. ^{1}H NMR spectrum of compound **2**

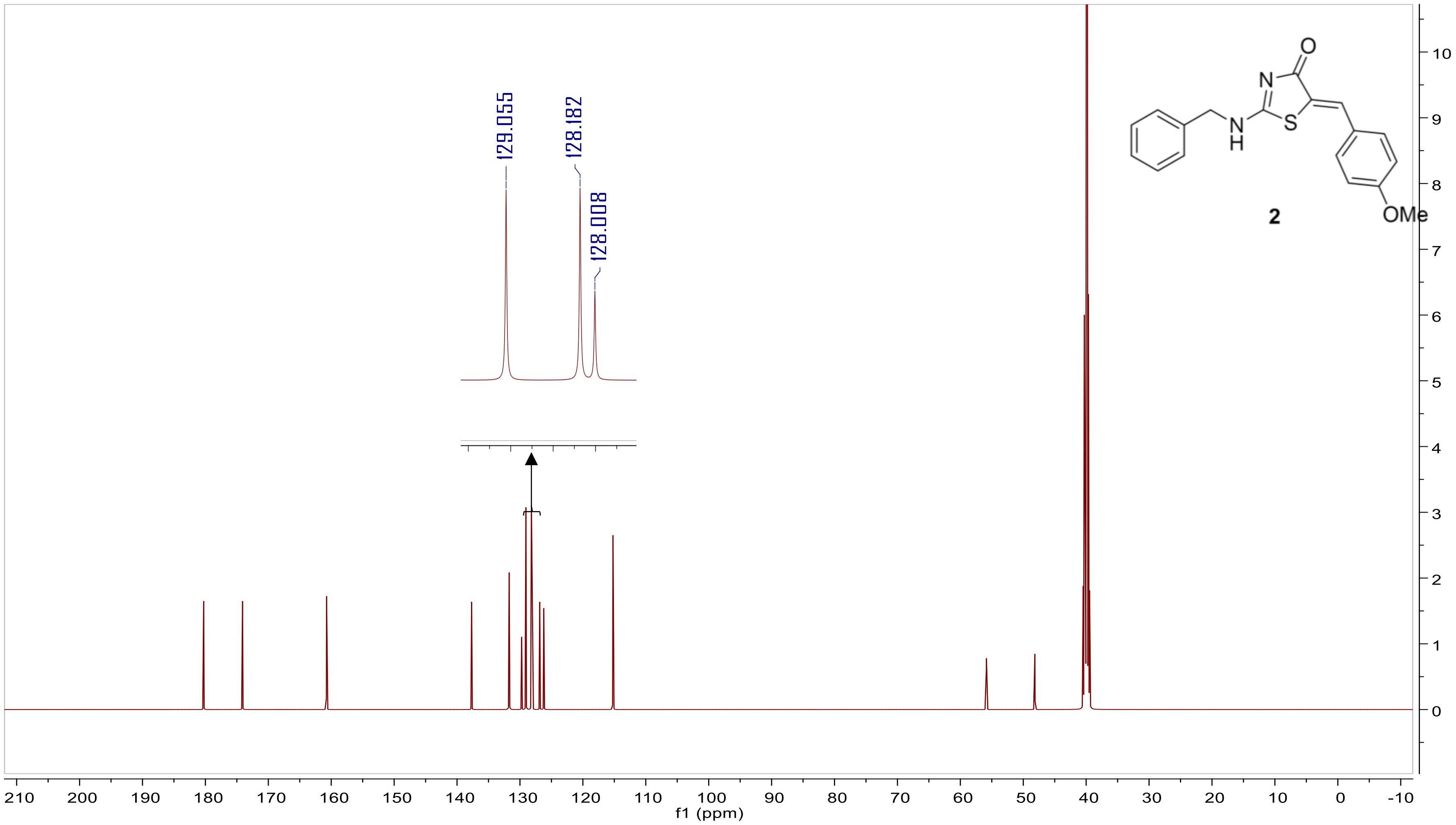

Figure S12. ^{13}C NMR spectrum of compound **2**

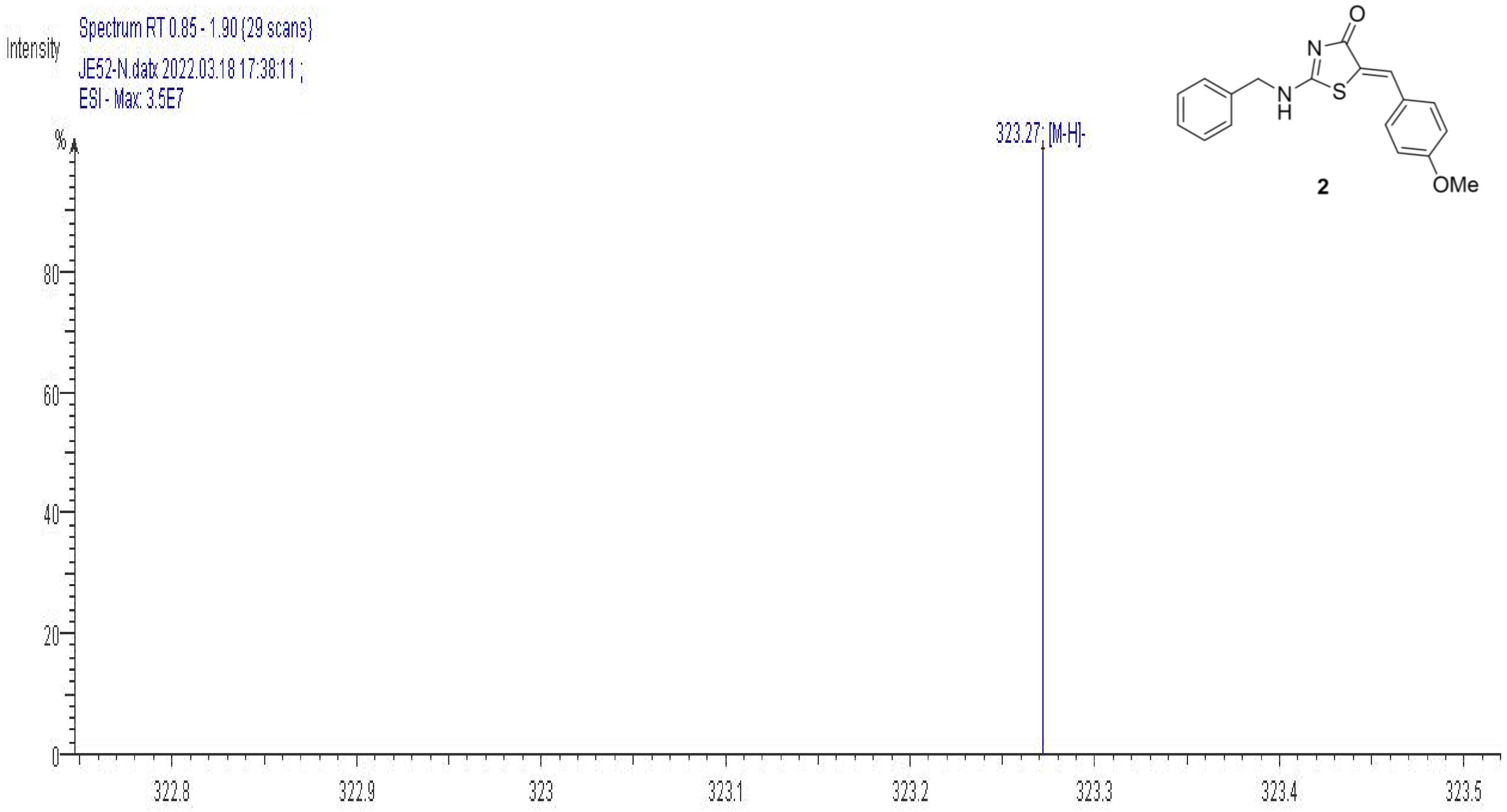

Figure S13. LRMS (ESI–) spectrum of compound **2**

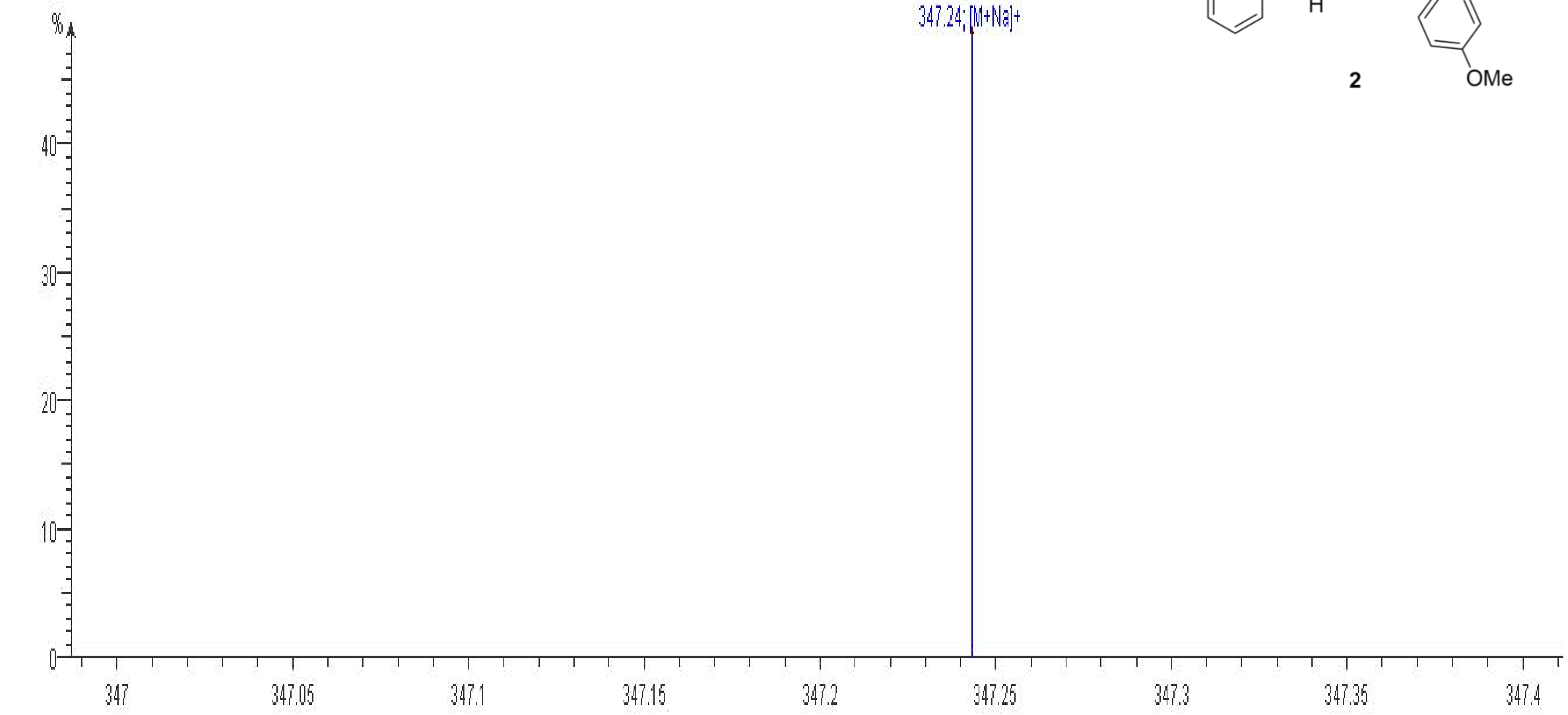

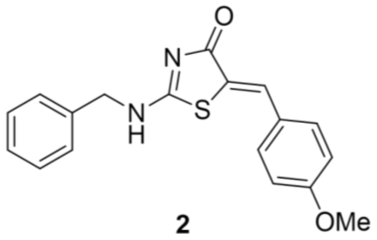

2

Figure S14. LRMS (ESI+) spectrum of compound **2**

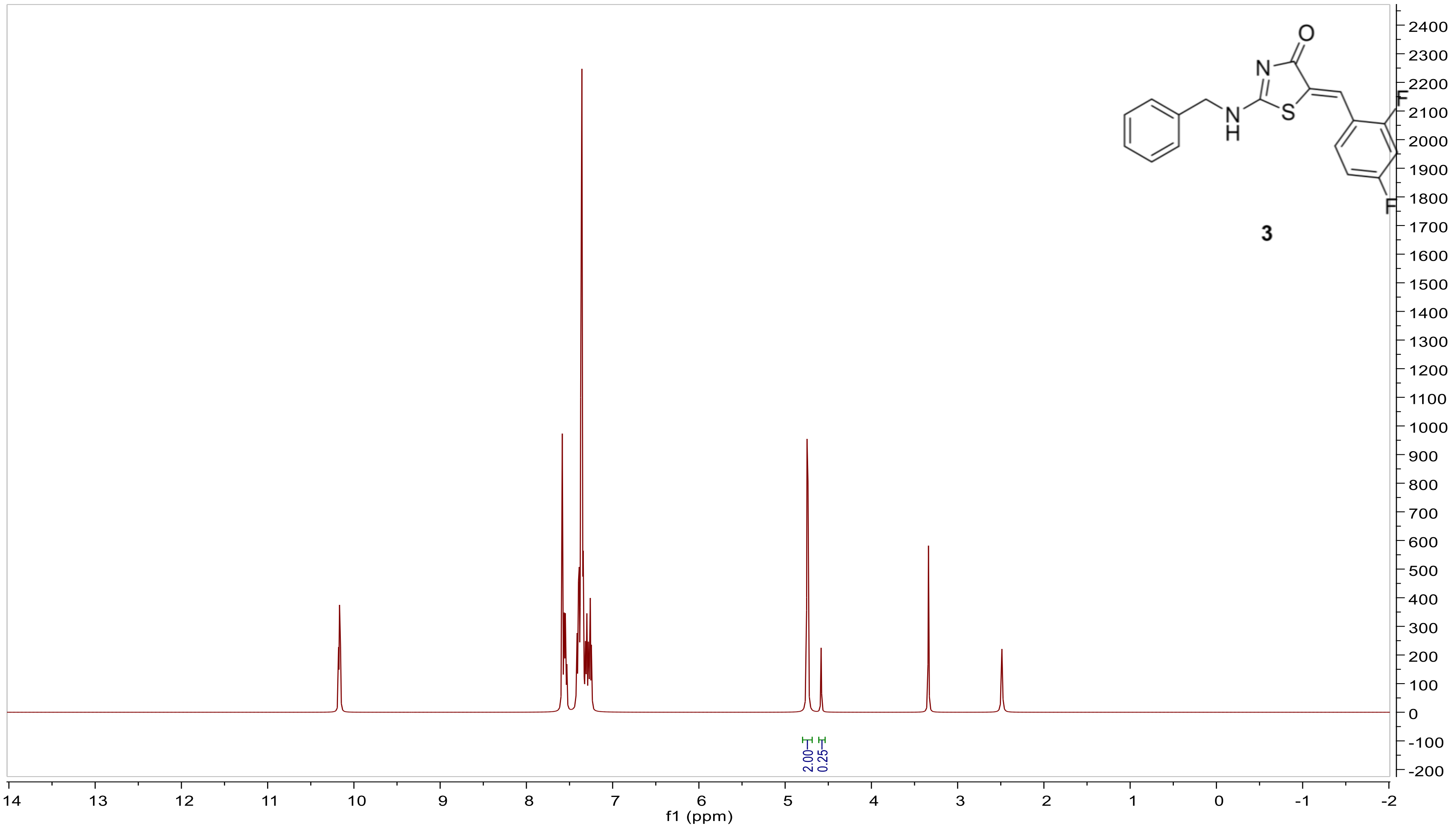

Figure S15. ^{1}H NMR spectrum of compound **3**

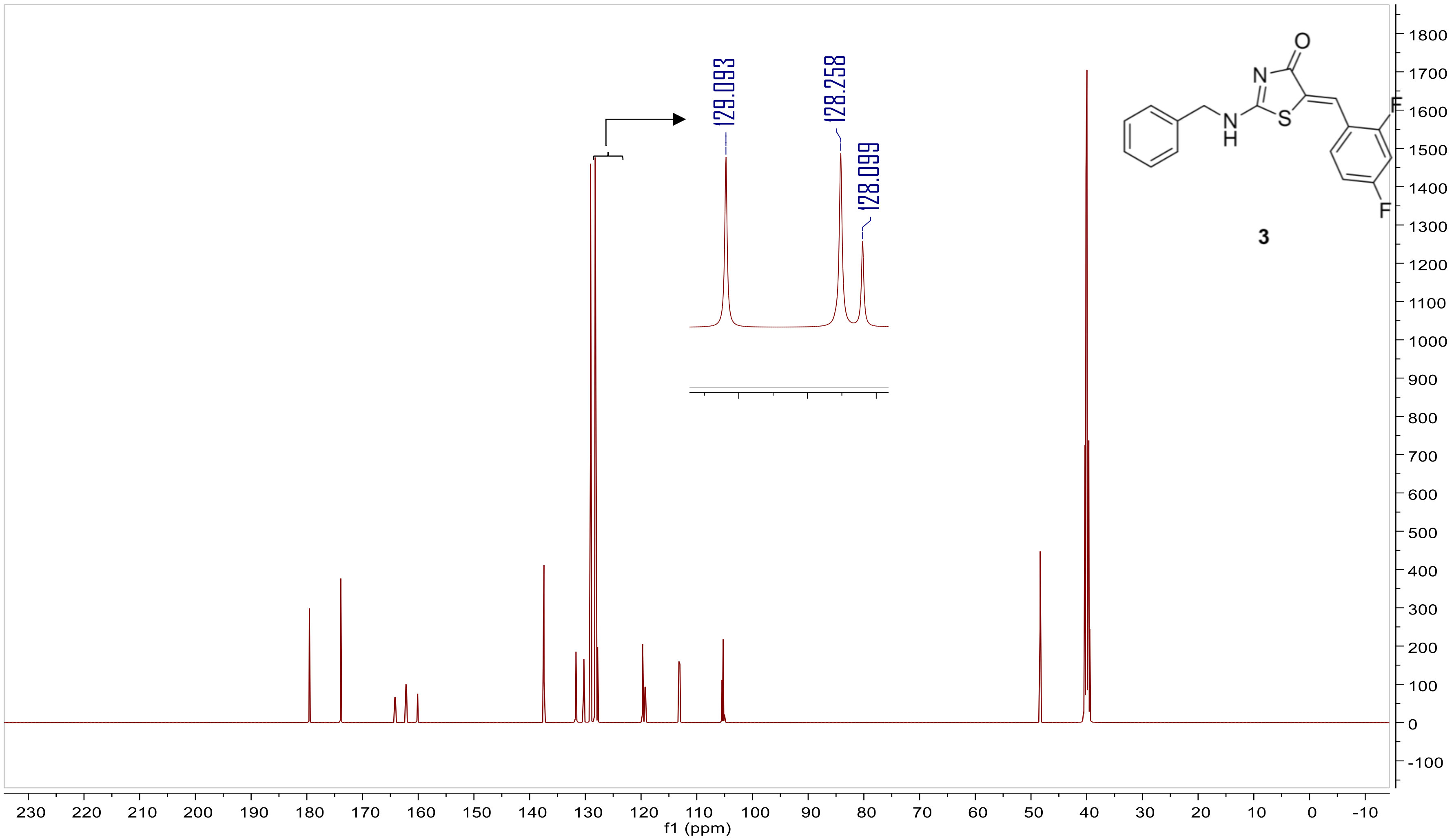

Figure S16. ^{13}C NMR spectrum of compound **3**

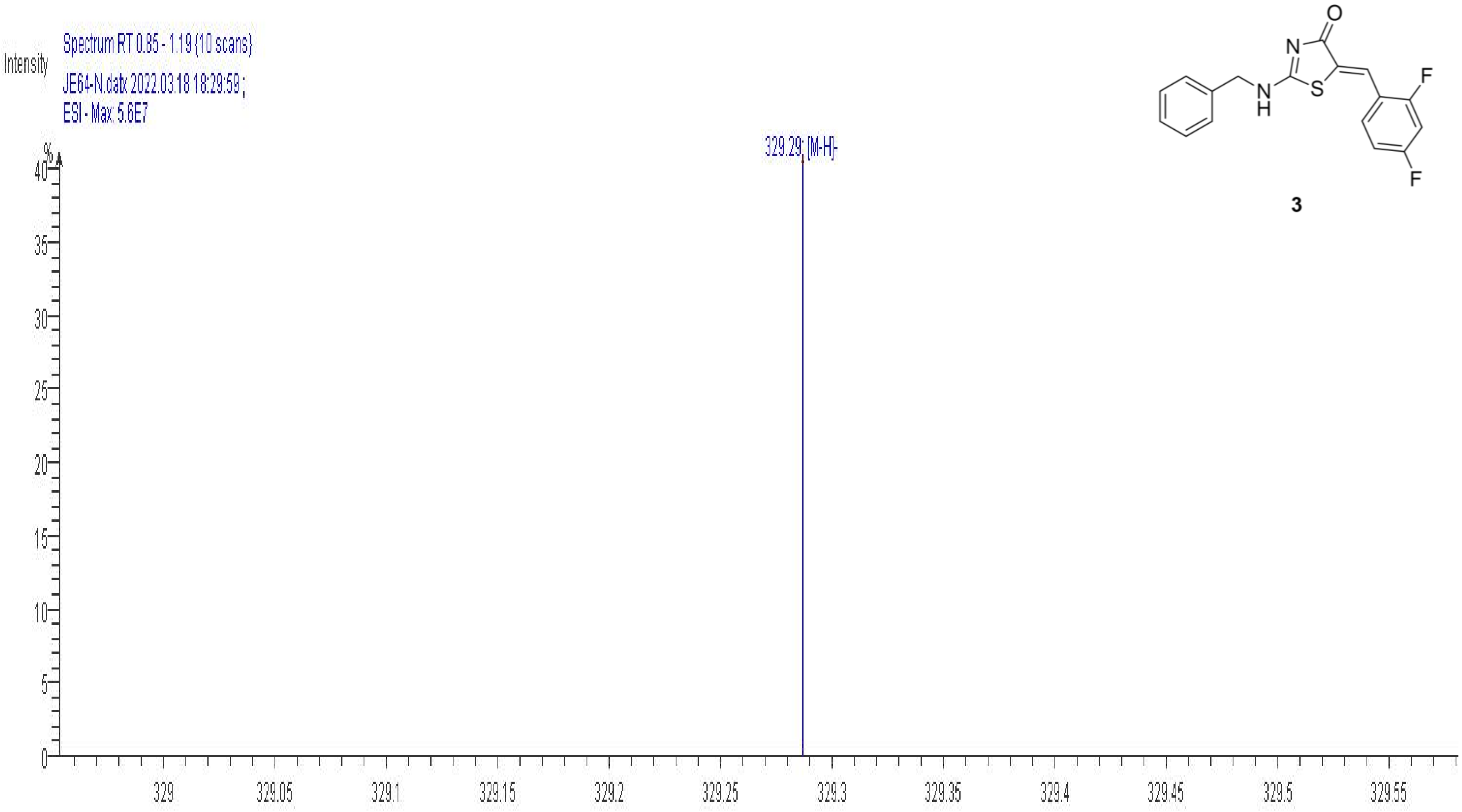

Figure S17. LRMS (ESI–) spectrum of compound **3**

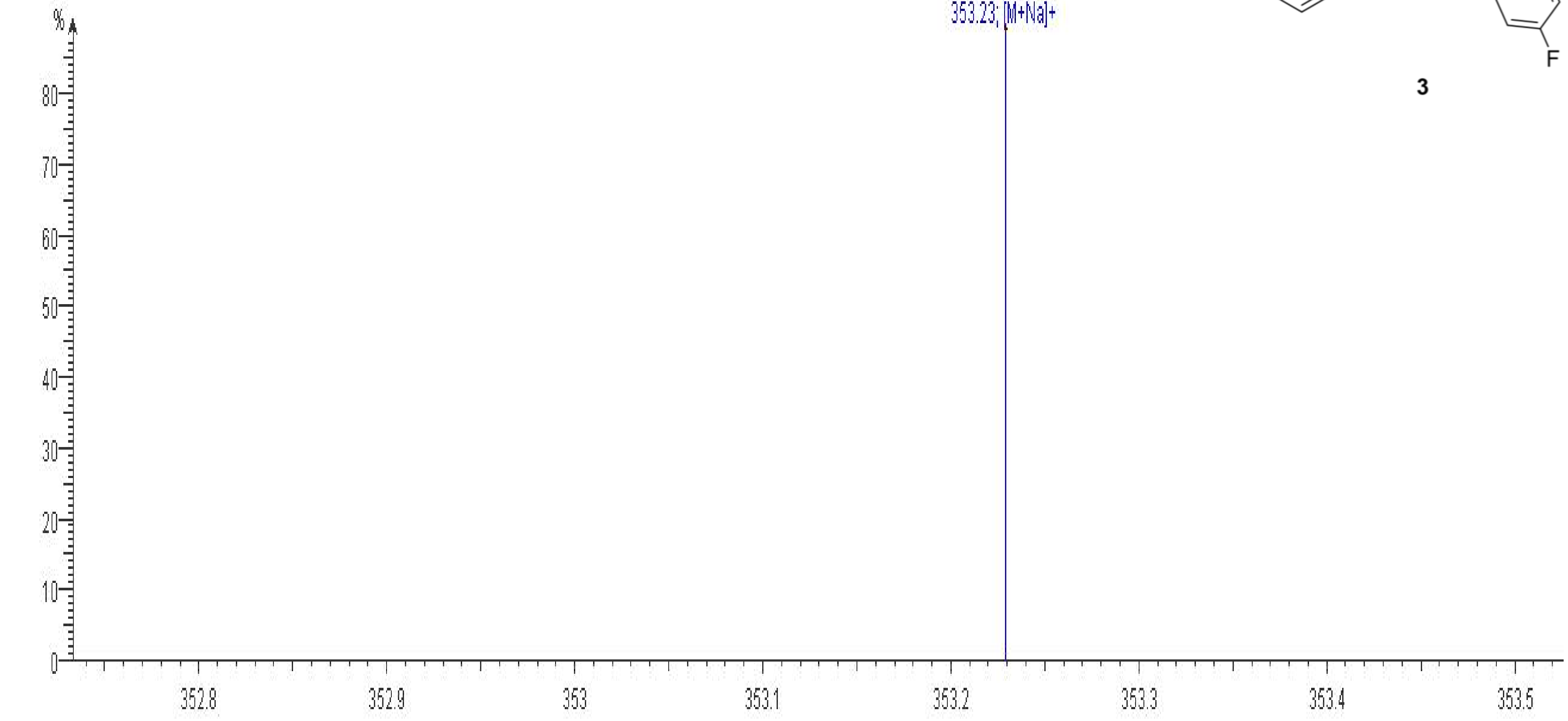

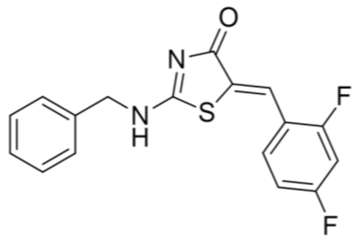

3

Figure S18. LRMS (ESI+) spectrum of compound **3**

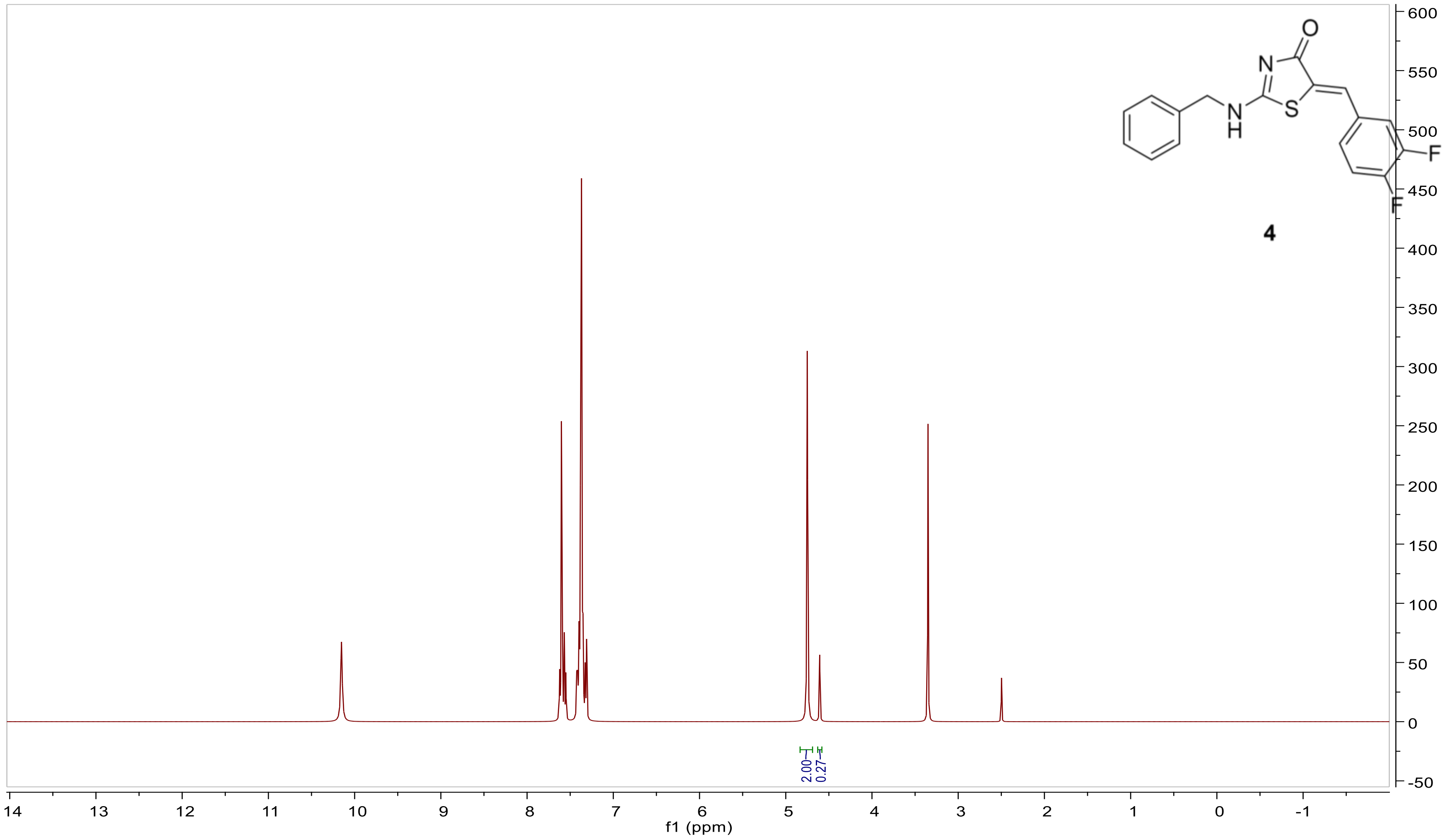

Figure S19. ^{1}H NMR spectrum of compound **4**

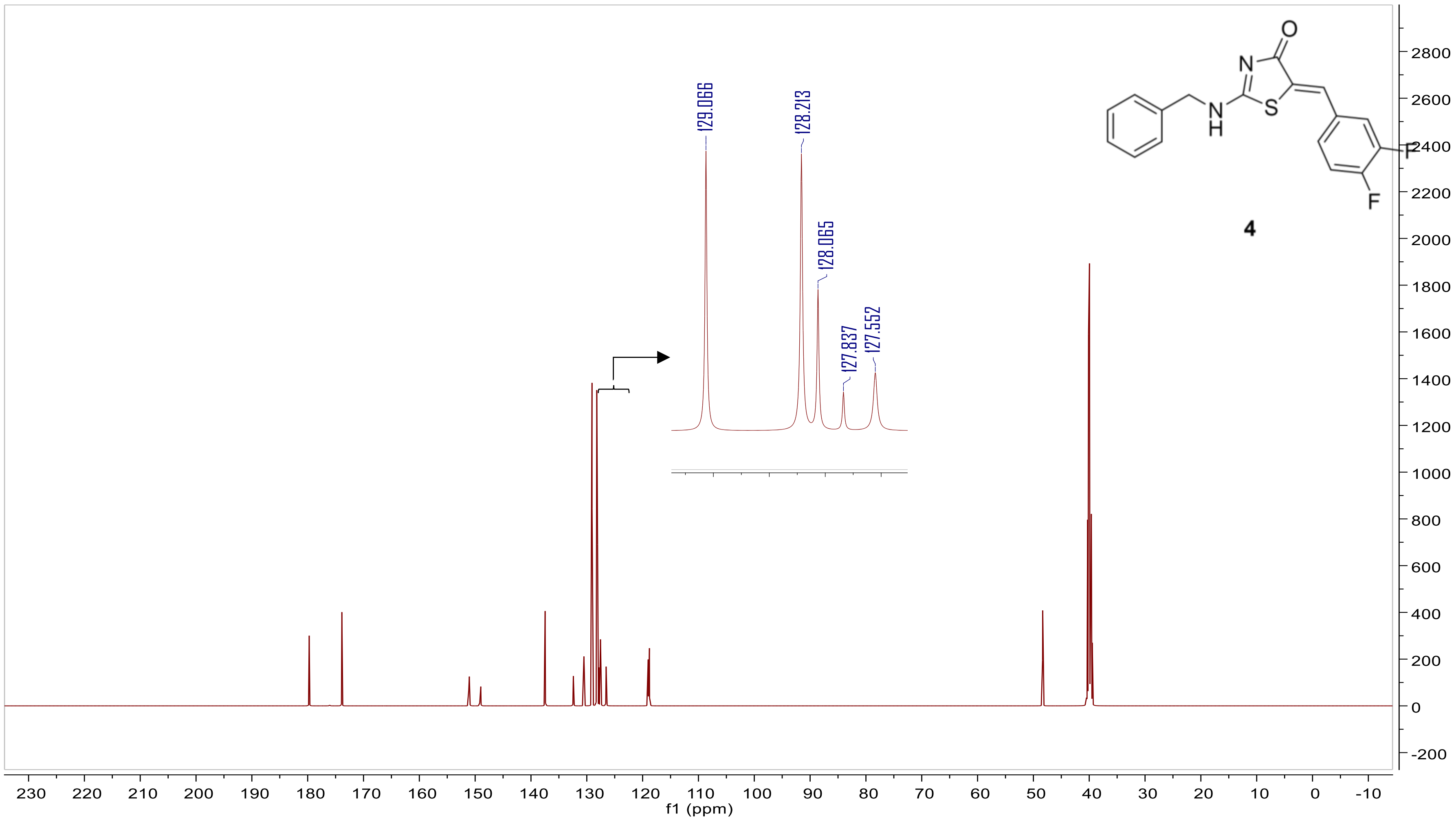

Figure S20. ^{13}C NMR spectrum of compound **4**

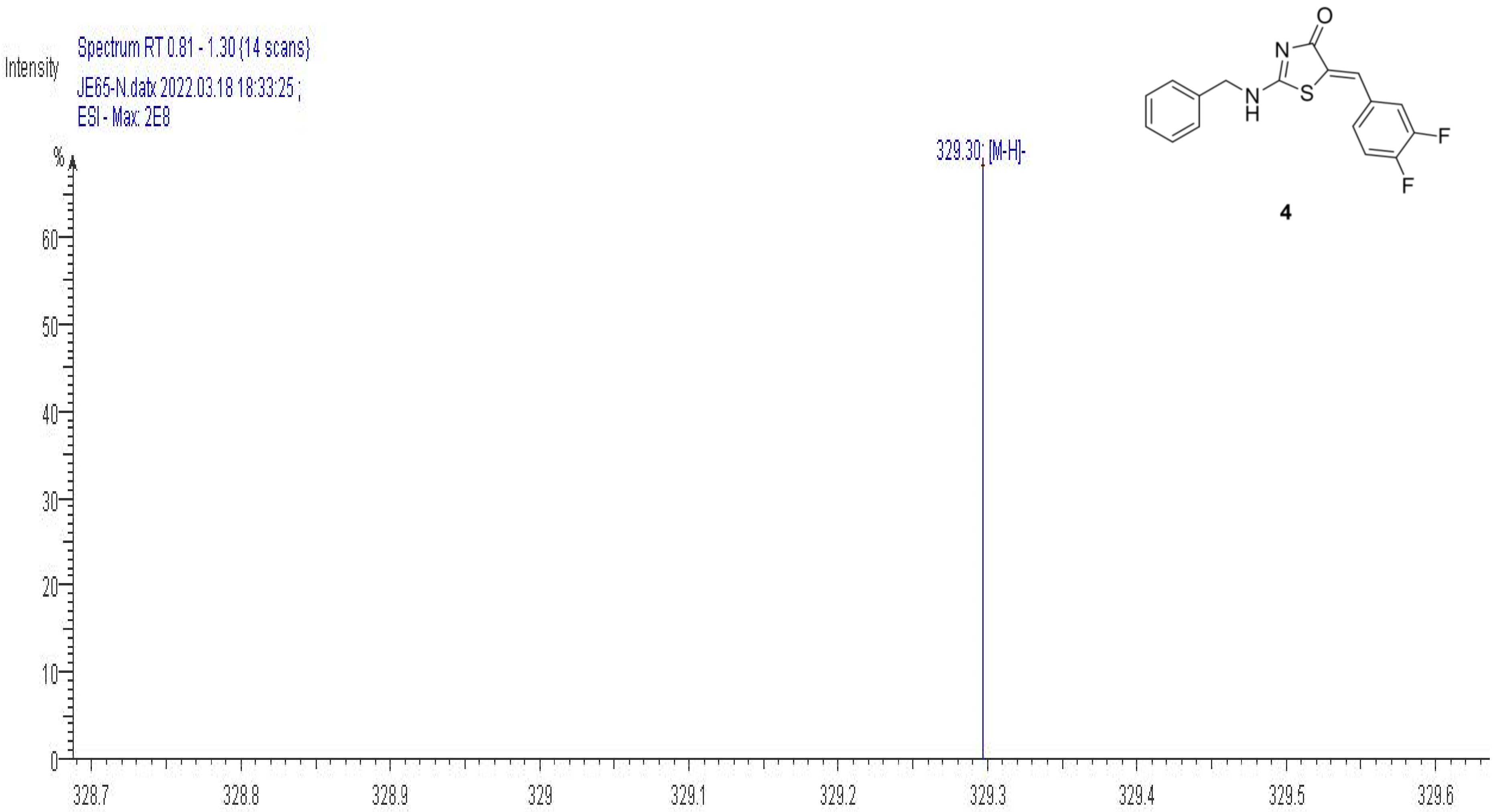

Figure S21. LRMS (ESI–) spectrum of compound **4**

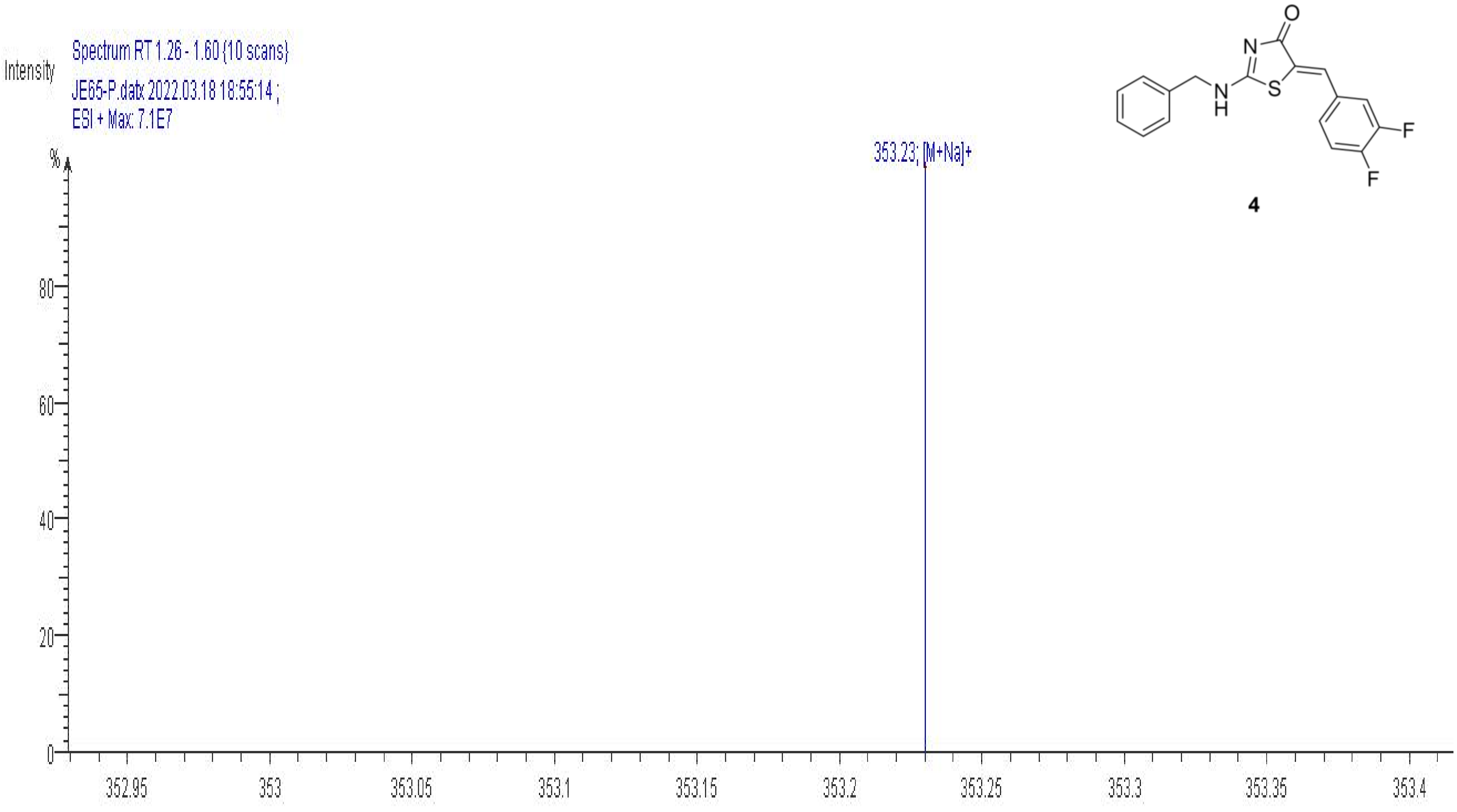

Figure S22. LRMS (ESI+) spectrum of compound **4**

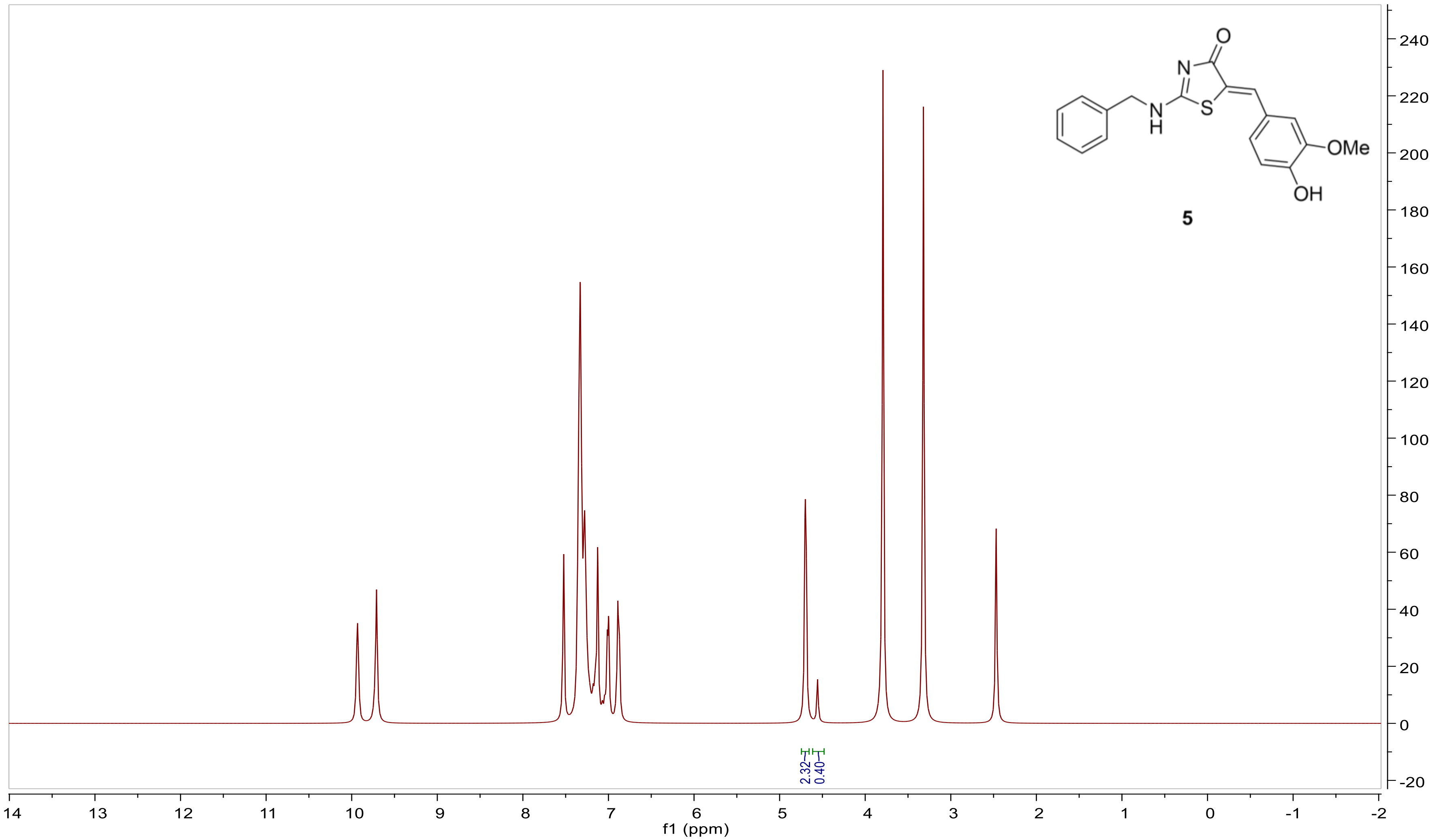

Figure S23. ^{1}H NMR spectrum of compound **5**

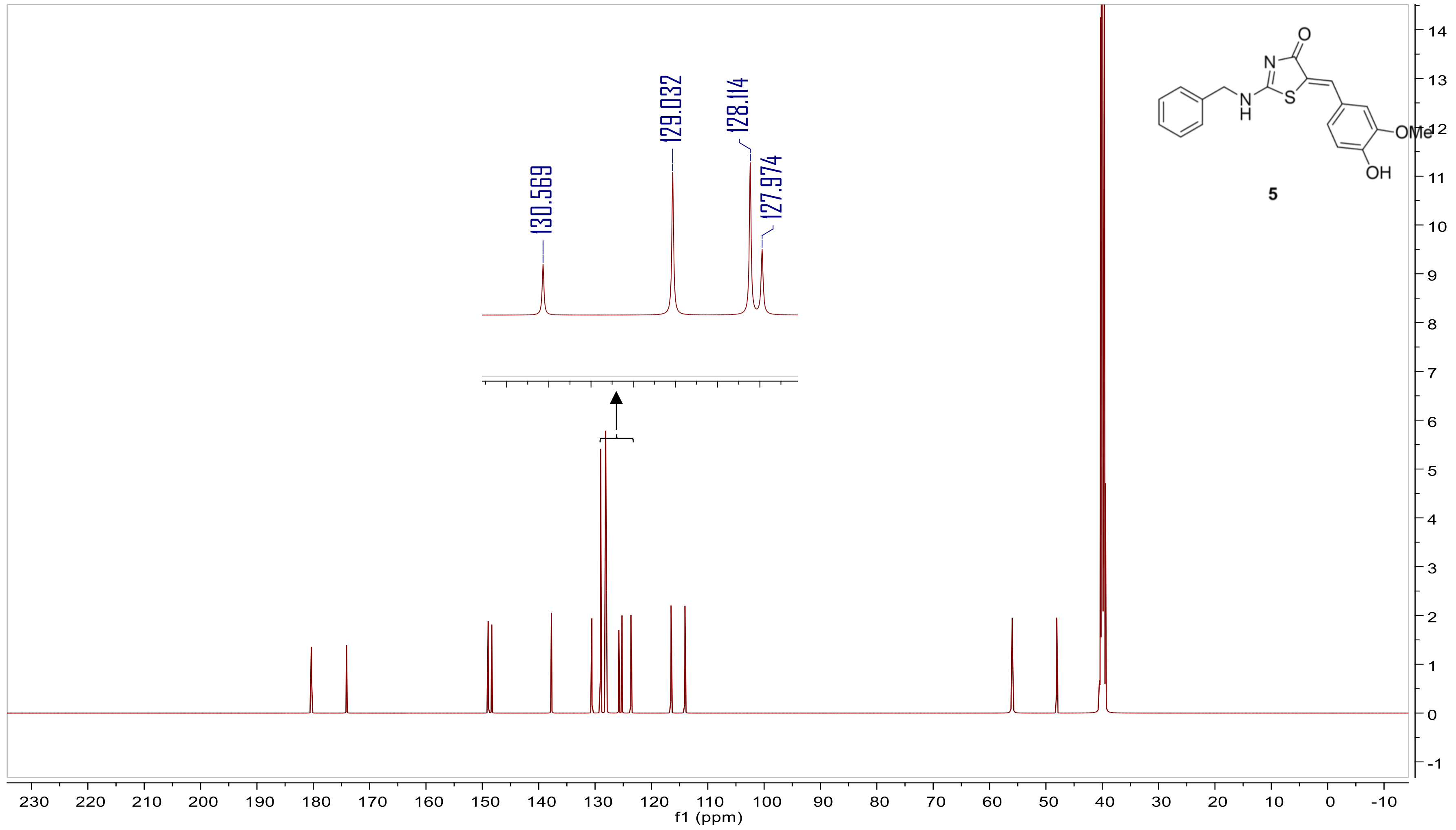

Figure S24. ^{13}C NMR spectrum of compound **5**

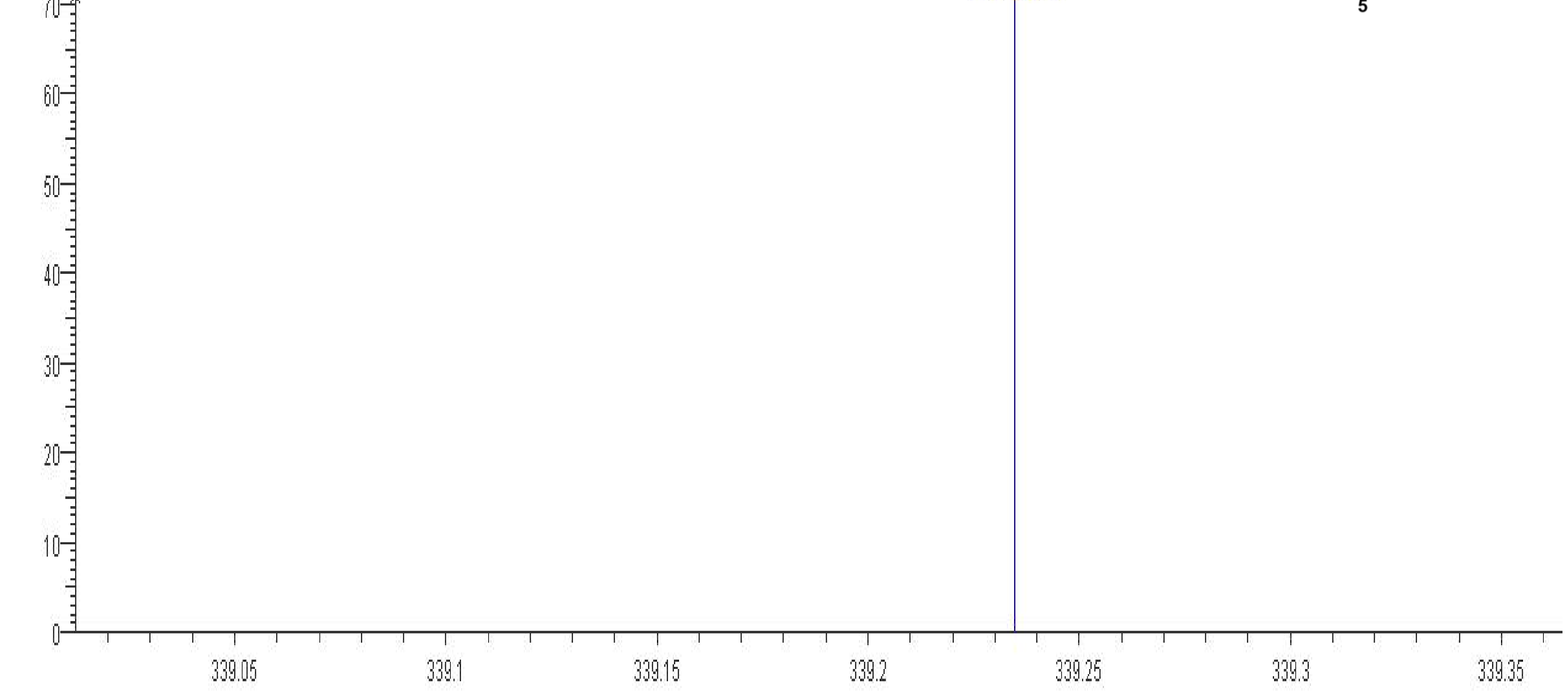

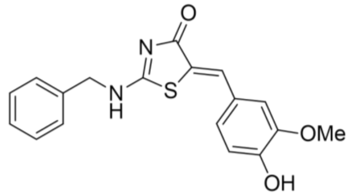

5

Figure S25. LRMS (ESI–) spectrum of compound **5**

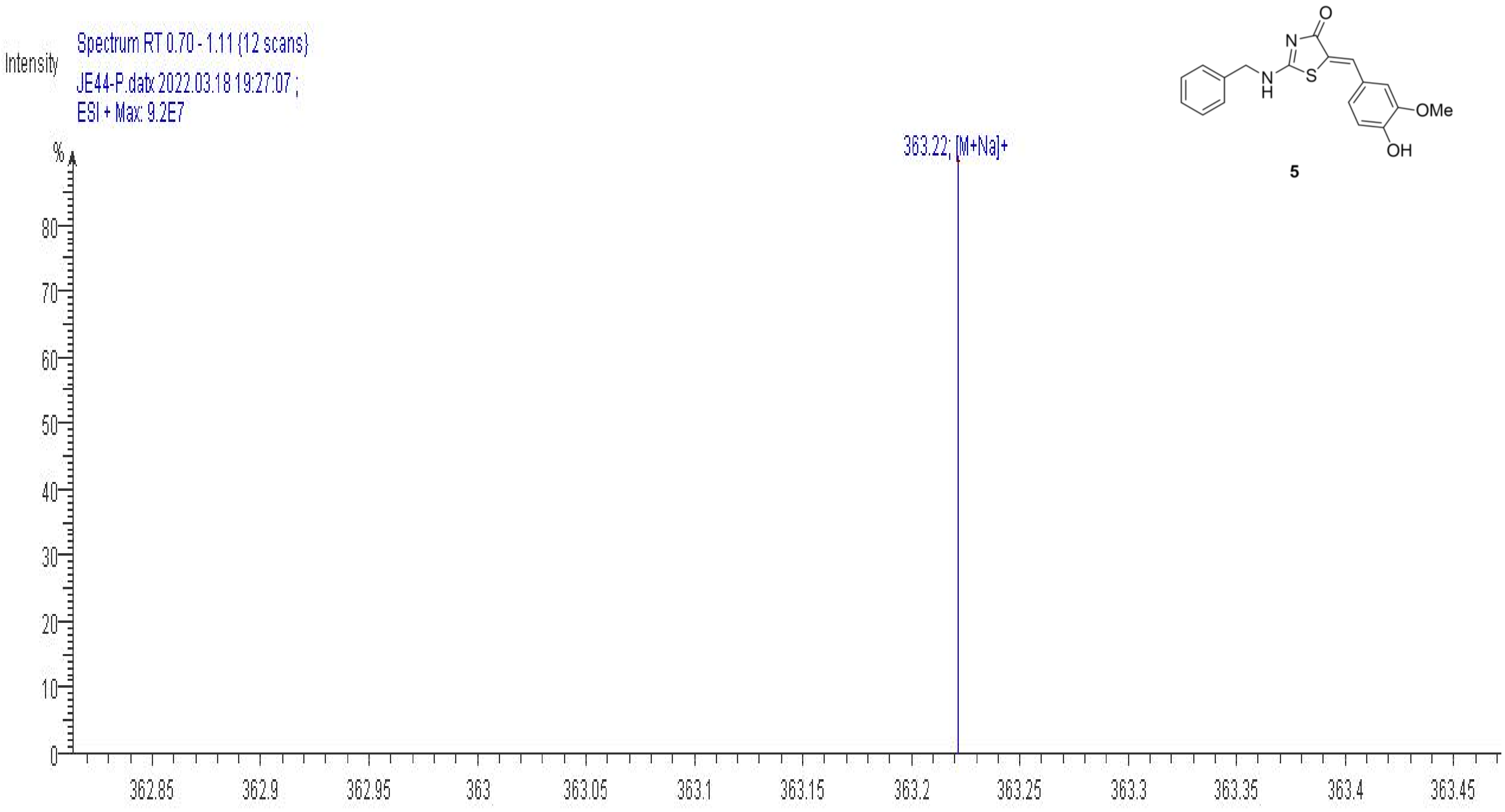

Figure S26. LRMS (ESI+) spectrum of compound **5**

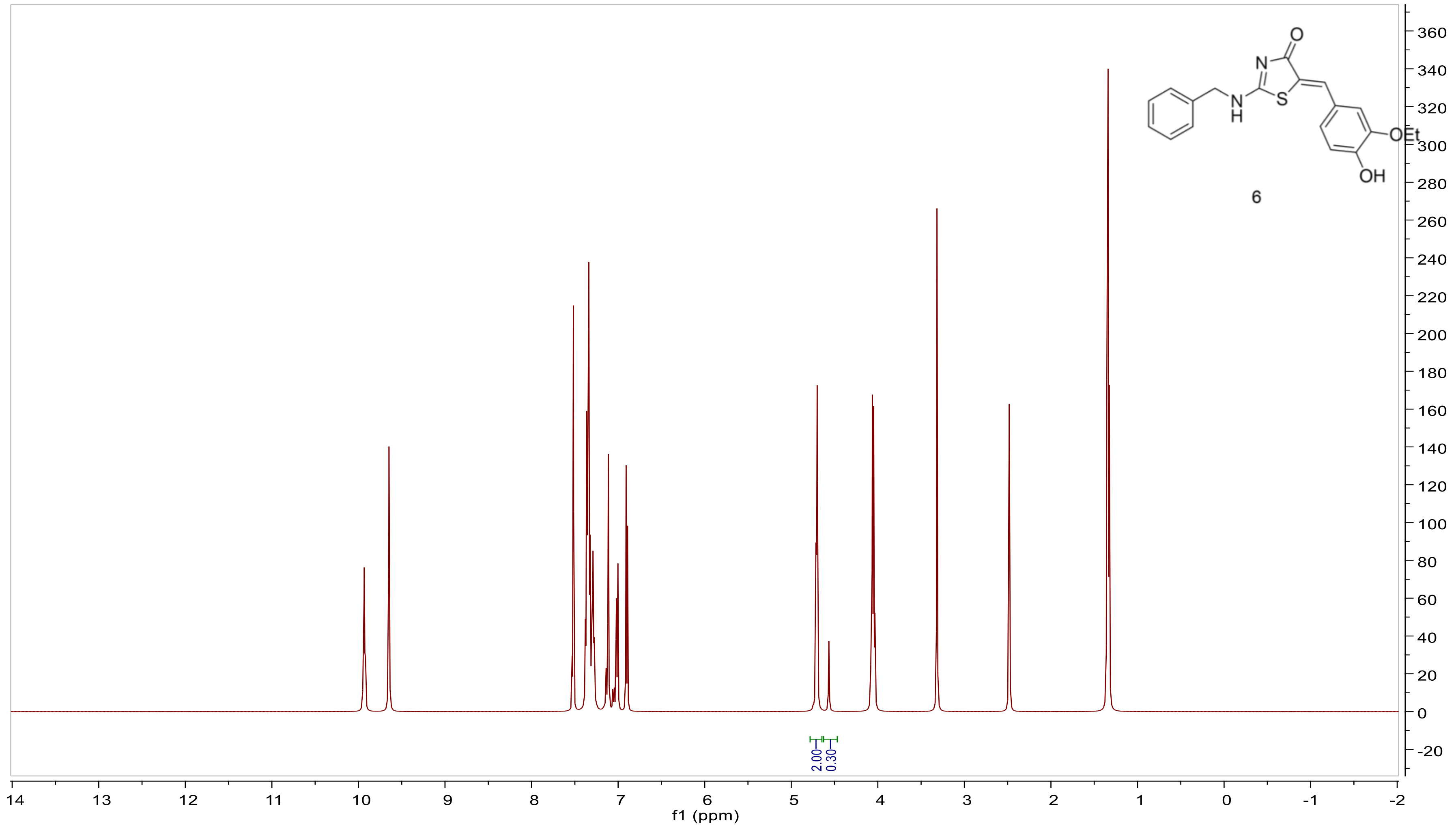

Figure S27. ^{1}H NMR spectrum of compound **6**

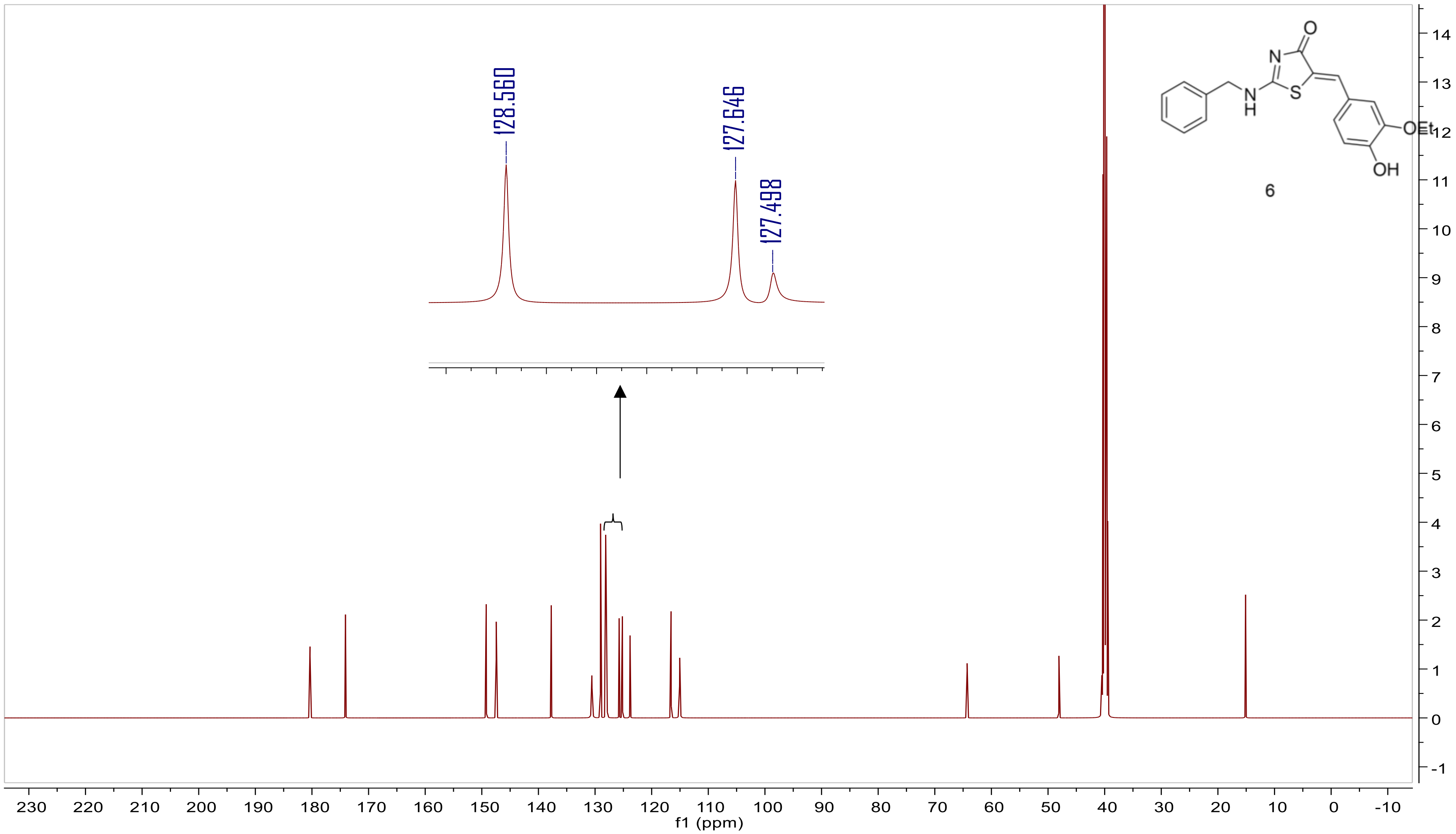

Figure S28. ^{13}C NMR spectrum of compound **6**

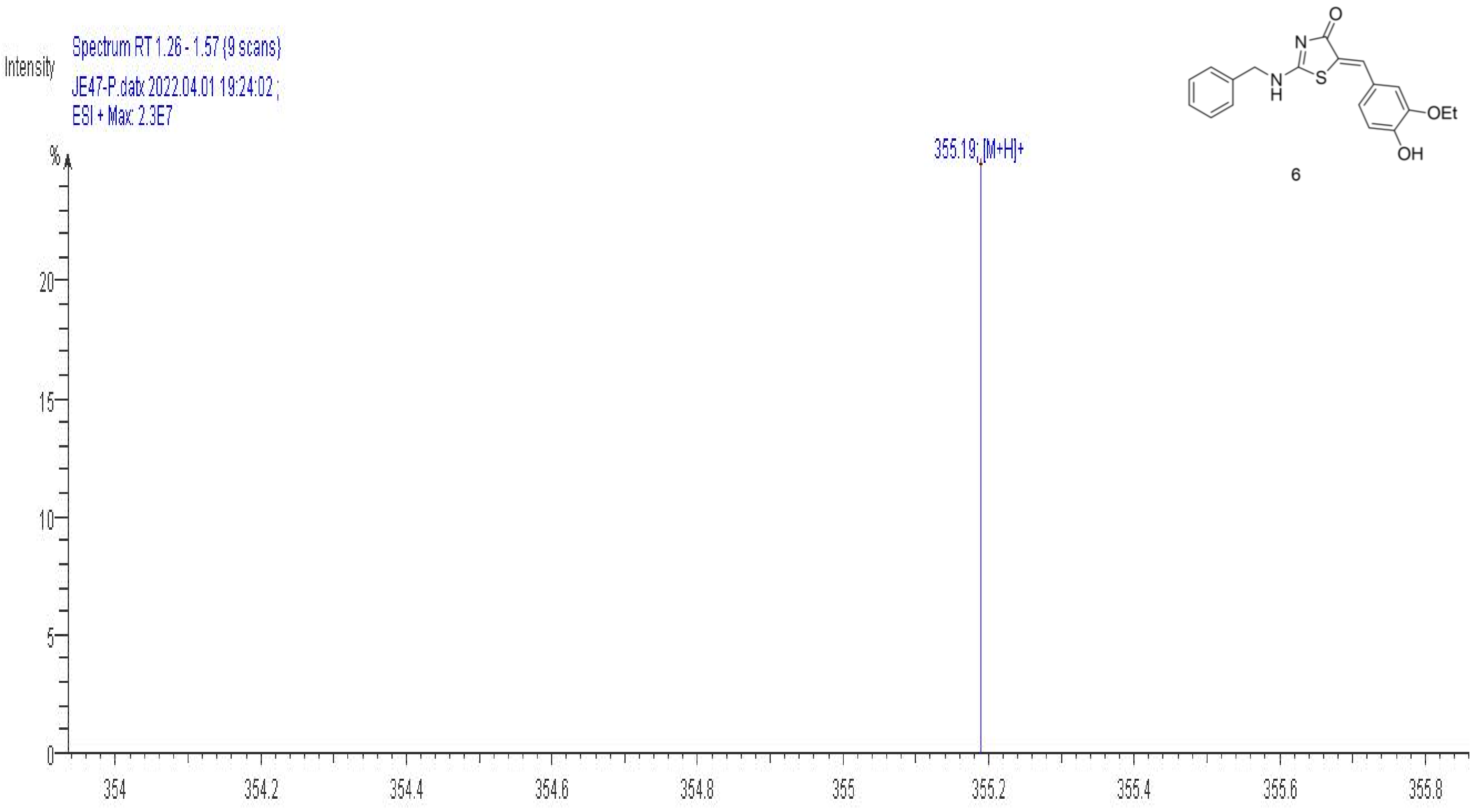

Figure S29. LRMS (ESI+) spectrum of compound **6**

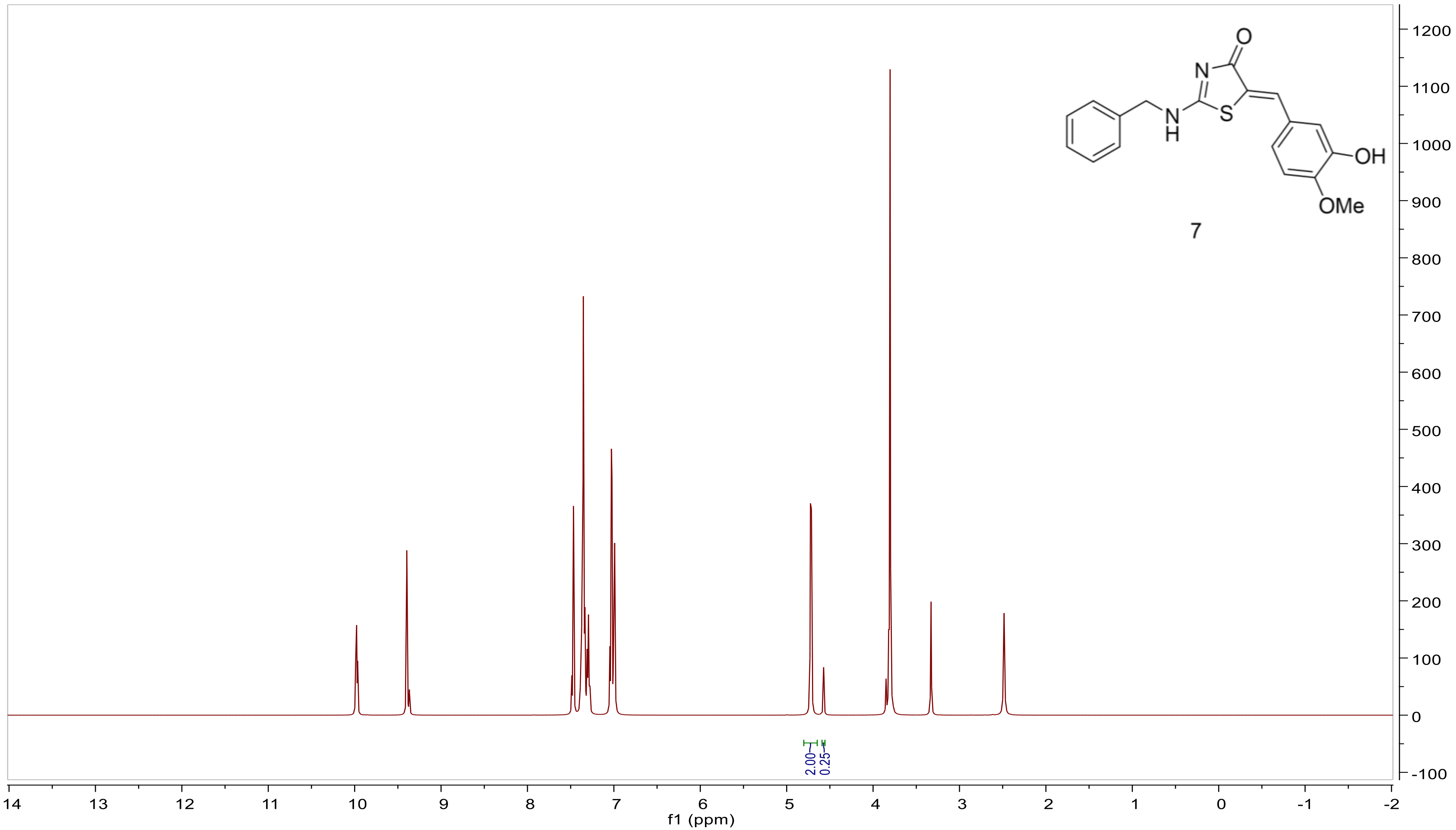

Figure S30. ^{1}H NMR spectrum of compound **7**

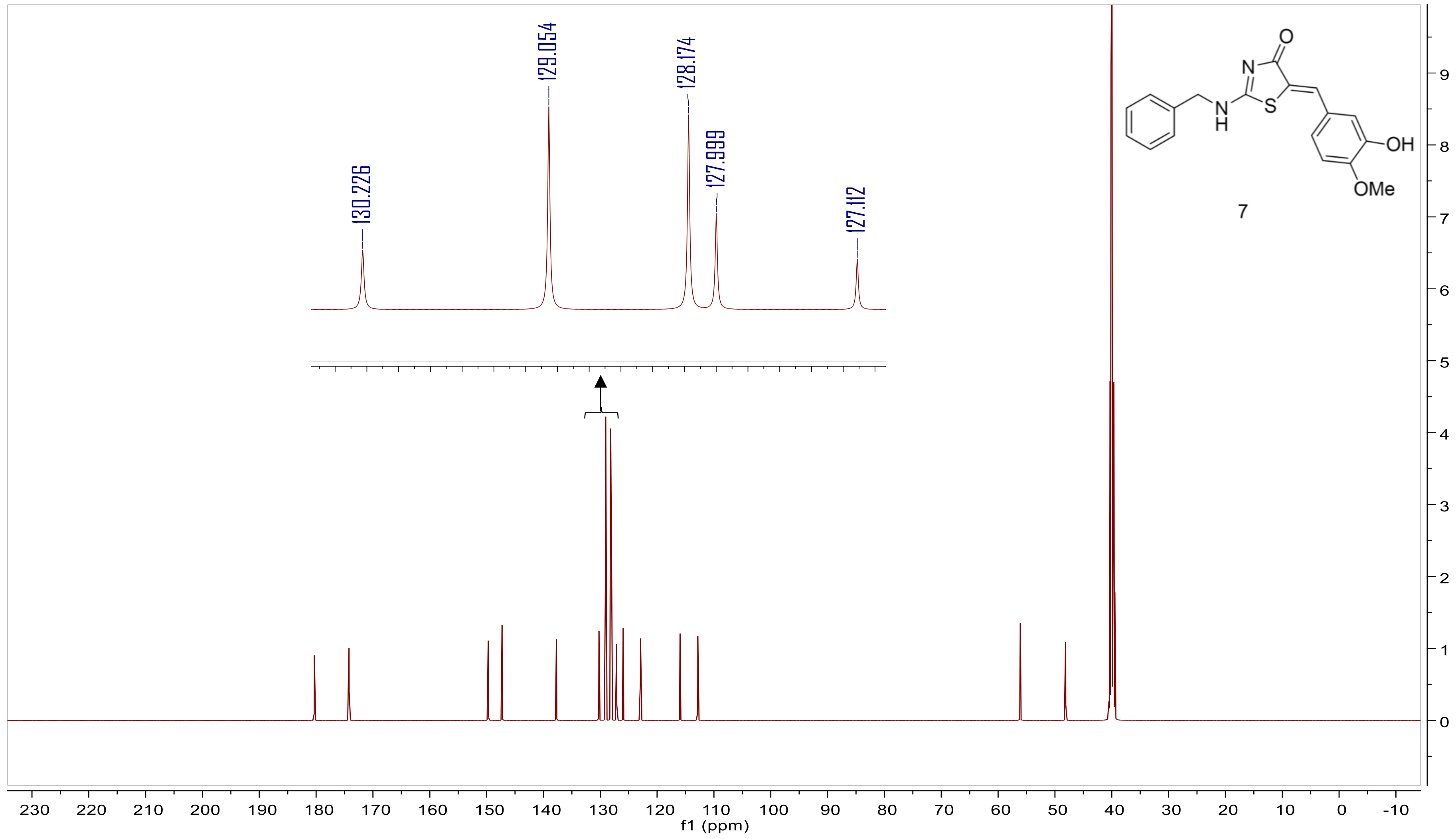

Figure S31. ^{13}C NMR spectrum of compound **7**

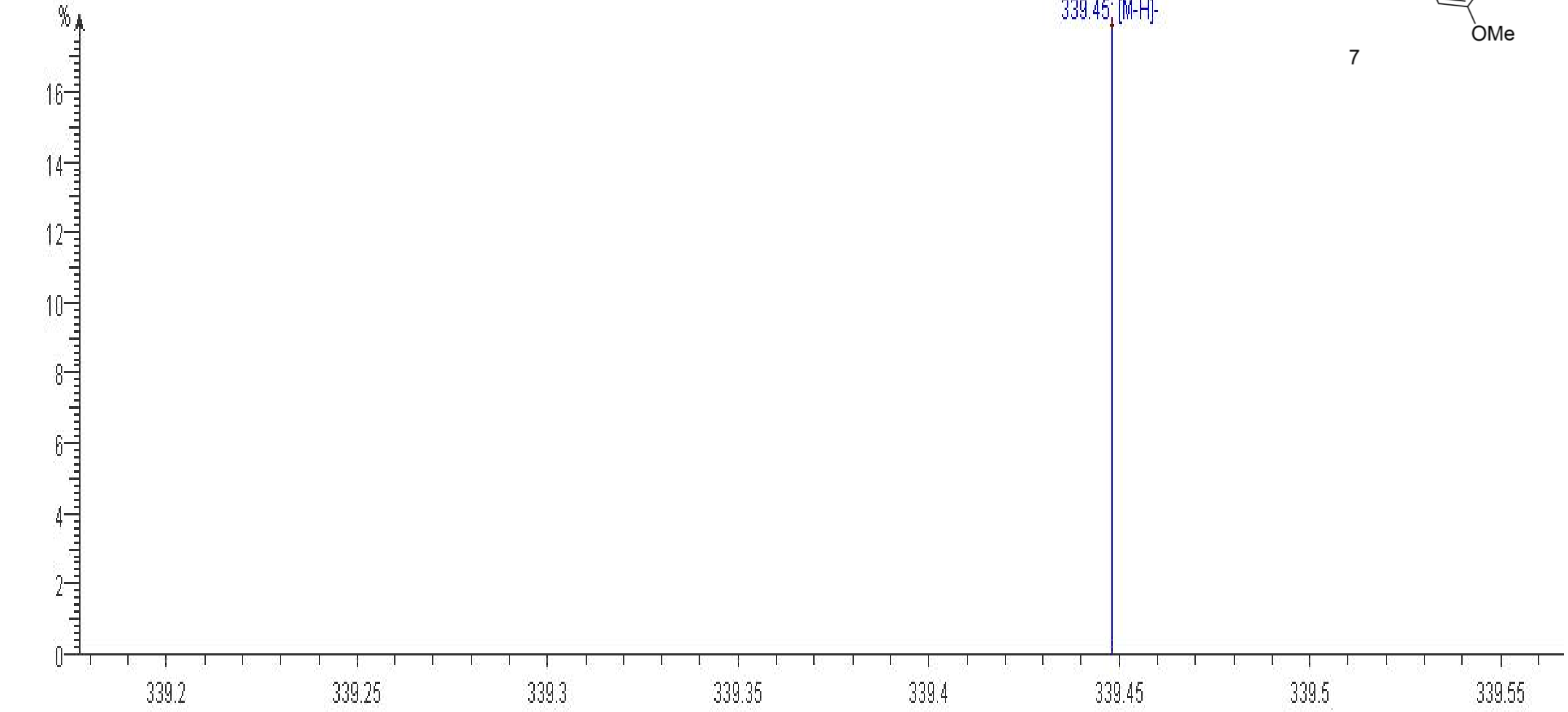

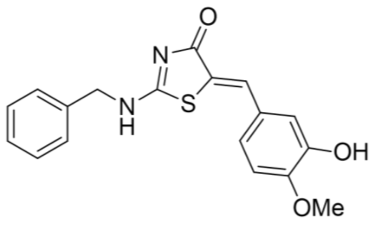

7

Figure S32. LRMS (ESI–) spectrum of compound **7**

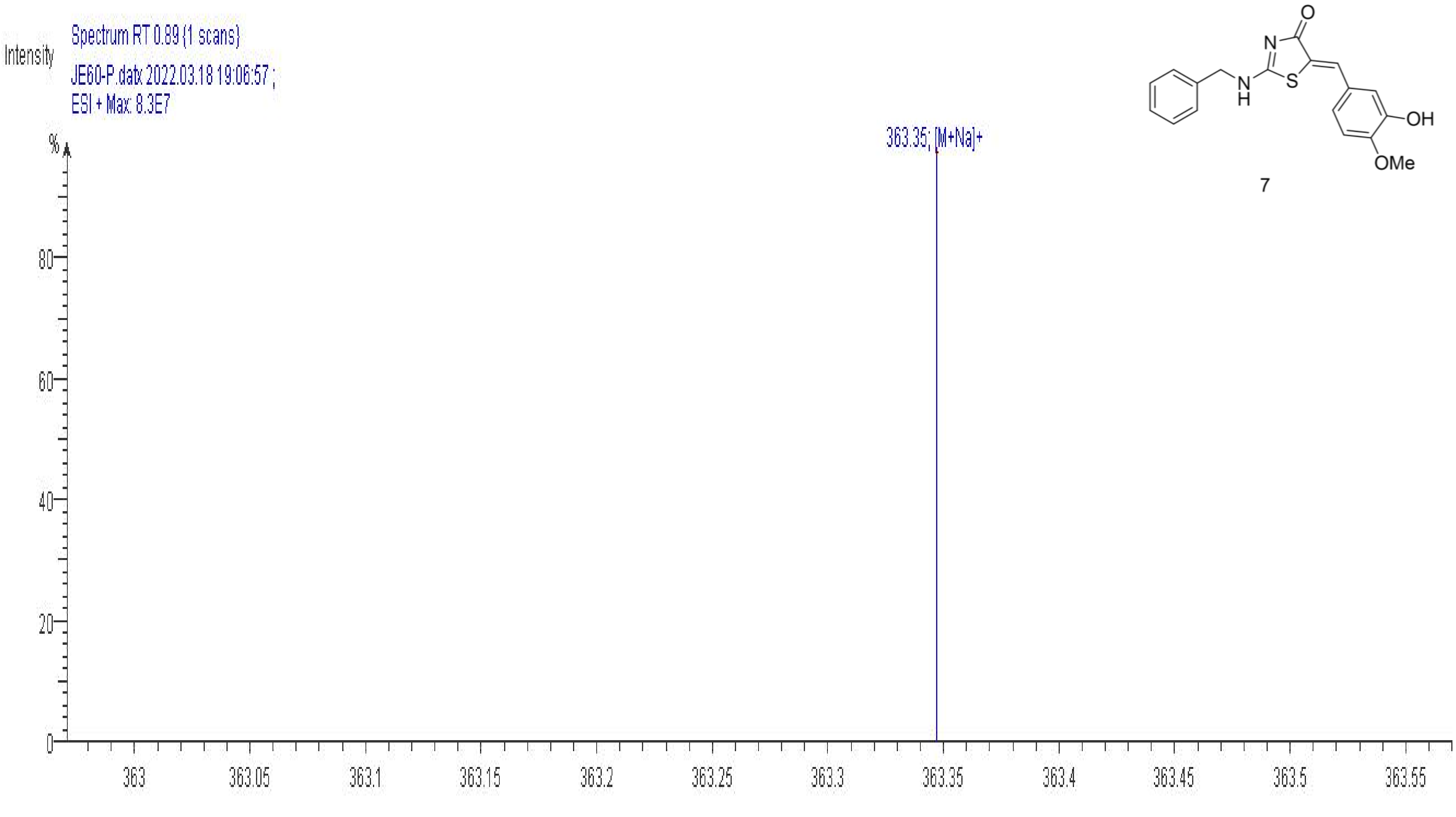

7

Figure S33. LRMS (ESI+) spectrum of compound **7**

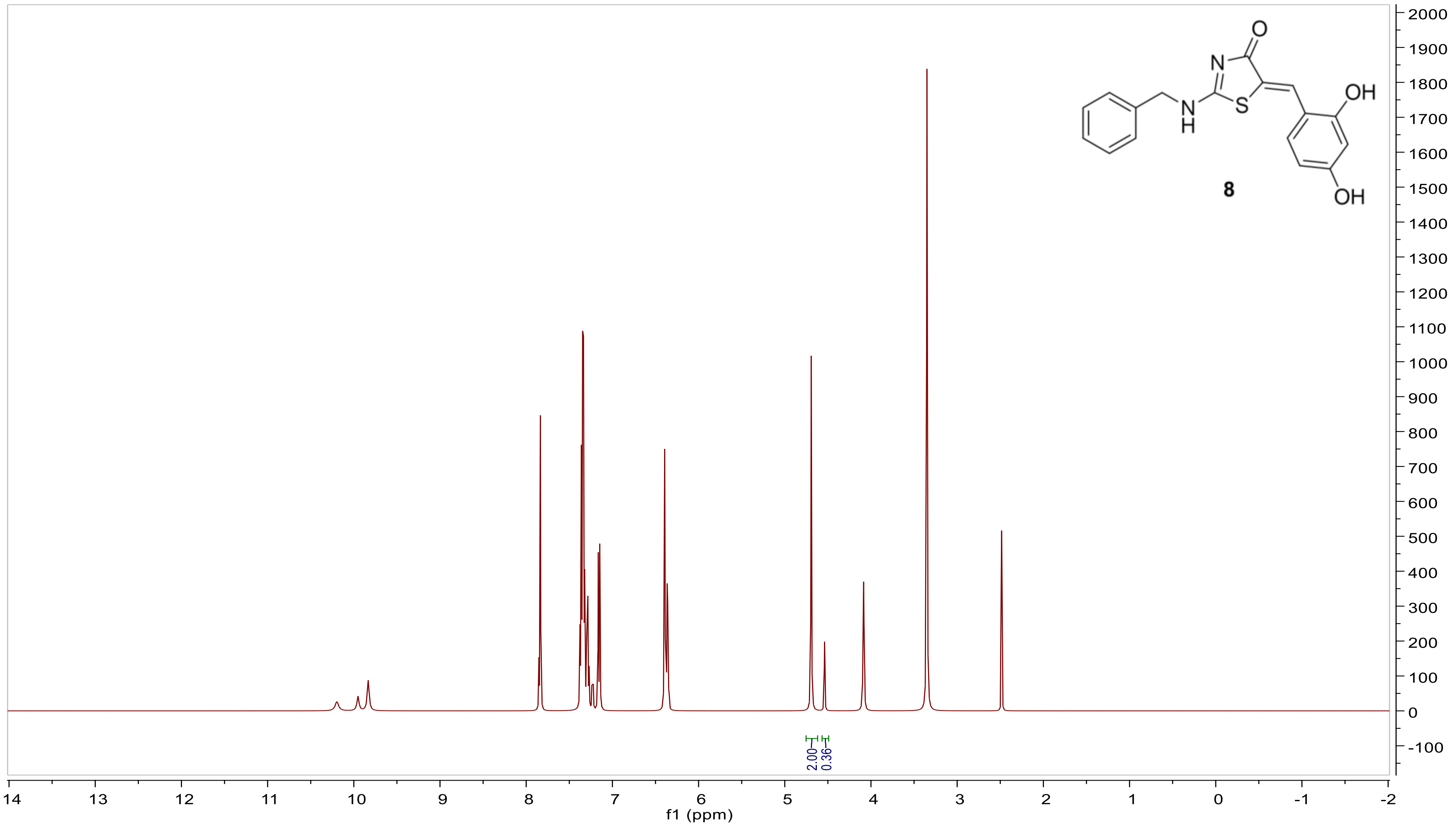

Figure S34. ^{1}H NMR spectrum of compound **8**

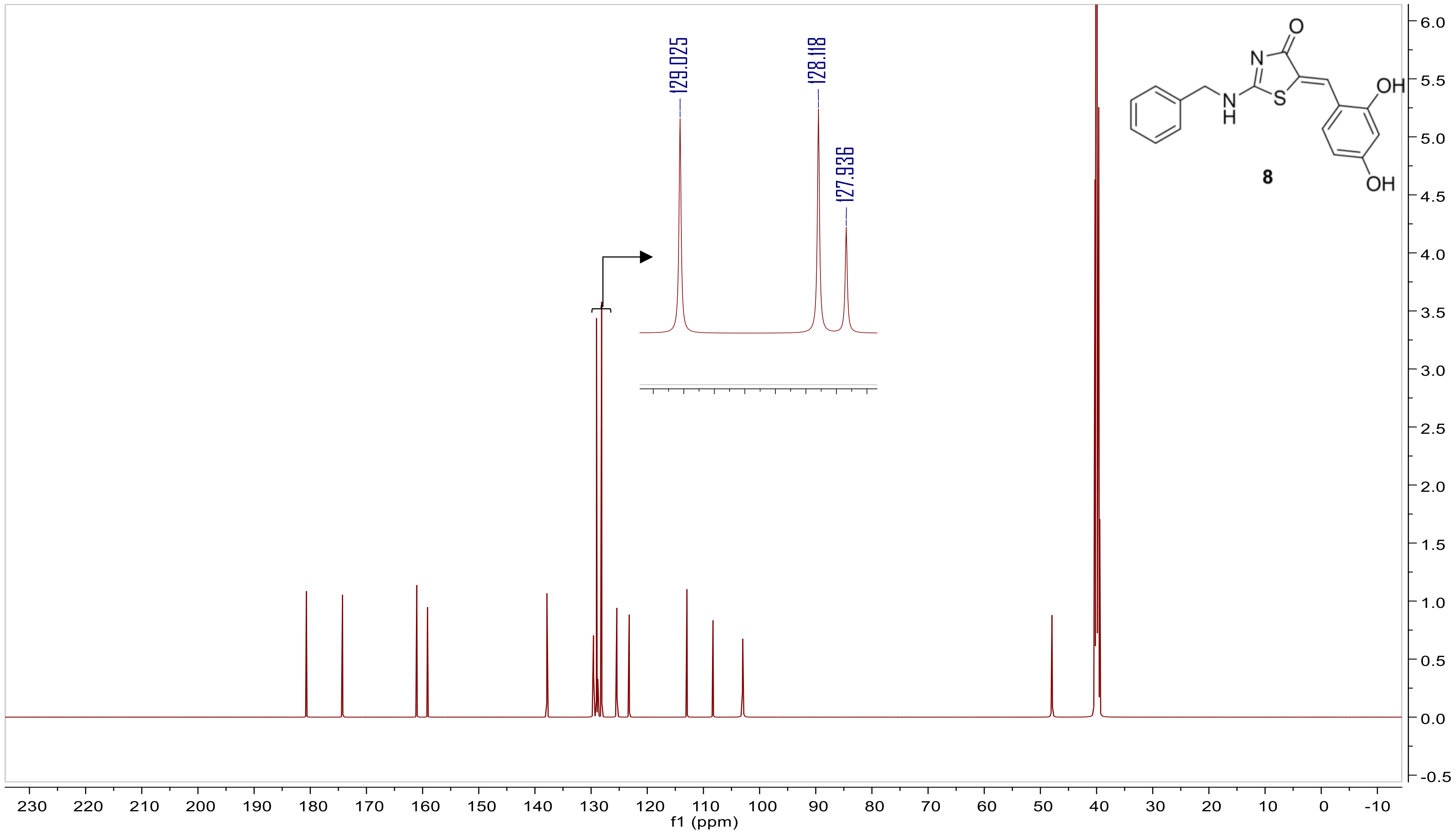

Figure S35. ^{13}C NMR spectrum of compound **8**

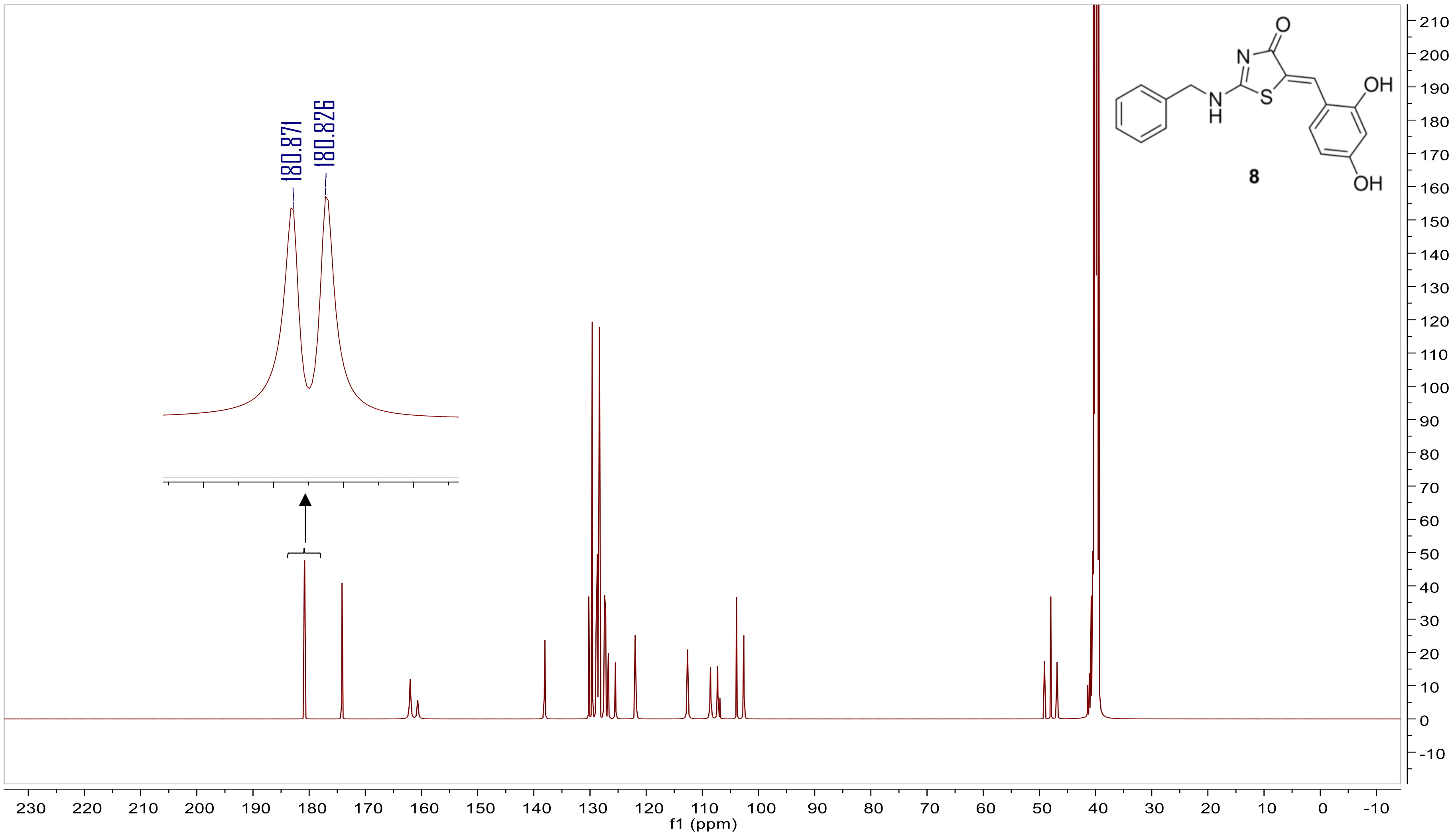

Figure S36. Proton-coupled ^{13}C NMR spectrum of compound **8**

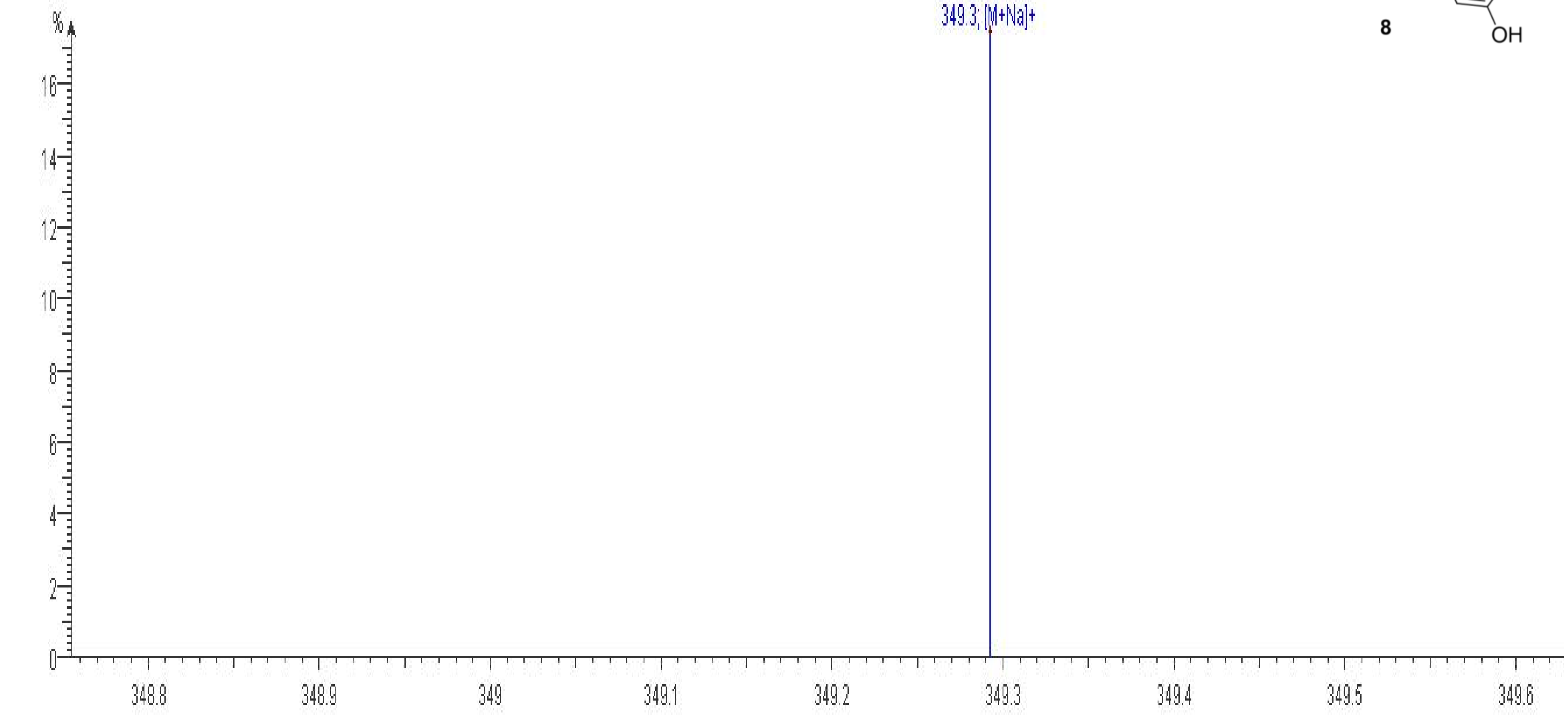

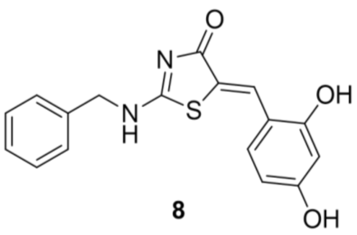

8

Figure S37. LRMS (ESI+) spectrum of compound **8**

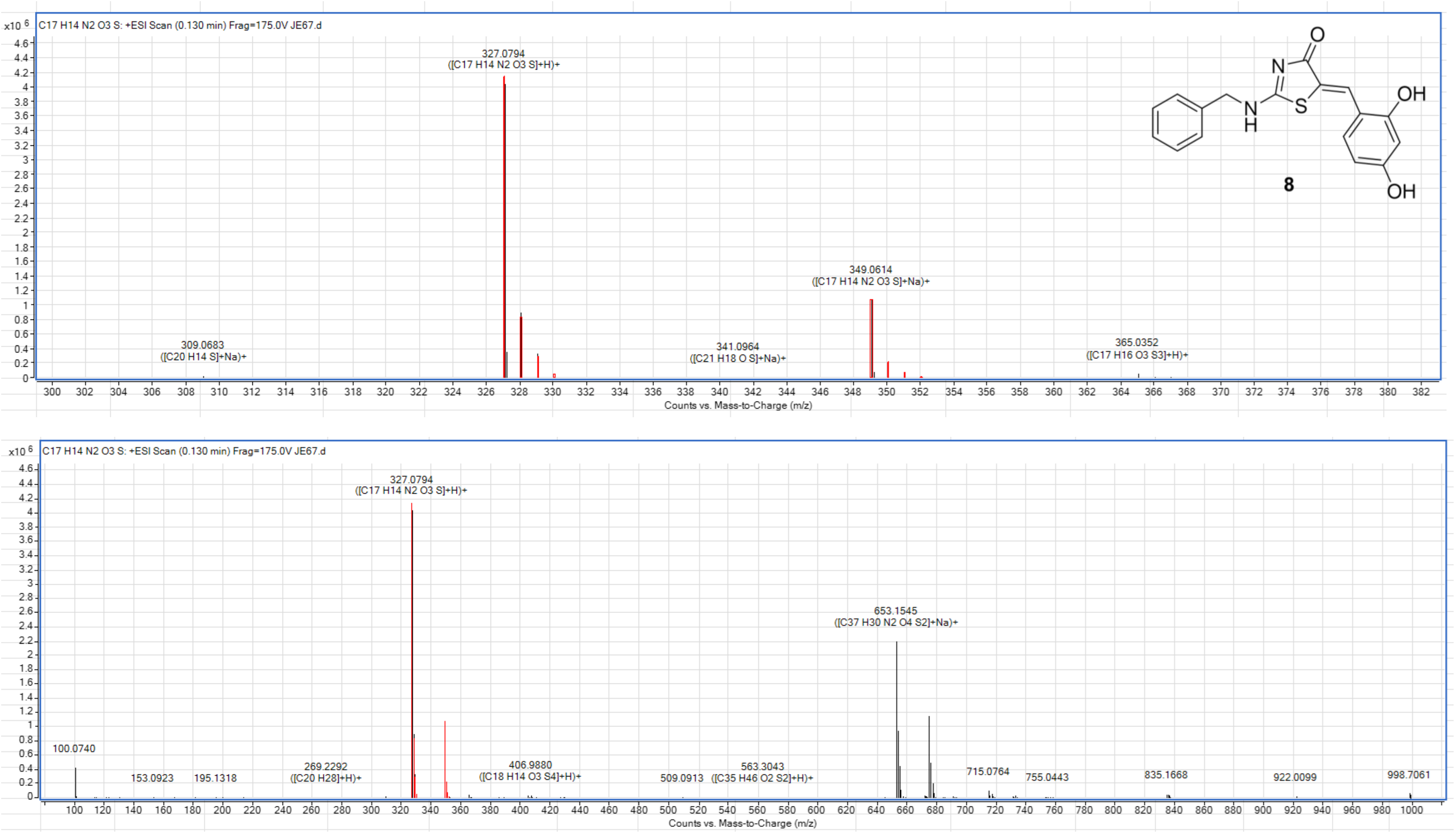

Figure S38. HRMS (ESI+) spectrum of compound **8**

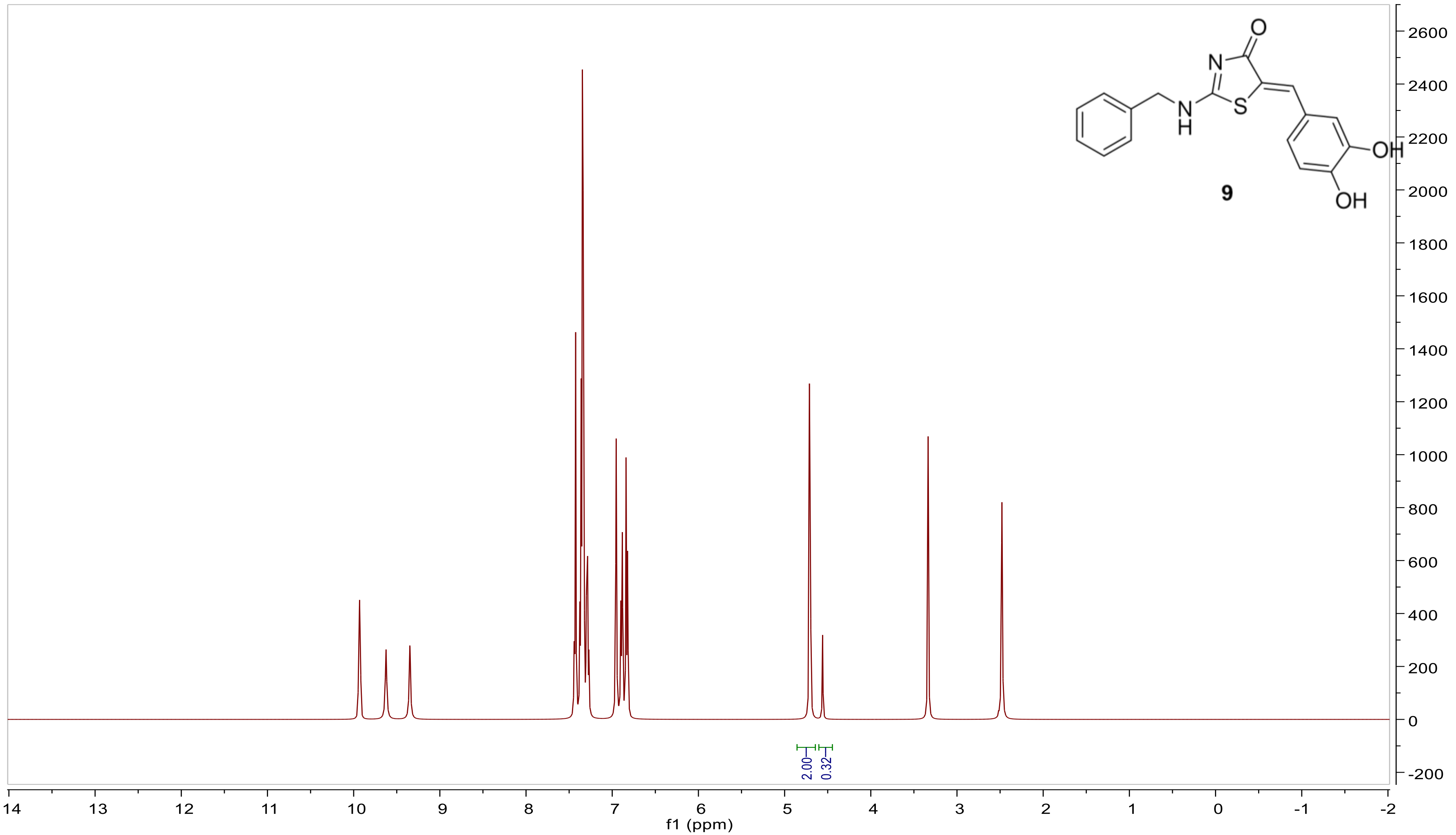

Figure S39. ^{1}H NMR spectrum of compound **9**

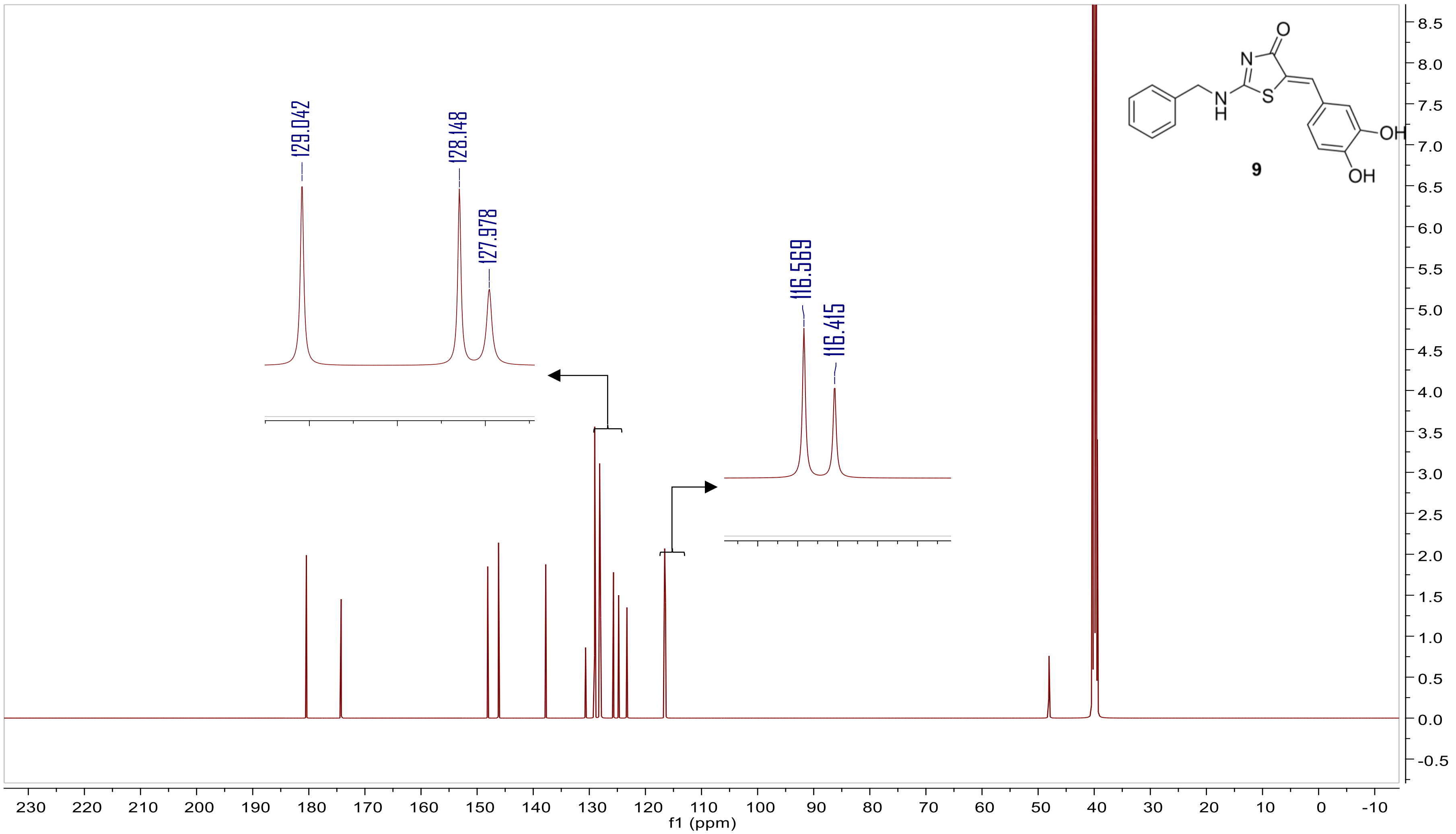

Figure S40. ^{13}C NMR spectrum of compound **9**

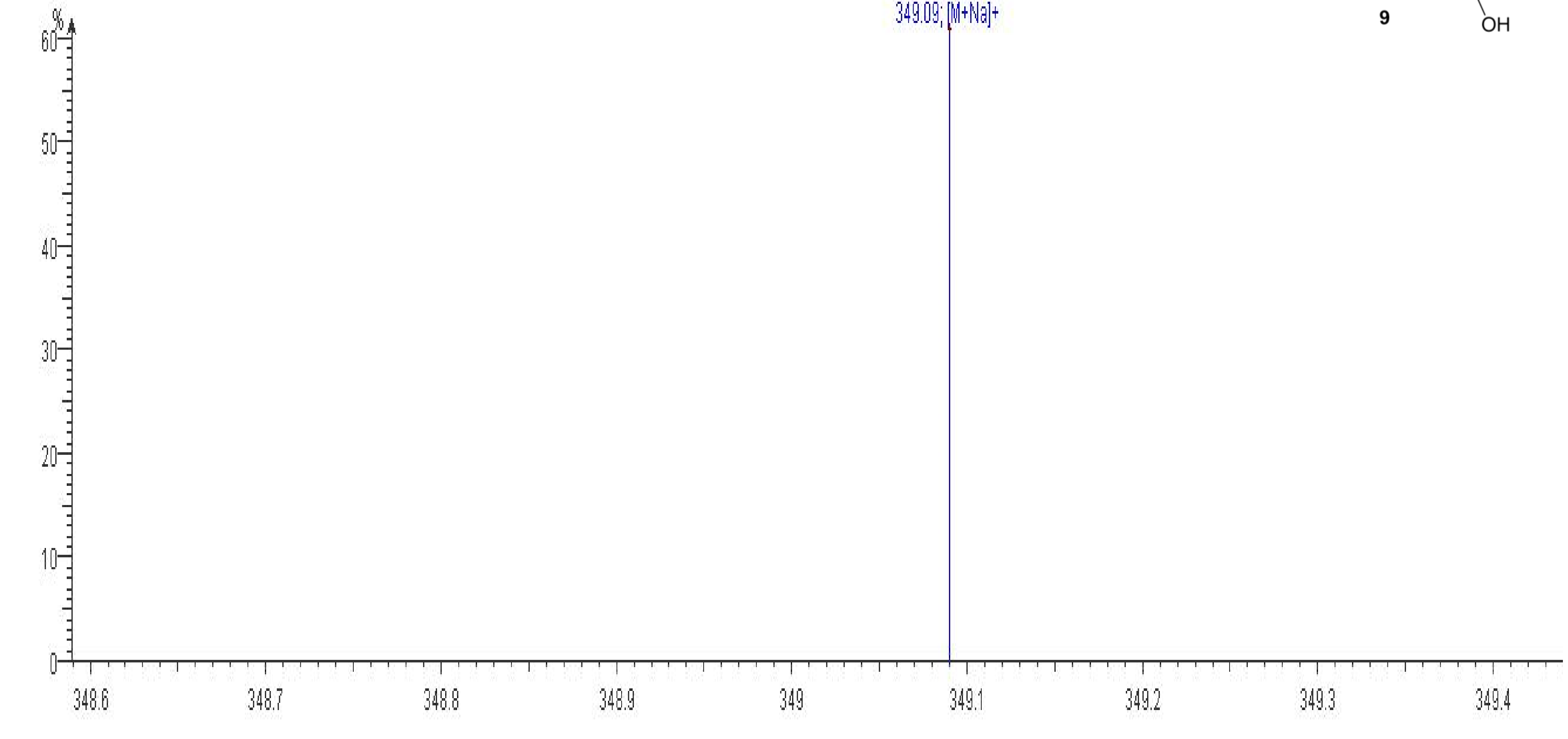

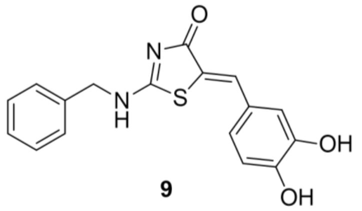

9

Figure S41. LRMS (ESI+) spectrum of compound **9**

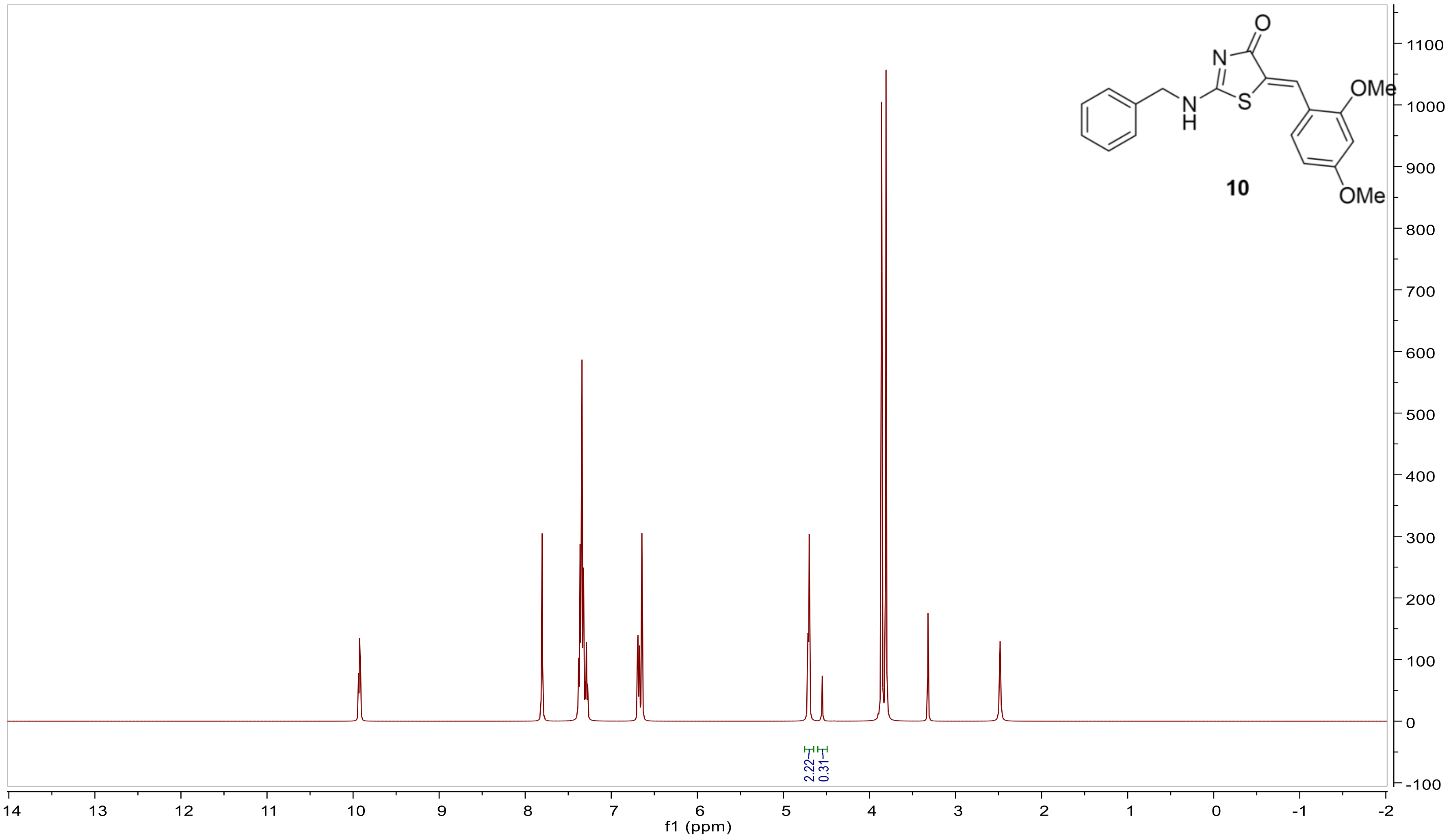

Figure S42. ^{1}H NMR spectrum of compound **10**

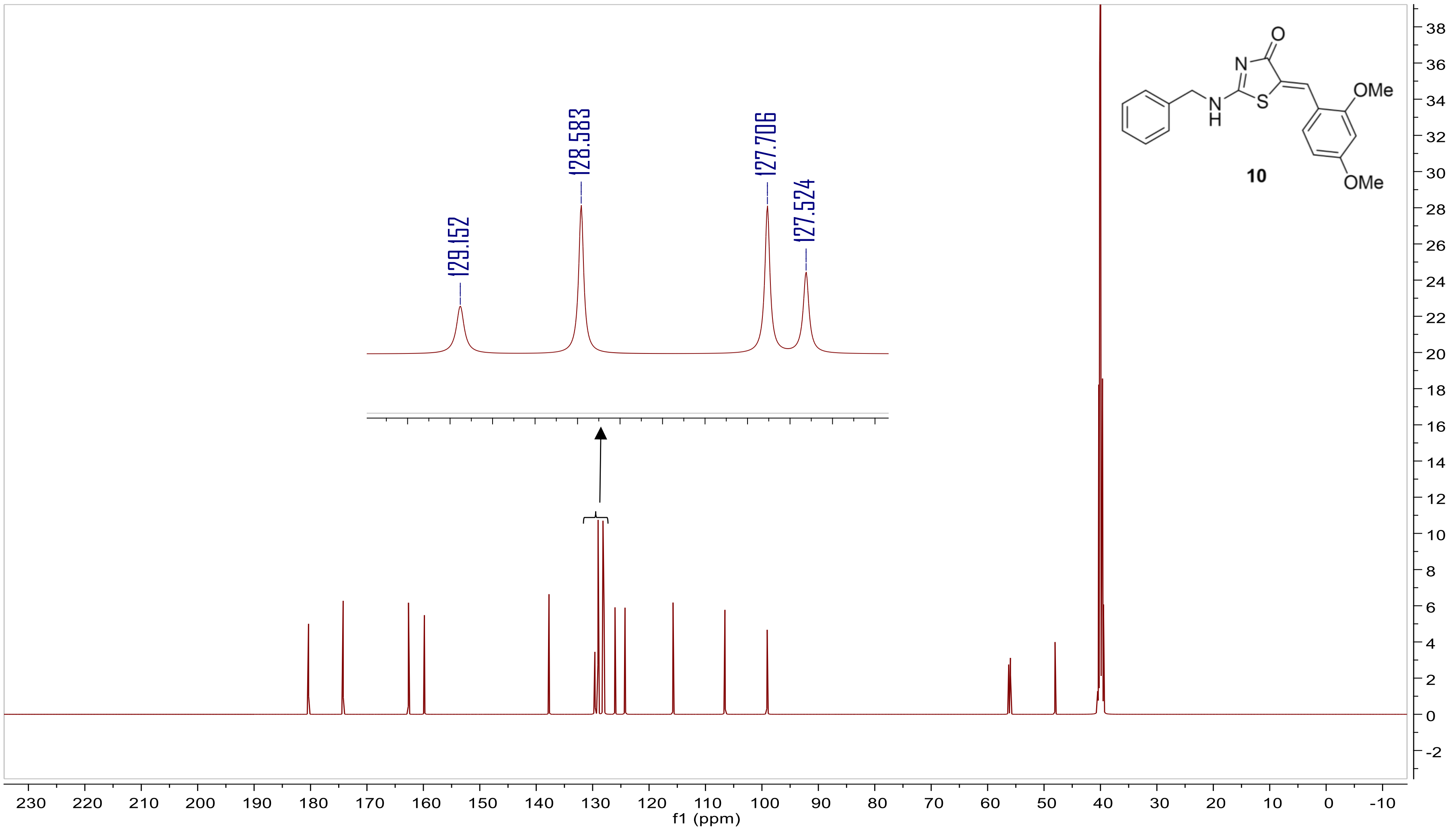

Figure S43. ^{13}C NMR spectrum of compound **10**

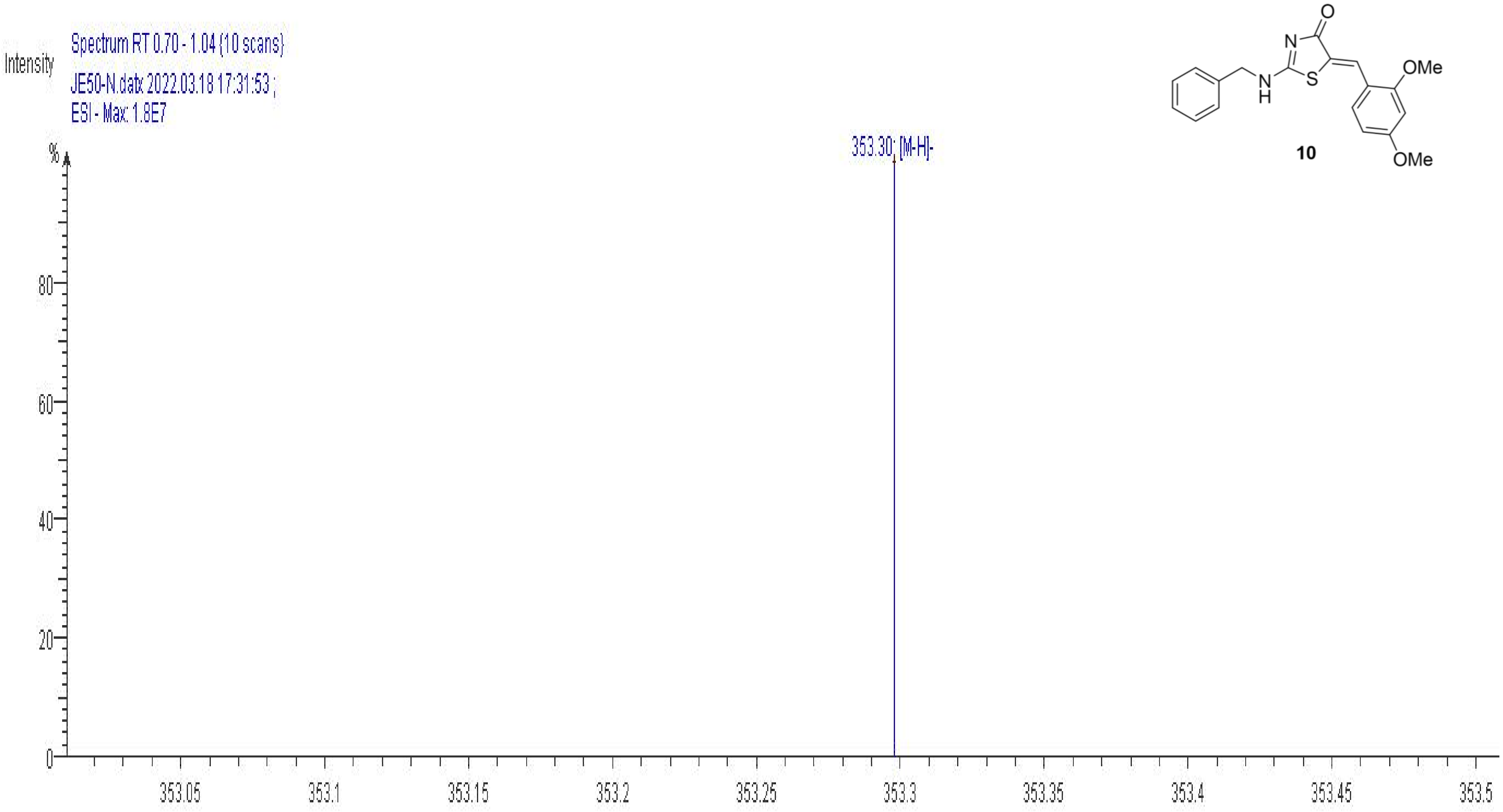

Figure S44. LRMS (ESI–) spectrum of compound **10**

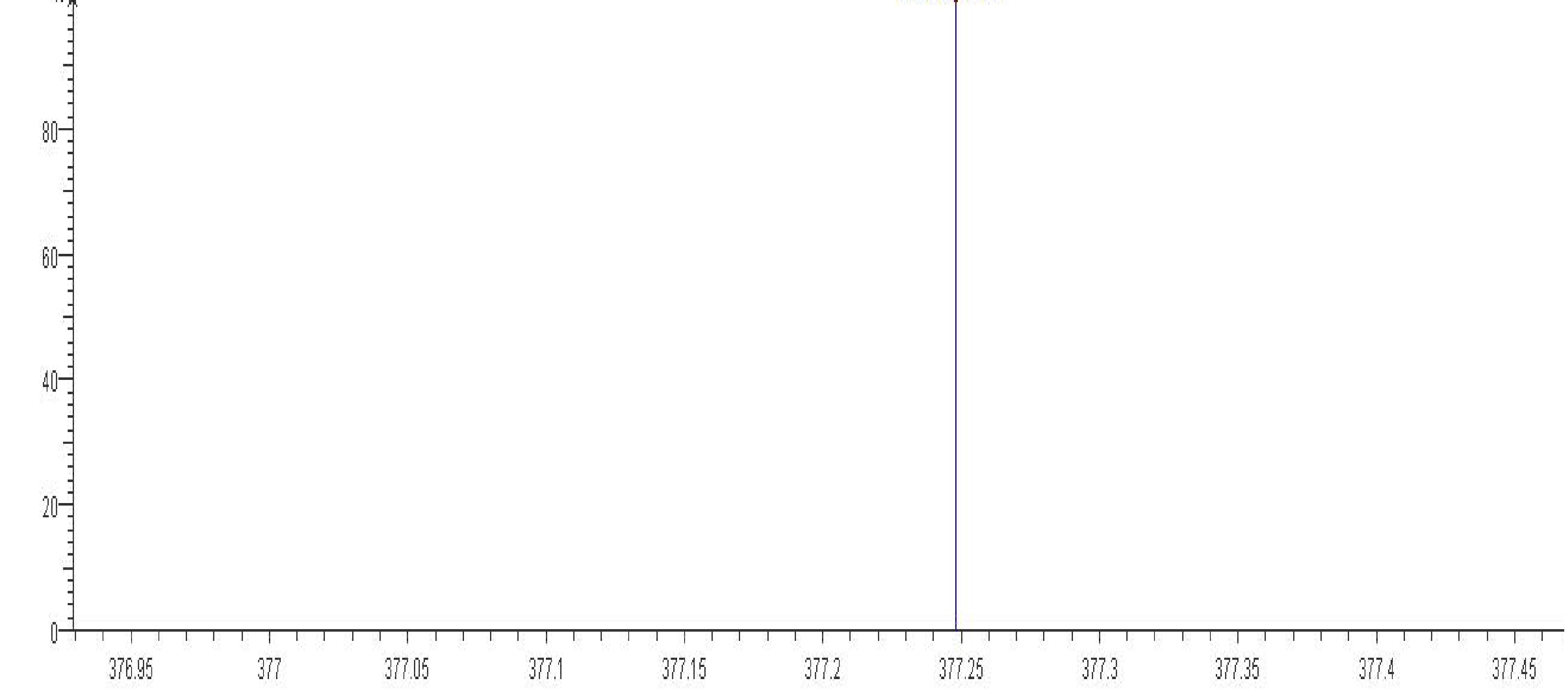

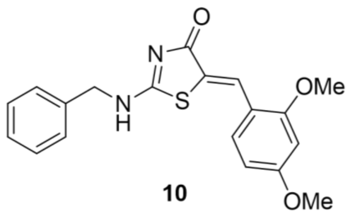

10

Figure S45. LRMS (ESI+) spectrum of compound **10**

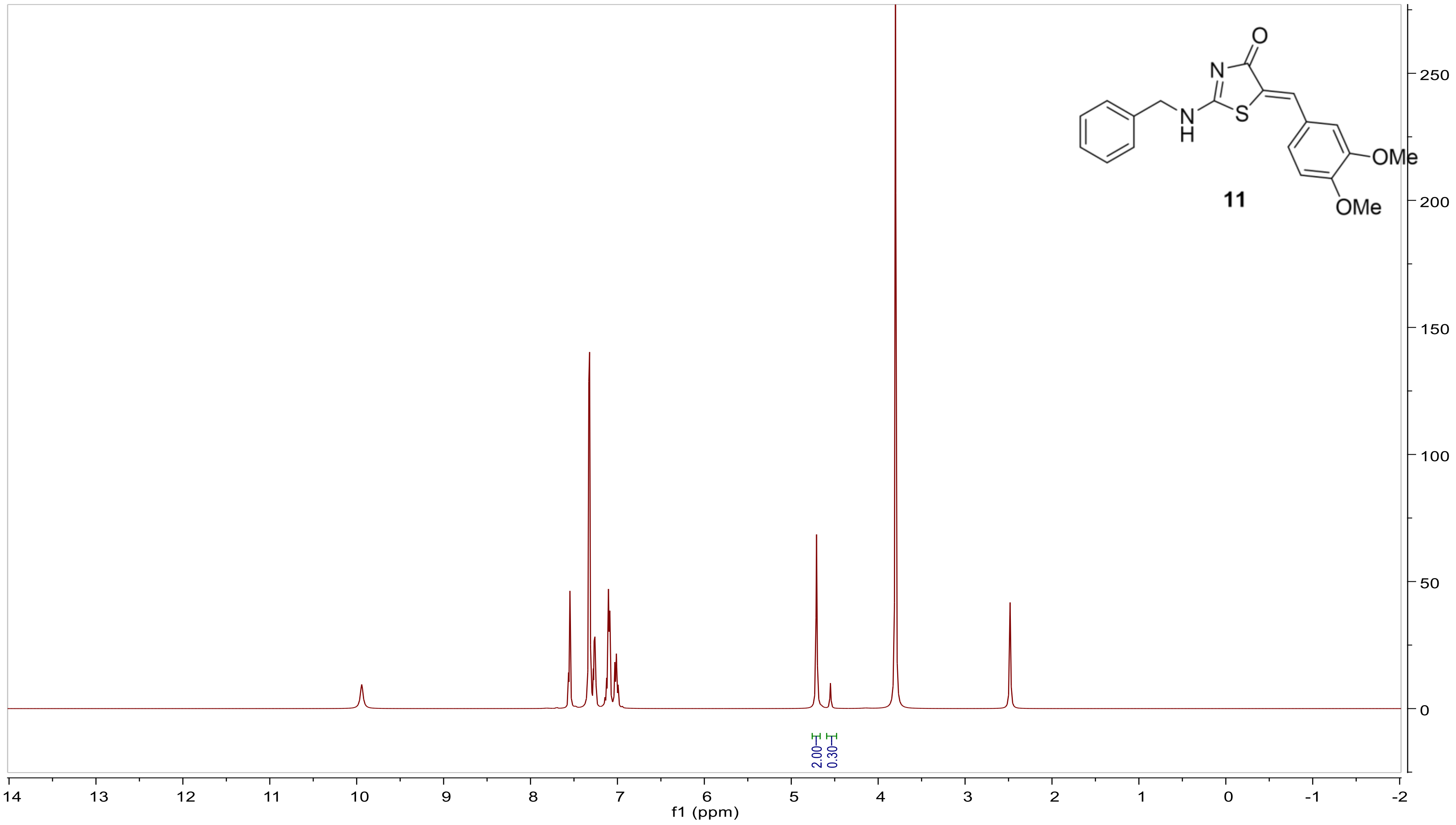

Figure S46. ^{1}H NMR spectrum of compound **11**

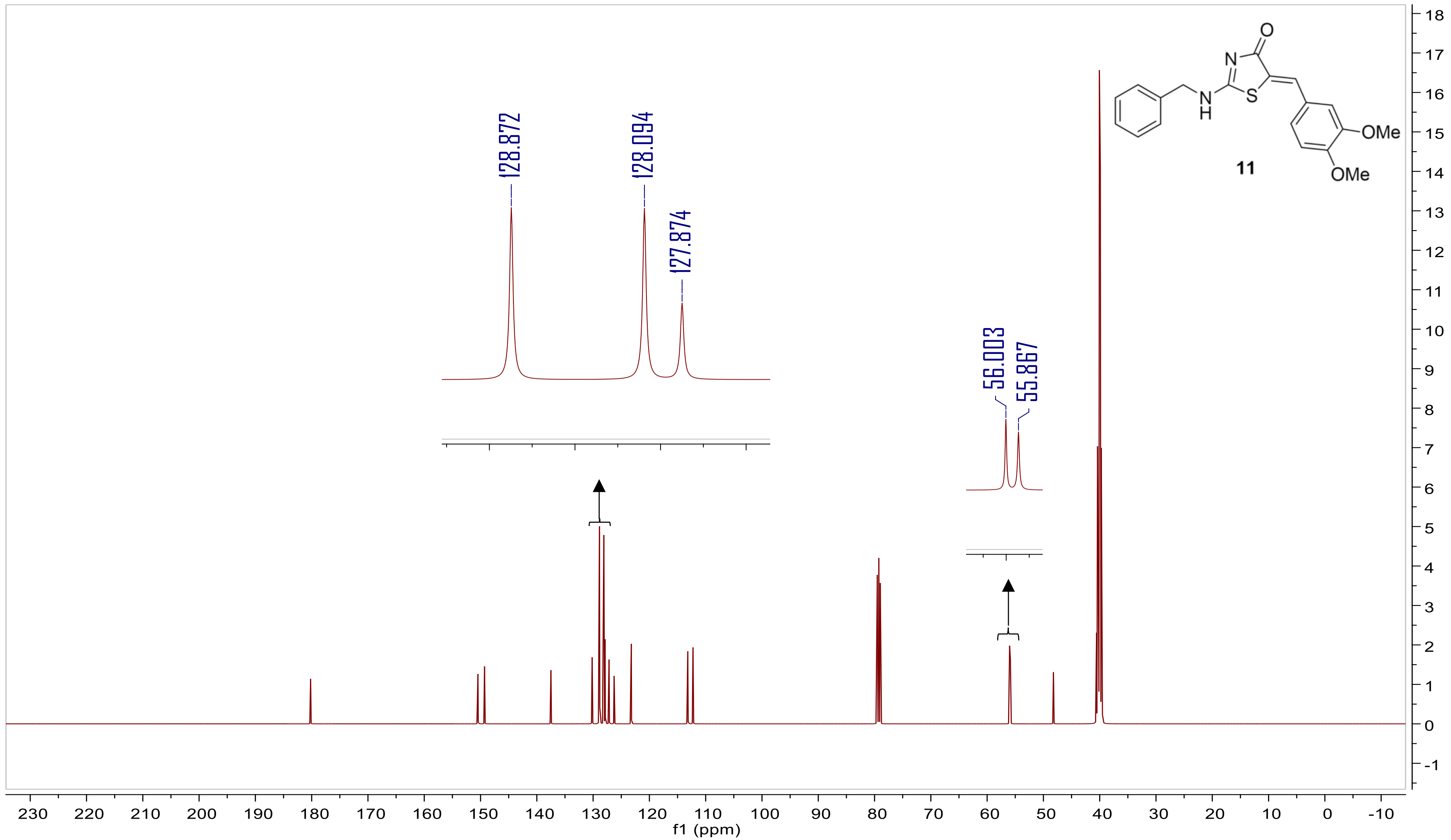

Figure S47. ^{13}C NMR spectrum of compound **11**

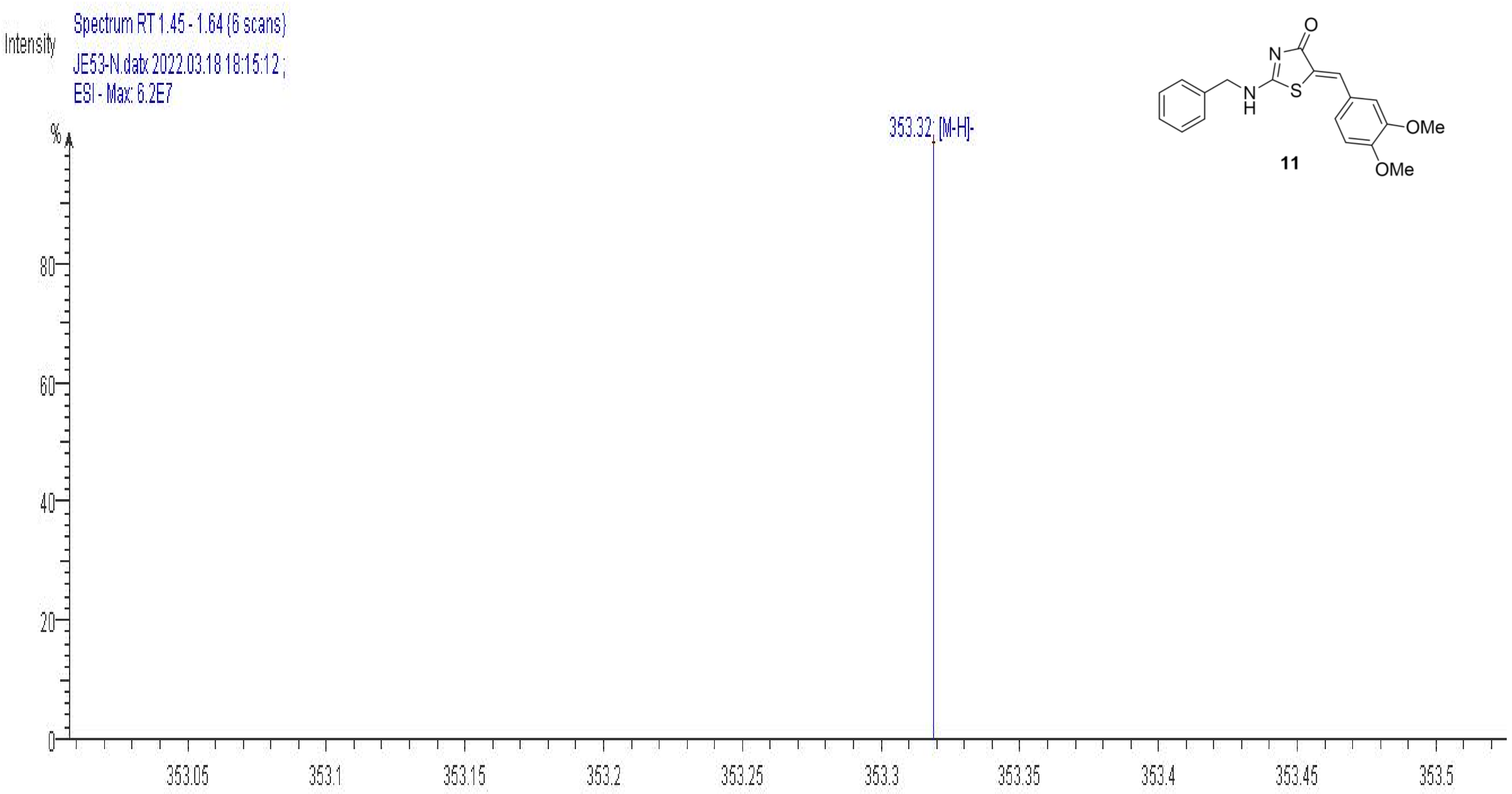

11

Figure S48. LRMS (ESI–) spectrum of compound **11**

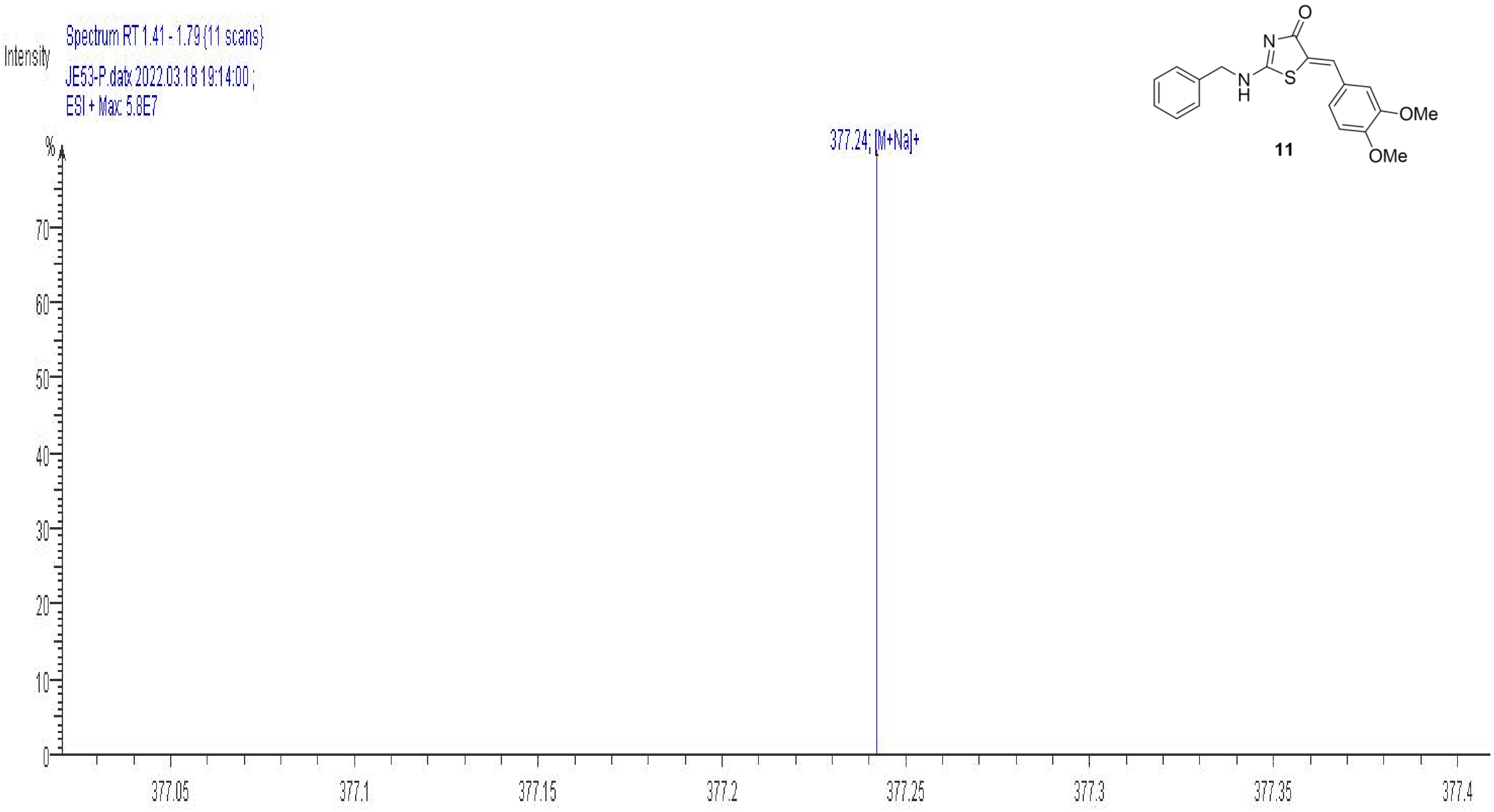

11

Figure S49. LRMS (ESI+) spectrum of compound **11**

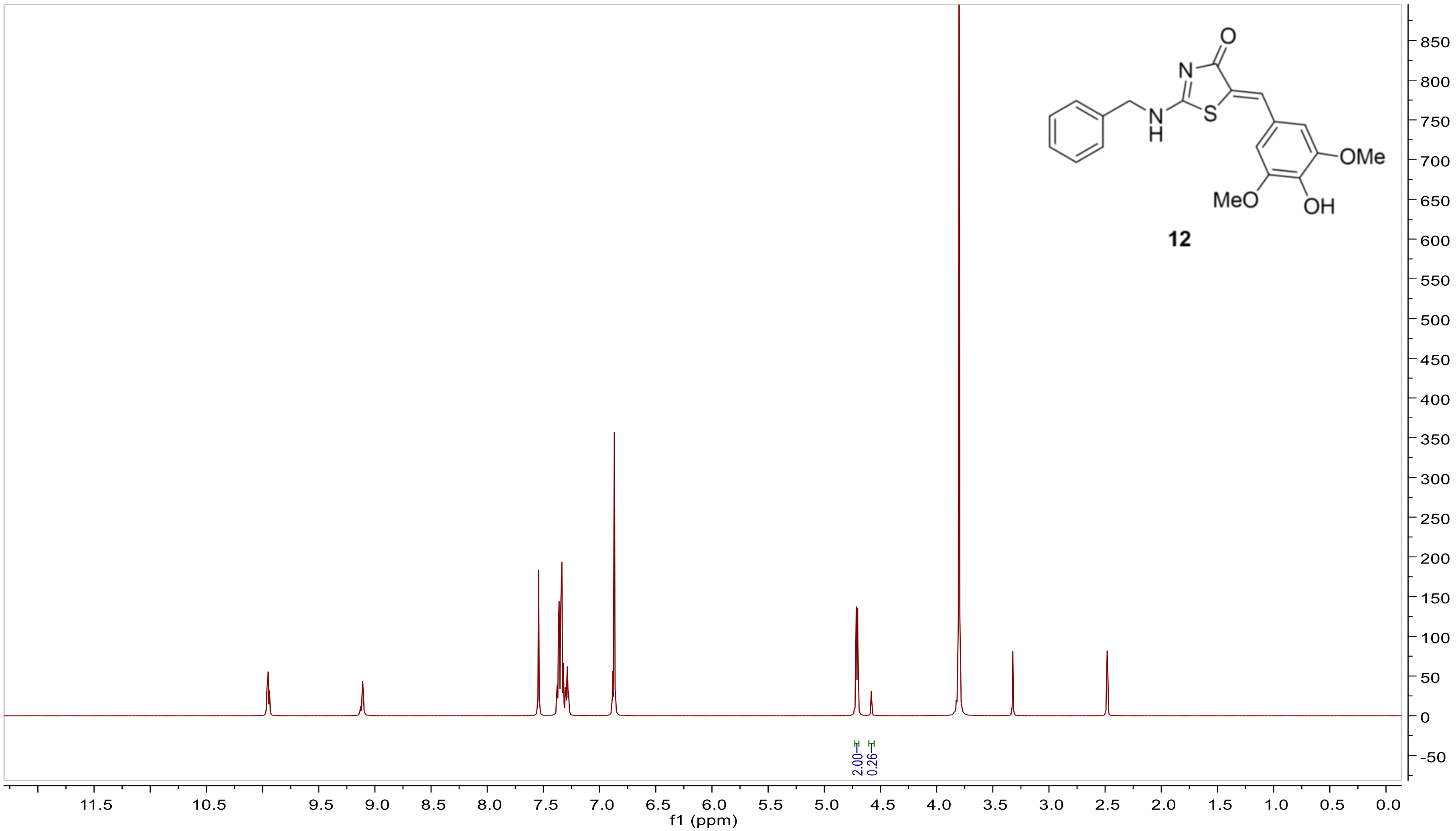

Figure S50. ¹H NMR spectrum of compound **12**

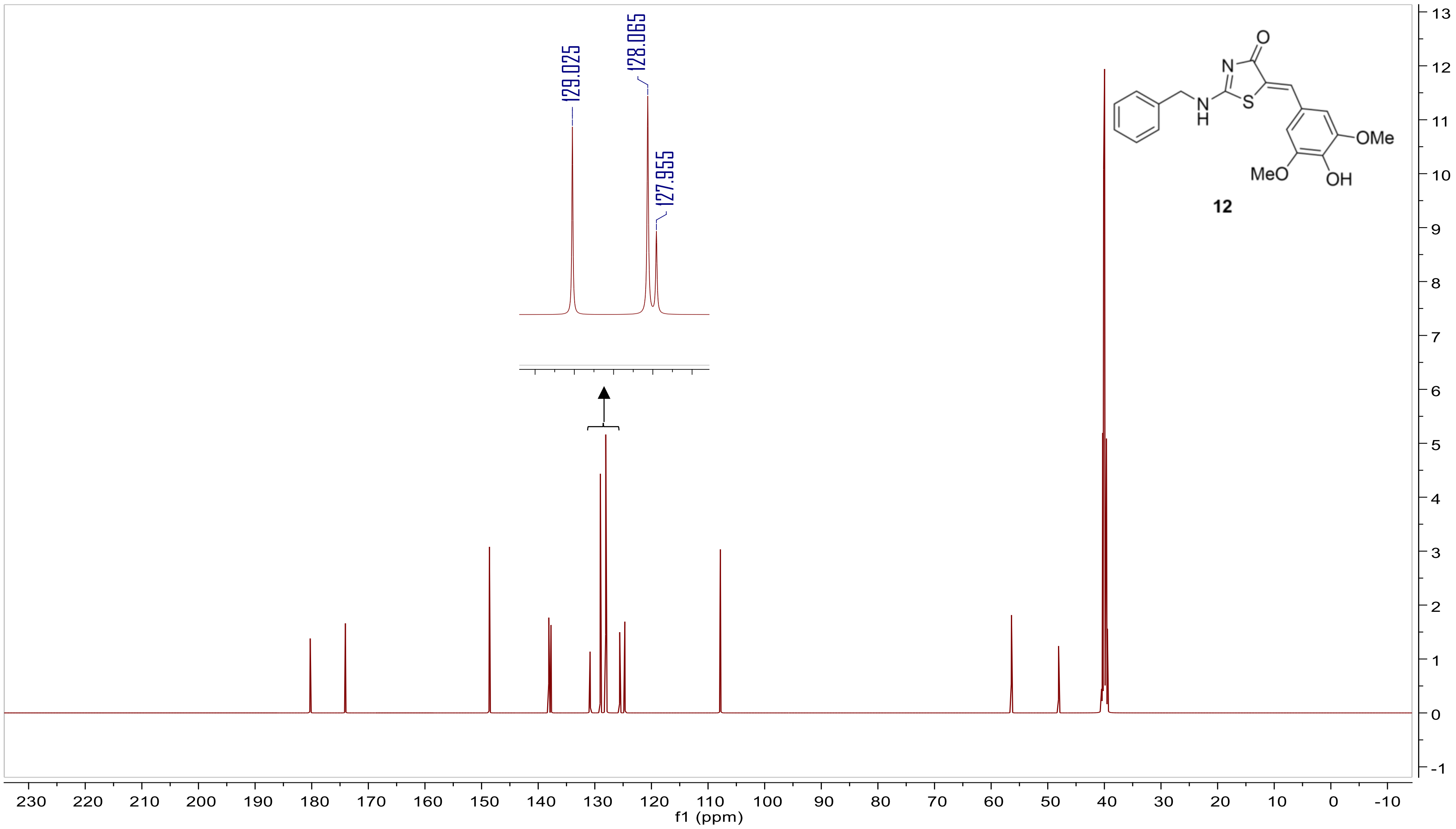

Figure S51. ^{13}C NMR spectrum of compound **12**

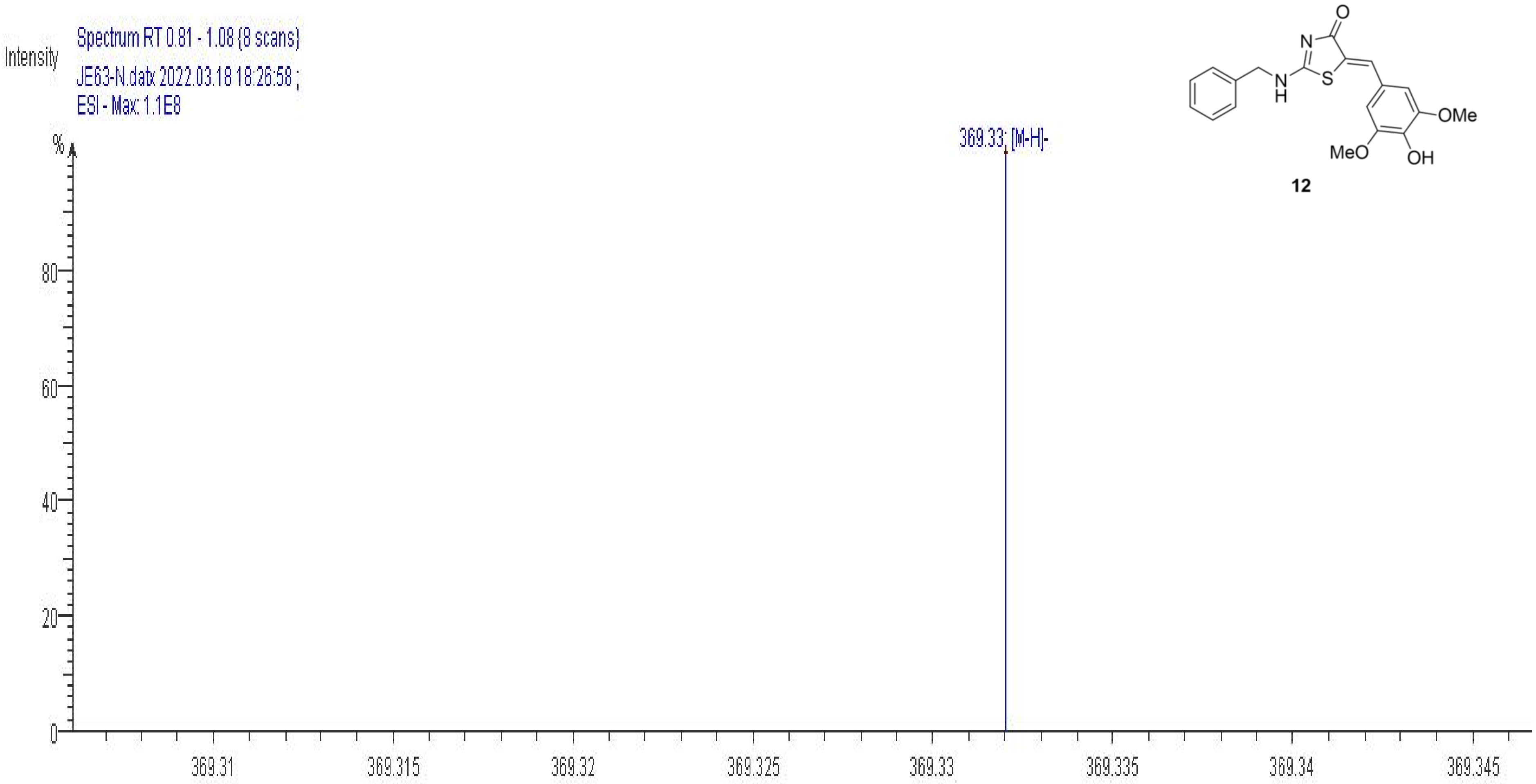

12

Figure S52. LRMS (ESI–) spectrum of compound **12**

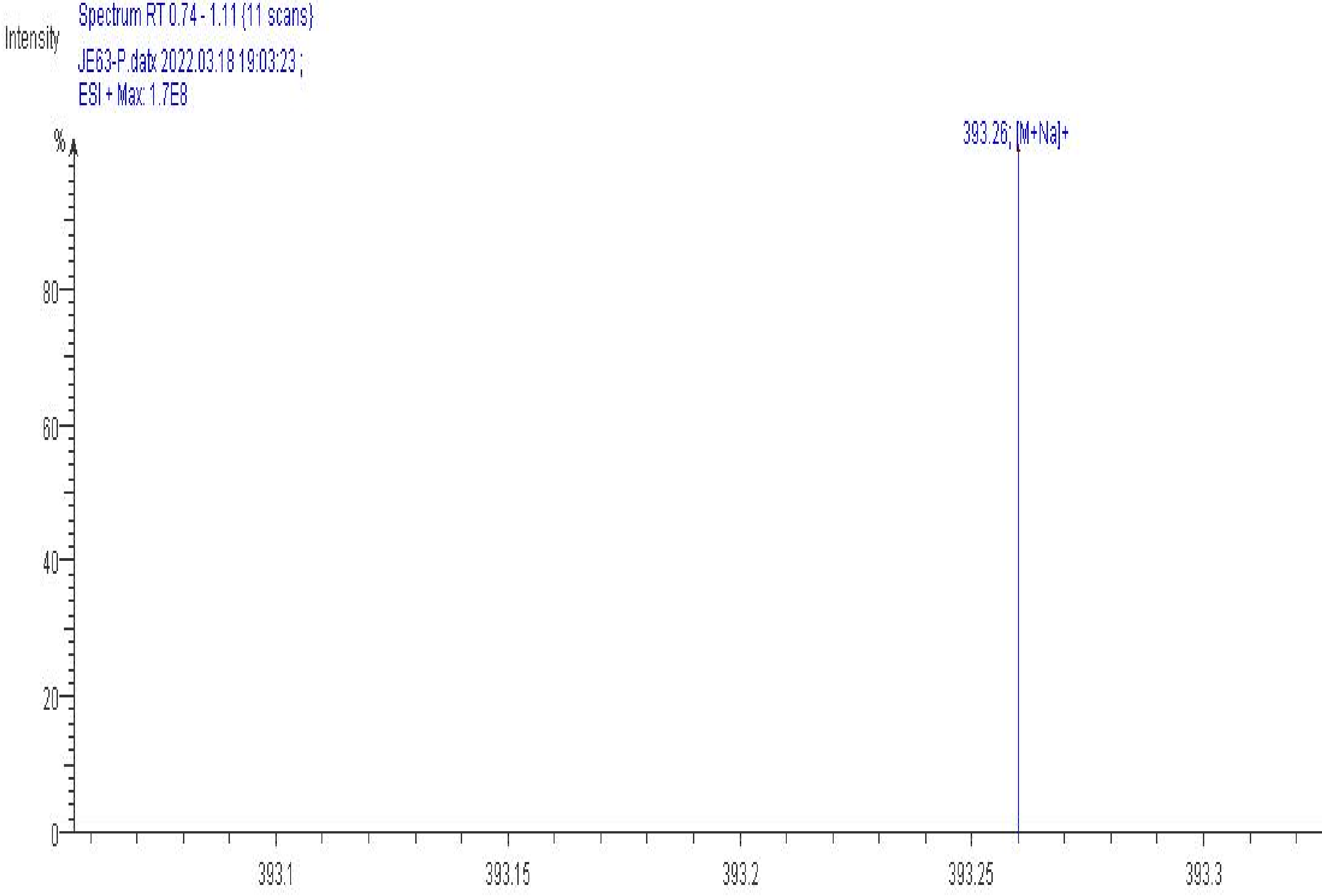

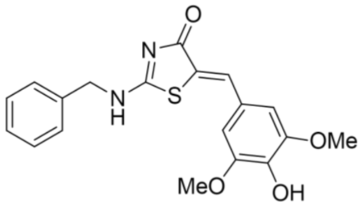

12

Figure S53. LRMS (ESI+) spectrum of compound **12**

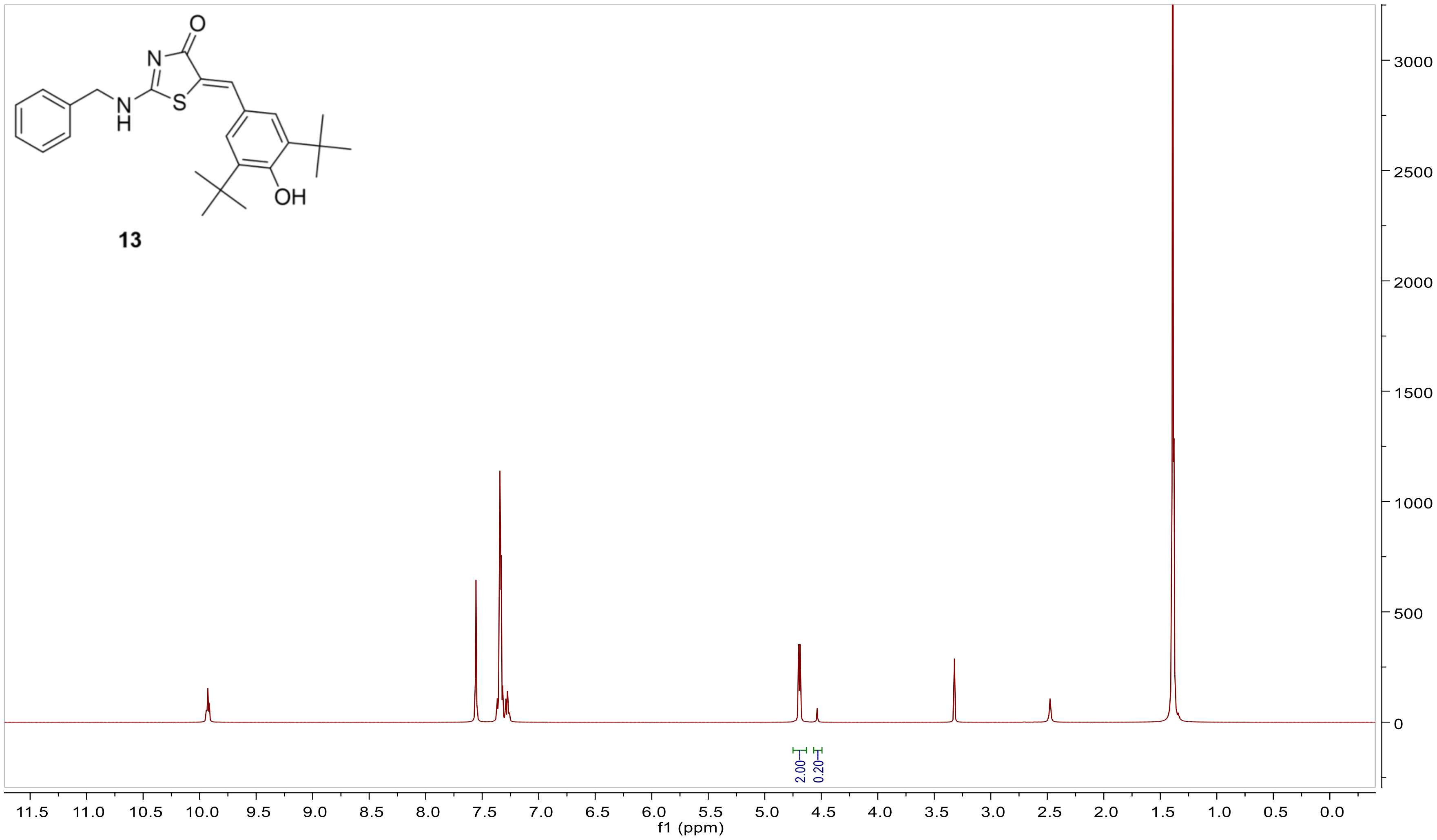

Figure S54. ^{1}H NMR spectrum of compound **13**

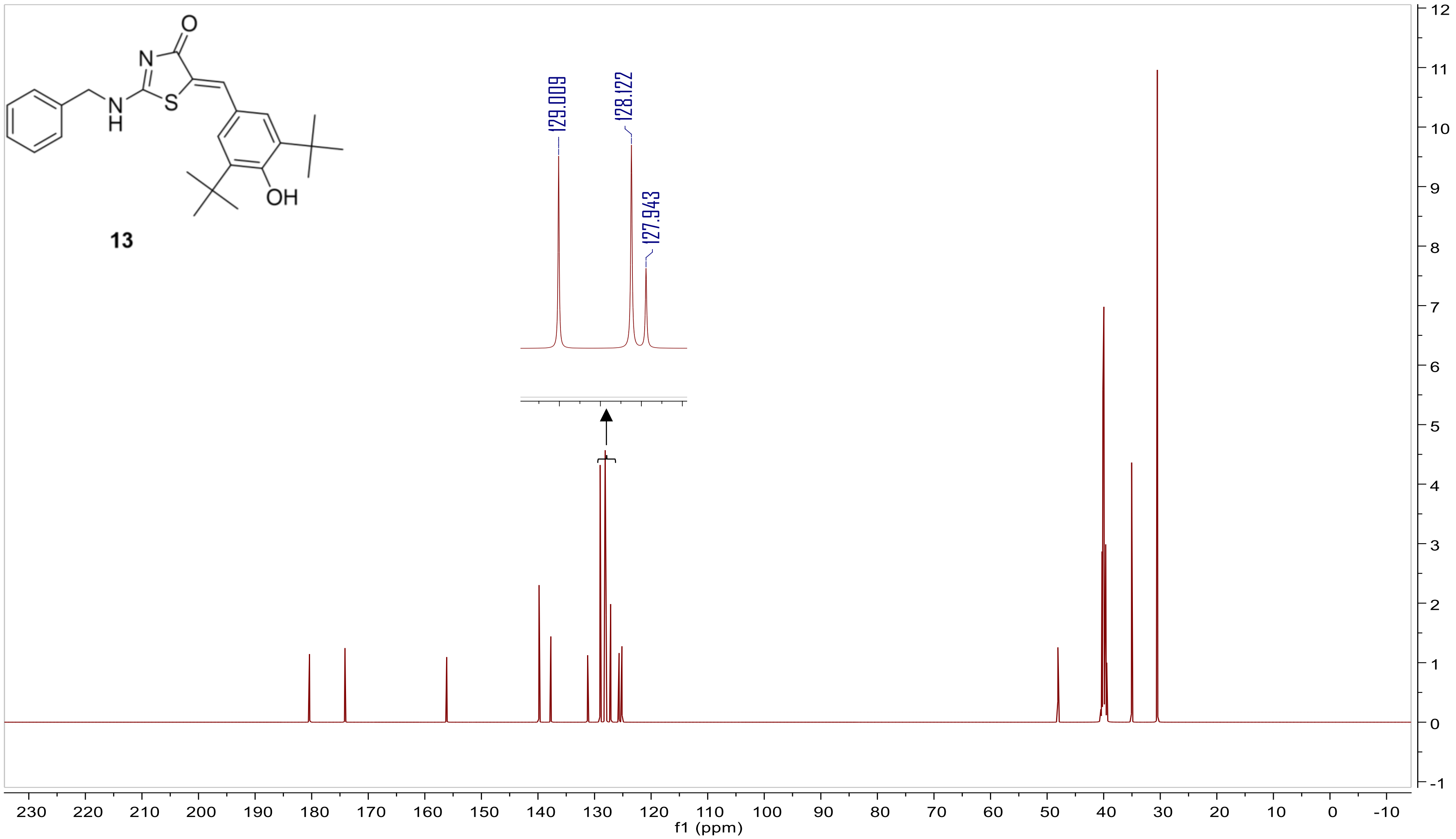

Figure S55. ^{13}C NMR spectrum of compound **13**

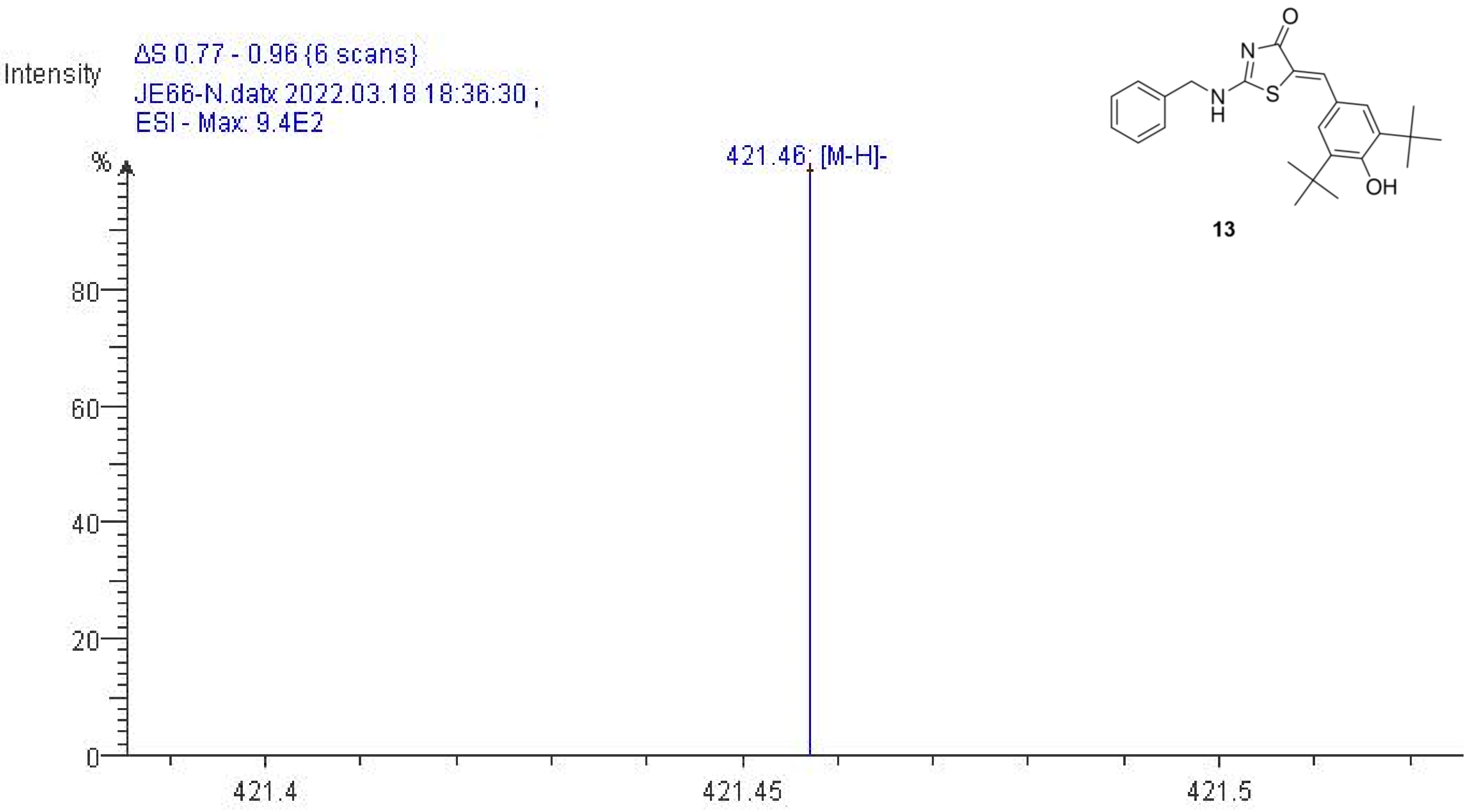

Figure S56. LRMS (ESI–) spectrum of compound **13**

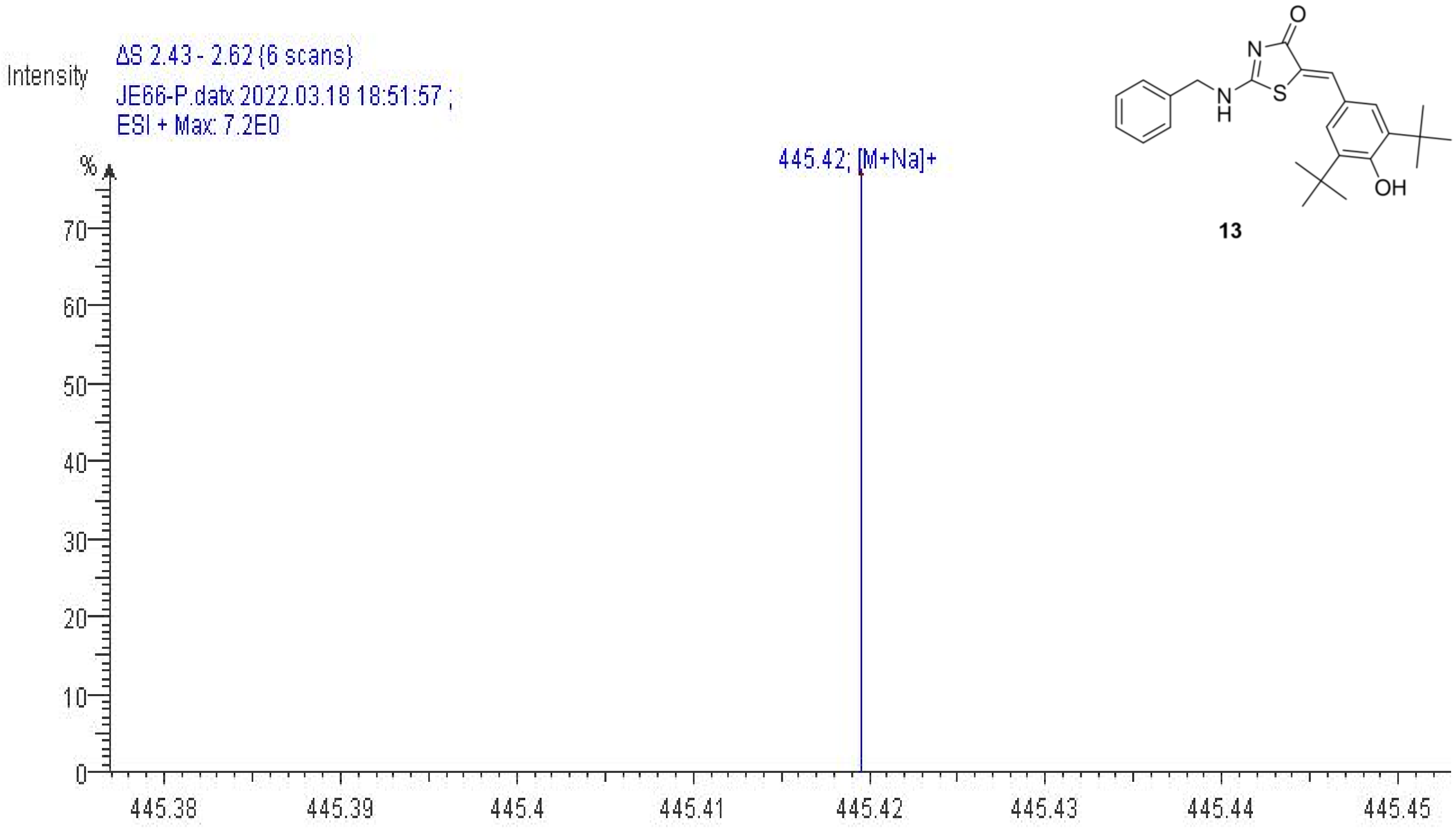

Figure S57. LRMS (ESI+) spectrum of compound **13**

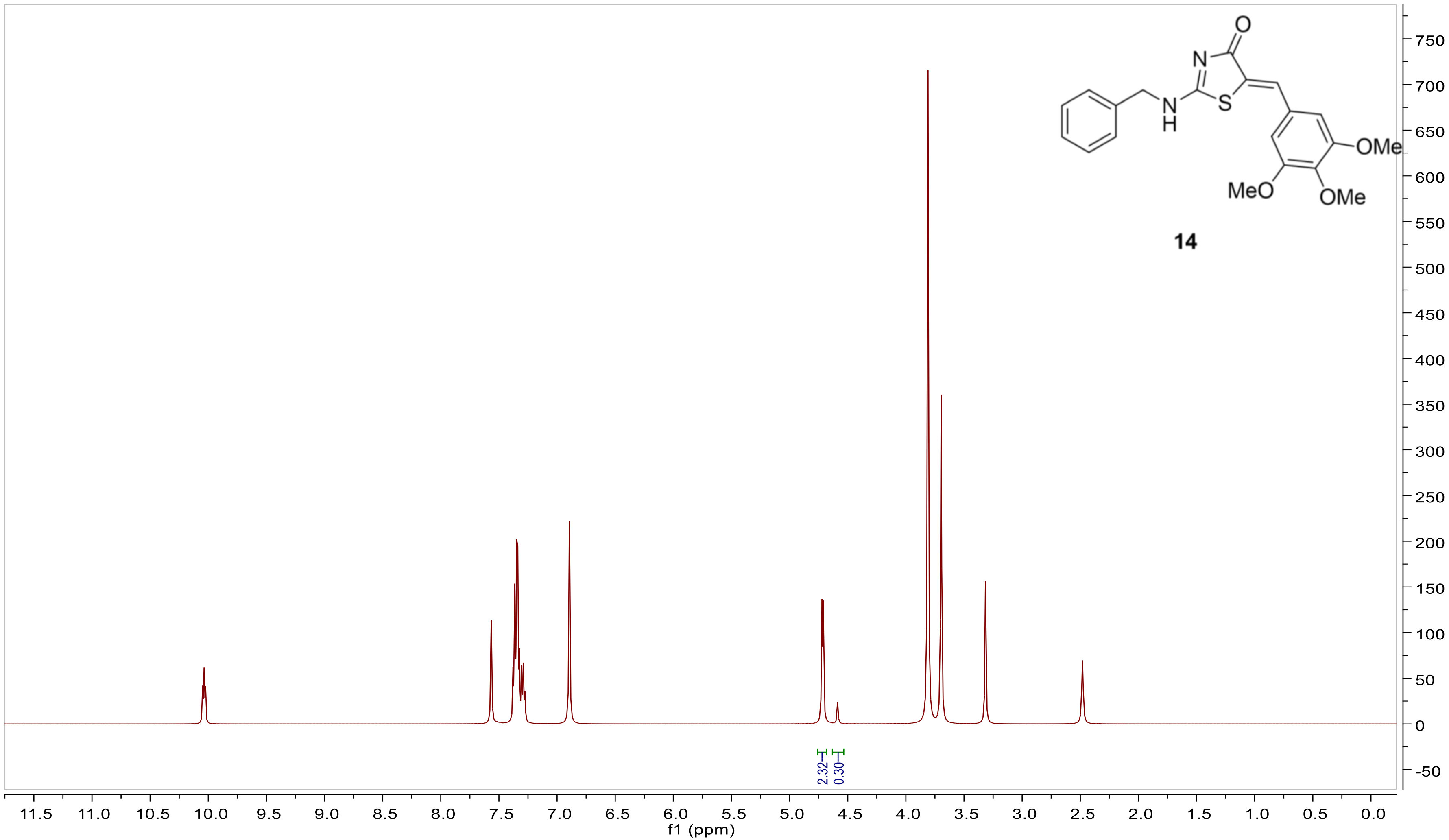

Figure S58. ^{1}H NMR spectrum of compound **14**

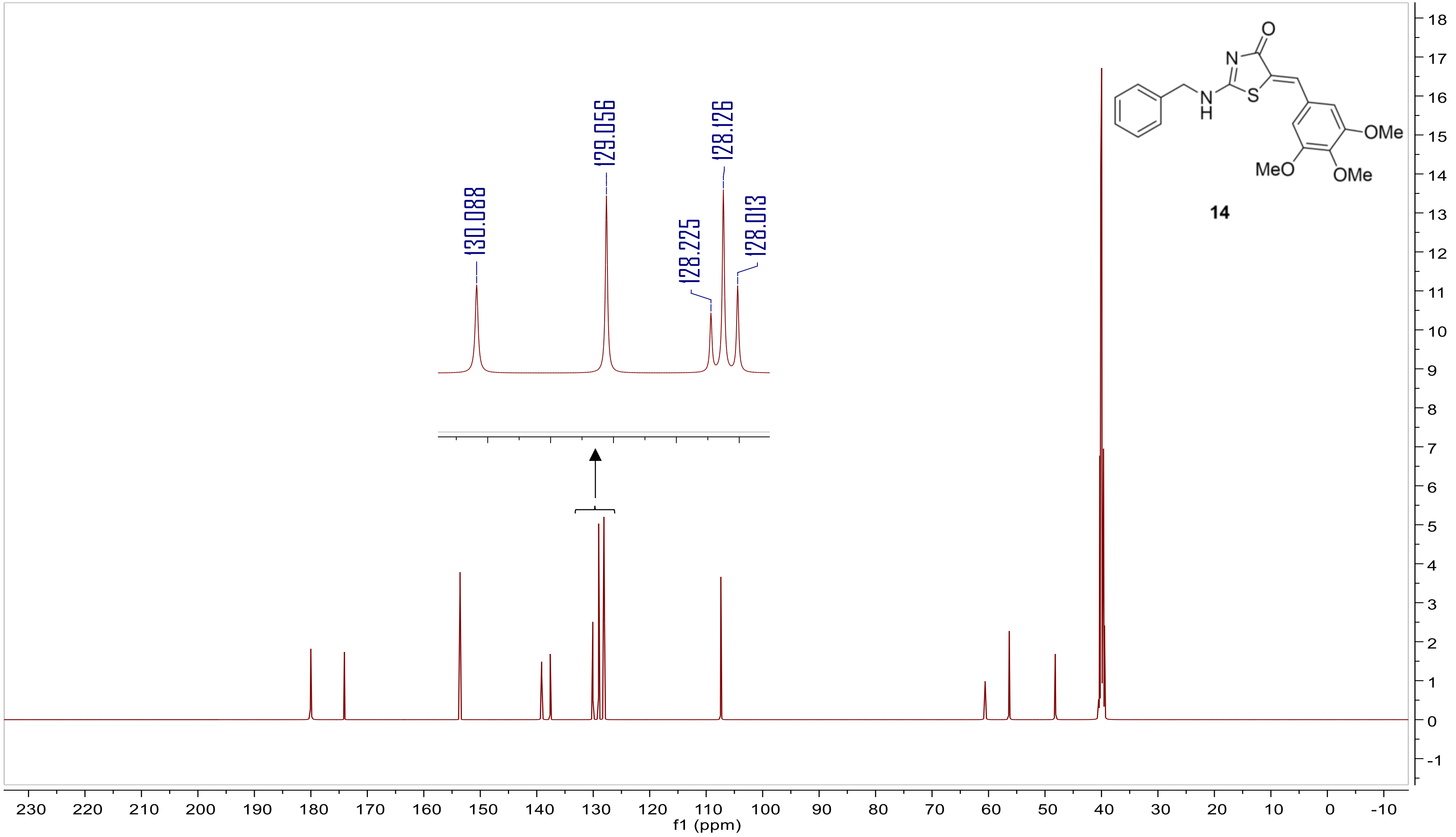

Figure S59. ^{13}C NMR spectrum of compound **14**

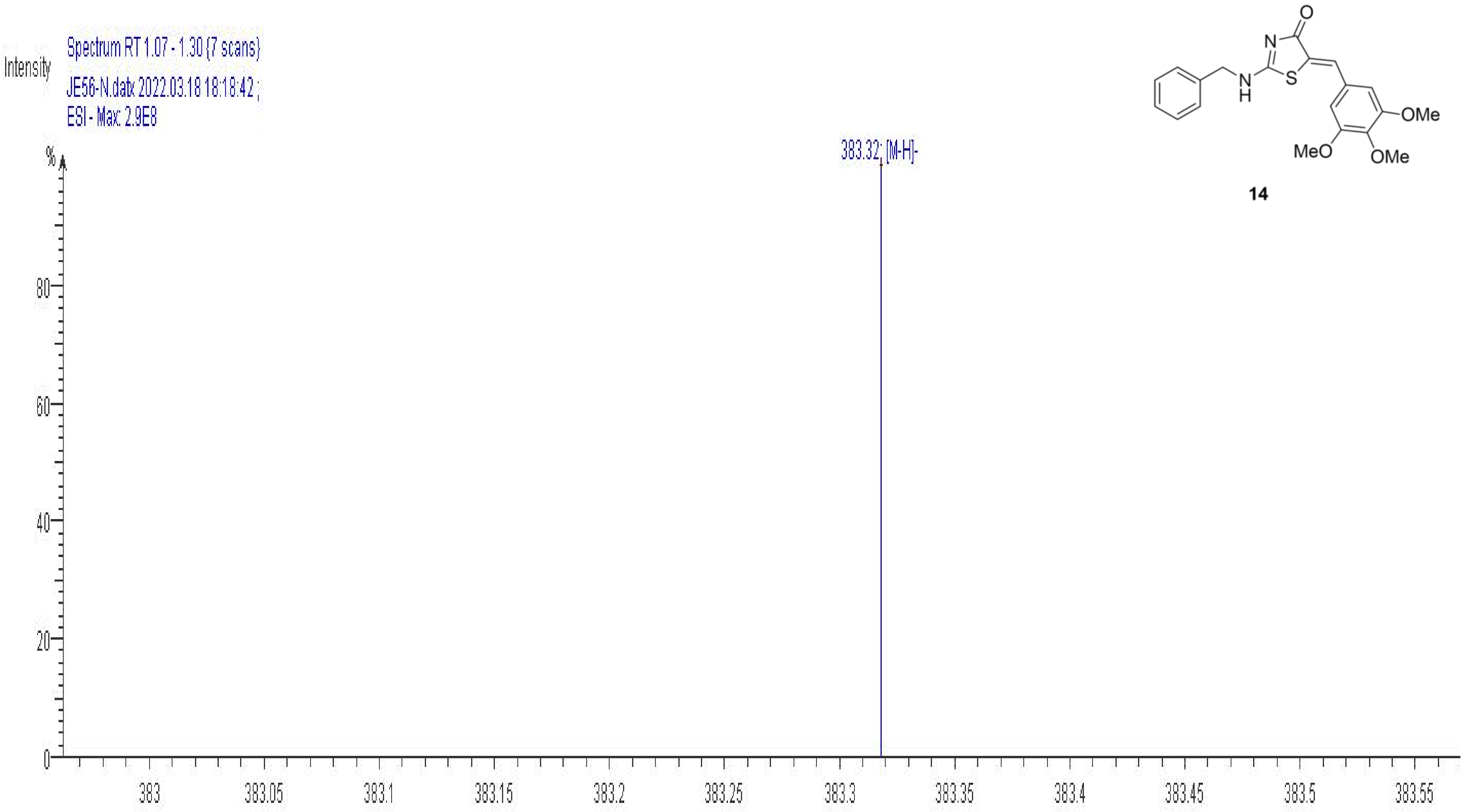

14

Figure S60. LRMS (ESI–) spectrum of compound **14**

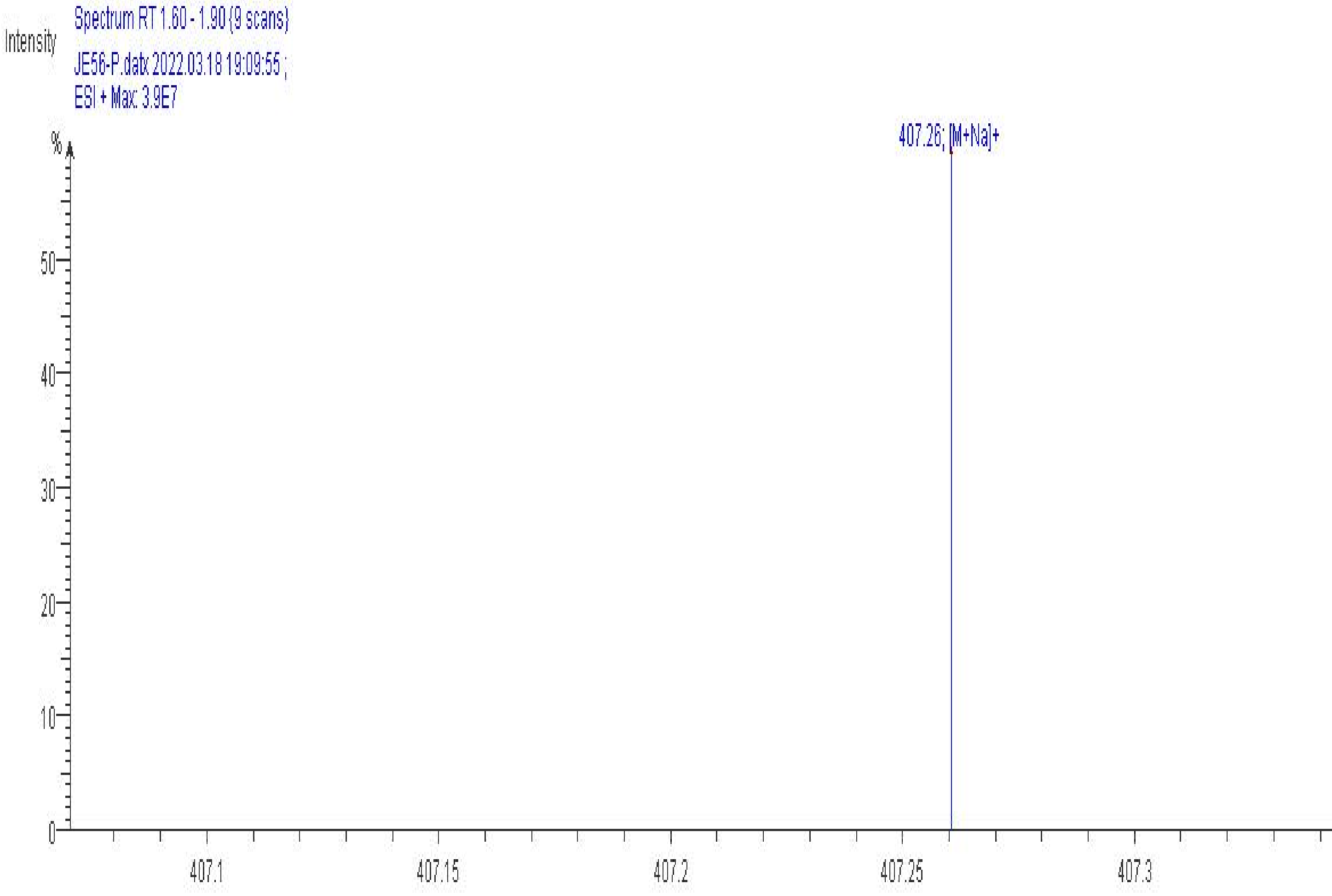

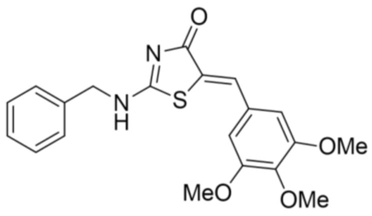

14

Figure S61. LRMS (ESI+) spectrum of compound **14**
